# Chromosomal inversion polymorphisms are widespread across the species ranges of rough periwinkles (*Littorina saxatilis* and *L. arcana*)

**DOI:** 10.1111/mec.17160

**Published:** 2023-10-16

**Authors:** James Reeve, Roger K. Butlin, Eva L. Koch, Sean Stankowski, Rui Faria

**Affiliations:** ^1^ Tjärnö Marine Laboratory University of Gothenburg Strömstad Sweden; ^2^ Ecology and Evolutionary Biology School of Biosciences University of Sheffield Sheffield UK; ^3^ Department of Zoology University of Cambridge Cambridge UK; ^4^ Institute of Science and Technology – Austria Klosterneuburg Austria; ^5^ Centro de Investigação em Biodiversidade e Recursos Genéticos InBIO Laboratório Associado Universidade do Porto Vairão Portugal; ^6^ BIOPOLIS Program in Genomics Biodiversity and Land Planning CIBIO Vairão Portugal

**Keywords:** ecological genetics, inversion, molecular evolution, molluscs, speciation

## Abstract

Inversions are thought to play a key role in adaptation and speciation, suppressing recombination between diverging populations. Genes influencing adaptive traits cluster in inversions, and changes in inversion frequencies are associated with environmental differences. However, in many organisms, it is unclear if inversions are geographically and taxonomically widespread. The intertidal snail, *Littorina saxatilis*, is one such example. Strong associations between putative polymorphic inversions and phenotypic differences have been demonstrated between two ecotypes of *L*. *saxatilis* in Sweden and inferred elsewhere, but no direct evidence for inversion polymorphism currently exists across the species range. Using whole genome data from 107 snails, most inversion polymorphisms were found to be widespread across the species range. The frequencies of some inversion arrangements were significantly different among ecotypes, suggesting a parallel adaptive role. Many inversions were also polymorphic in the sister species, *L*. *arcana*, hinting at an ancient origin.

## 1 INTRODUCTION

Inversions suppress recombination, allowing combinations of alleles to be maintained despite gene flow, which potentially plays a key role in local adaptation and speciation (Butlin, 2005; Faria, Johannesson, et al., 2019; Faria & Navarro, 2010; Hoffmann & Rieseberg, 2008; Jackson, 2011; Kirkpatrick & Barton, 2006; Wellenreuther & Bernatchez, 2018). Although inversions have been identified in numerous systems spanning the speciation continuum (Wellenreuther & Bernatchez, 2018), they are often polymorphic in one or both diverging populations, suggesting that they are under balancing selection (Durmaz et al., 2020; Faria, Johannesson, et al., 2019; Wellenreuther & Bernatchez, 2018). To understand the interplay between balancing and divergent selection within inversions, we need to track how they evolve, determining their origin and spread and the balance of evolutionary forces affecting them over time.

Empirical evidence for the role of inversions in divergence is often limited to small geographical areas, which raises the question: are the same inverted regions driving local adaptation across a species range? Some studies have explored the adaptive role of inversions across global distributions. In some cases, inversion frequencies change across broad biogeographic clines, such as with latitude in *Drosophila melanogaster* (Kapun & Flatt, 2019) or precipitation in several malaria‐harbouring mosquito species (Ayala et al., 2014, 2017). In other (although non‐mutually exclusive) cases, inversions are involved in local adaptation, leading to parallel phenotypic evolution across sites with similar environmental contrasts (Westram et al., 2022). Examples include three inversions in the three‐spine stickleback that differentiate freshwater and marine populations (Jones et al., 2012), four inversions linked with migratory behaviour in Atlantic cod populations (Matschiner et al., 2022) and 13 inversions associated with changes between forest and prairie habitats in deer mice (Harringmeyer & Hoekstra, 2022). These parallel patterns strongly support inversions' role in local adaptation and emphasize how characterizing the global distribution of inversions helps to understand the genetic basis of adaptation.

The rough periwinkle (*Littorina saxatilis* Olivi, 1792) is a useful study system for understanding the role of inversions in adaptation and speciation. *L. saxatilis* is a phenotypically diverse intertidal snail that primarily inhabits rocky seashores across the North Atlantic (Reid, 1996, pp. 324–331). Recently, 18 clusters of loci in linkage disequilibrium have been found within the species, which are indications of polymorphic chromosomal inversions (Faria, Chaube, et al., 2019; Westram et al., 2021). Some of these putative inversions contain loci influencing adaptive traits that differentiate two ecotypes (Koch et al., 2021, 2022): a *crab* ecotype resistant to predation by shore crabs (Boulding et al., 2017; Janson, 1982; Johannesson, 1986) and a *wave* ecotype resistant to dislodgment by waves (Larsson, 2021; Le Pennec et al., 2017). These polymorphic inversions and their associations with ecotypes are repeated across multiple nearby sites in Sweden (Westram et al., 2021). Strong genetic differentiation between ecotypes occurs at genomic regions corresponding to some Swedish inversions, suggesting that similar associations exist in the United Kingdom, France and Spain (Kess & Boulding, 2019; Morales et al., 2019). However, while a signal of *crab*–*wave* divergence has been inferred across Europe, there is currently no direct evidence that the inversions detected in Sweden are polymorphic across the species range.

The species range of *L*. *saxatilis* covers many habitats and overlaps substantially with two closely‐related species (*L*. *arcana* and *L*. *compressa;* all three species ranges overlap from Brittany to the Barents Sea). Morphological studies over the last two centuries have proposed numerous species names and taxonomic subgroupings for *L*. *saxatilis* (Reid, 1996, pp. 278–292). Reid (1996, pp. 305–318) summarized this variation as four ecotypes: *crab* (*moderate* sensu Reid), *wave*, *brackish* and *barnacle*, that occur within ovoviviparous *L*. *saxatilis* and also its egg‐laying relatives, *L*. *arcana* and *L*. *compressa* (pp. 248–278). *L*. *saxatilis* and *L*. *arcana* are considered sister species with near‐complete reproductive isolation (Stankowski et al., 2020). There is also increasing evidence of a strong phylogeographic break in *L*. *saxatilis* around the Bay of Biscay, separating populations in the Iberian Peninsula from those in the North (Doellman et al., 2011; Morales et al., 2019; Panova et al., 2011; Tirado et al., 2016). By identifying inversions in other ecotypes, species and geographic regions, we can better contextualize their adaptive role in *L*. *saxatilis* and their taxonomic spread.

Our current knowledge of the *crab* and *wave* ecotypes can help us infer the adaptive role of inversions in other ecotypes facing similar environmental pressures. The *barnacle* ecotype is found on very exposed shores; thus, any inversion arrangement common in *wave* snails is also expected to be common in *barnacle* snails. Similarly, *L*. *arcana* typically inhabits moderately exposed shores (Reid, 1996, p. 274); therefore, if they share inversions with *L*. *saxatilis*, it is more likely they carry *wave* arrangements. However, this prediction assumes that adaptation is only directed by wave exposure and predation, an assumption that may not always hold (Morales et al., 2019).

To clarify the adaptive role of *L*. *saxatilis* inversions, we investigated the distribution of each inversion polymorphism across the entire species range. Specifically, we aimed to: (i) find whether inversions that were identified in Sweden are polymorphic across the species range; (ii) determine if the inversions previously associated with *crab–wave* divergence also differentiate ecotypes consistently throughout the species range and (iii) investigate if these inversion polymorphisms are shared with a sister species (*L*. *arcana*), which would suggest an ancient origin. Studying the geographic and taxonomic distribution of inversions should provide a broader perspective on their roles in ecotype formation and speciation.

## 2 METHODS

### 2.1 Sample collection

**FIGURE 1:**
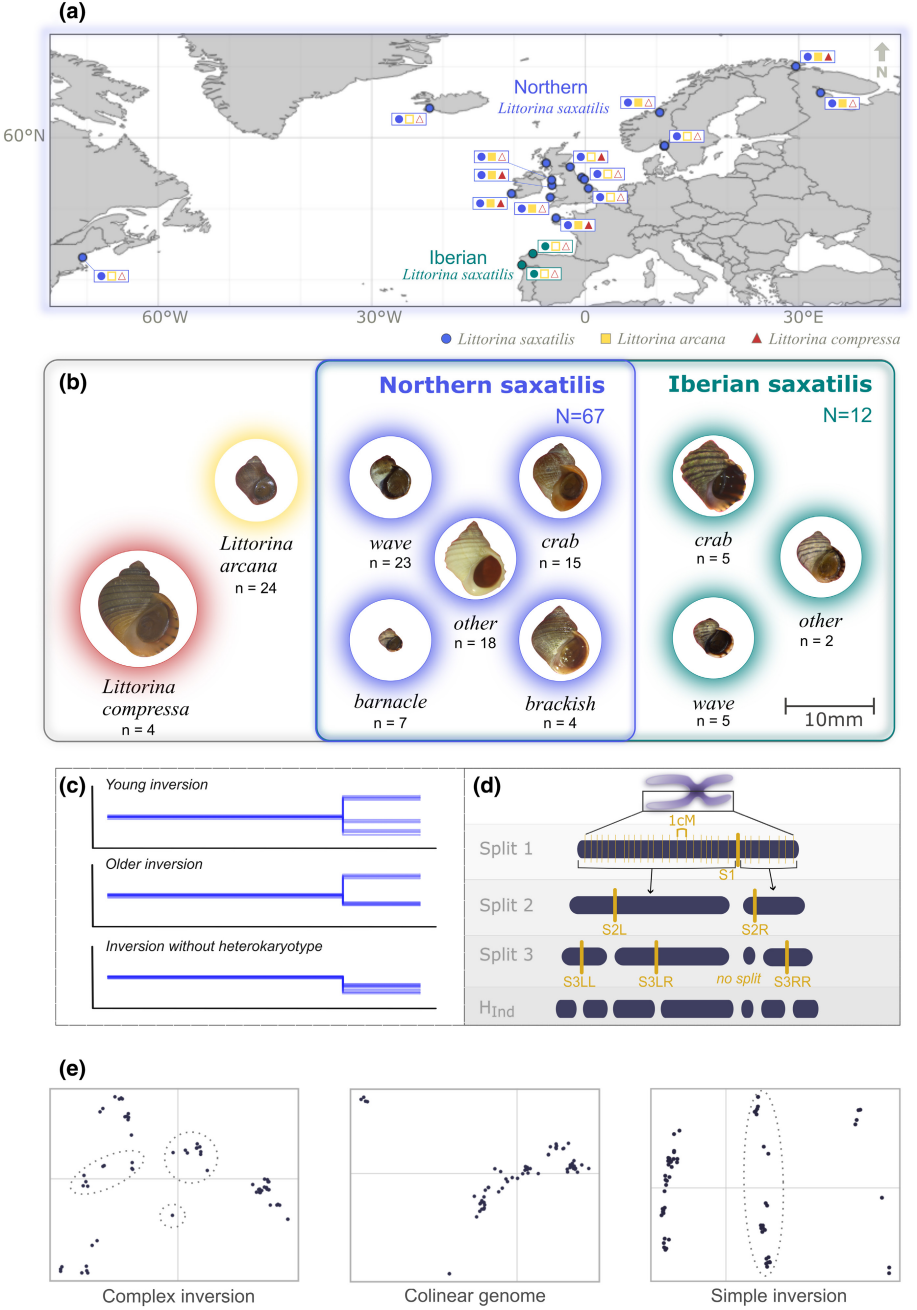
Distribution of samples included in this study and schematics of detection methods. (a) Map of sampling locations. Solid symbols represent the presence of *Littorina saxatilis* (circle), *L*. *arcana* (square) and *L*. *compressa* (triangle) at each site, according to Reid (1996). Hollow symbols represent absence. The base map was produced in QGIS using Natural Earth countries data with a 1:50 m resolution (crs = WGS 84; https://www.naturalearthdata.com/downloads/). (b) Shell photos of each species, genetic group and ecotype. All four *L*. *saxatilis* ecotypes (Reid, 1996, pp. 305–318) are shown, with only *crab* and *wave* existing in Iberia. An additional photo is included for both the Iberian and Northern saxatilis groups of a snail that does not fit an established ecotype. For Northern saxatilis, the *crab*, *wave* and *brackish* photos were taken from snails collected in Sweden; the *barnacle* and *L*. *arcana* snails were from the North‐East coast of England; *L*. *compressa* was from Northern Wales; and the ‘other’ snail was from Iceland. For the Iberian saxatilis, the *crab* and *wave* photos were taken from snails collected from Centinela, while the ‘other’ snail was from near Burela. (c and d) Schematics of the heterozygosity split approach. (c) Hypothetical results for three different patterns that could indicate inversions. (d) Diagram of how split positions are determined. Each split is represented by a thick yellow line. Each linkage group is split three times for an individual. The final row shows the segments that are used to calculate *H*_Ind_. (e) Example PCA plots showing patterns of a complex double inversion (LGC6.1/2), a colinear segment of the genome (LG8) and a simple inversion (LGC1.2). Heterokaryotype clusters have been circled.

The dataset used for this study was gathered for a phylogenetic study of the *L*. *saxatilis* species complex (Stankowski, Zagrodzka, Galindo, et al., 2023; Stankowski, Zagrodzka, Garlovsky, et al., 2023). Snails were collected between 2014 and 2020 by several different collectors from 18 locations across the North Atlantic (Figure 1a; Table S1). Where possible, all ecotypes and species present were sampled. However, not all locations had habitats or shell characteristics that were typical of the recognized ecotypes described by Reid (1996, pp. 305–318). In such cases, these individuals were classified as ‘other’. The collection site details and full list of collectors are available in the Supporting Information (Table S1).

Only reproductively mature individuals were sequenced. Maturity was determined by examining the reproductive anatomy. *L*. *saxatilis* and *L*. *arcana* were also distinguished by female reproductive anatomy. Males could not be distinguished by morphology; hence, only females were analysed in locations where these species overlapped.

Samples were split into three genetic groups for analysis: Northern saxatilis, Iberian saxatilis and *L*. *arcana* (Figure 1). These were chosen based on known genetic differences to reduce the impact of geographic isolation (Northern vs. Iberian; Doellman et al., 2011; Morales et al., 2019; Panova et al., 2014; Stankowski, Zagrodzka, Galindo, et al., 2023; Tirado et al., 2016) and reproductive isolation (*L*. *saxatilis* vs. *L*. *arcana*; Stankowski et al., 2020) while still maintaining a sufficient sample size to identify polymorphic inversions. *L*. *compressa* was used as an outgroup and not included in inversion‐detection analyses due to the low sample size (four). One *L*. *saxatilis* (IMI_6_2) was excluded outright due to missing collection information. Sample sizes are presented in Table S1.

### 2.2 Sequencing and SNP calling

DNA was extracted from a small piece of foot tissue from each snail using a CTAB protocol (Panova et al., 2016). These DNA samples were sent to Edinburgh Genomics (University of Edinburgh) for library preparation using a 350‐bp insert TrueSeq DNA Nano gel‐free approach and sequenced on an Illumina HiSeqX machine with 150‐bp pair‐end reads and 15X target coverage. Reads were aligned, and variants were called following the pipeline in Stankowski et al. (2020). Briefly, reads were trimmed and then mapped to the *L*. *saxatilis* genome assembly (Westram et al., 2018) using BWAmem (Li & Durbin, 2009), followed by variant calling using GATK4 (McKenna et al., 2010). Called SNPs were then filtered to remove those with multiple alleles (>2), low mapping quality (Q < 30), minor allele frequencies (maf) below 0.05 and extreme depth of coverage (5 > AD > 35). Only SNPs mapped onto a contig on the *L. saxatilis* linkage map were retained.

### 2.3 Updating inversion positions on the linkage map

SNP genotypes were assigned by contig onto a new version of the *L*. *saxatilis* linkage map, which accounts for map compression within inversions (see Supporting Information). This combines a map from a *crab* ecotype family (Westram et al., 2018, supp. mat.) with an F2 *crab–wave* map (Koch et al., 2021). This consensus map was filtered to remove any SNP markers within a contig that mapped to a different linkage group or were located >2 cM from the average map position of that contig. A new average map position was then assigned to each contig. Linkage groups (LG) 10 and 14 retained positions from the *crab* map because few informative markers were found on the *crab–wave* map. Our WGS SNPs did not match the markers used for map construction; thus, the average map position of each contig was assigned to SNPs in this dataset. Published positions of the inversion boundaries previously detected in this species (Hearn et al., 2022 for LG12; Westram et al., 2021 for others) were transferred to the new map using contigs that were previously mapped to inversion boundaries. Conservatively, the widest distance between inversion boundary positions was adopted.

### 2.4 Inversion detection approaches

Inversions were detected in each genetic group with two complementary approaches: variation in average observed heterozygosity (‘heterozygosity splits’) and principal component analysis (PCA). Approaches used elsewhere, such as *LDna* (Kemppainen et al., 2015), *l ocal_PCA* (Li & Ralph, 2019), or *asaph* (Nowling et al., 2020), could not be used for our data due to the lack of contiguity of the reference genome and the strong population structure across the species range. For consistency between our approaches, we used contigs in 1 cM non‐overlapping windows (see below), chosen to give sufficient SNPs for informative PCA results. Visual inspection was used to determine if the positions of inversions were consistent between approaches and genetic groups.

### 2.5 Inversion detection approach 1: heterozygosity splits

Inversions were identified by scanning each linkage group for significant shifts (‘splits’) in average individual heterozygosity (i.e. the proportion of SNPs in a genomic window that are heterozygous) among snails in each genetic group (i.e. Northern saxatilis, Iberian saxatilis and *L*. *arcana*). Heterozygosity is expected to differ among inversion karyotypes and to differ from regions outside the inversion (colinear genome). Young inverted homokaryotypes usually have reduced heterozygosity as they recently expanded from a single mutated haplotype. As they get older, inverted homokaryotypes accumulate genetic diversity from mutation and gene flux. Heterokaryotypes for older inversions have increased heterozygosity as they contain two isolated haplotypes that are likely to be differentiated at multiple sites. Finally, non‐inverted homokaryotypes are expected to have levels of heterozygosity similar to or slightly lower than the genetic background, as the inversion reduces effective population size (in proportion to inversion frequency) by acting as a localized reproductive barrier for part of the genome. However, gene flux and selection can distort these expectations. By comparing heterozygosity along the genome among individuals, an inversion can be identified by a cluster of splits in average individual heterozygosity that groups snails into two or three sets, at least one of which differs from the background (Figure 1c).

Splits in average individual heterozygosity (*H*_Ind_) were identified by dividing each linkage group into blocks of similar heterozygosity. Specifically, we designed a hierarchical split‐function (Figure 1d). For each individual, *H*_Ind_ was calculated on either side of potential splits and distributed every 1 cM along each linkage group. A model was fitted to the data with a single mean *H*_Ind_ and beta‐binomial distribution, with a fitted dispersion parameter *ρ*. Models were then compared for all possible splits of the LG into two contiguous segments with different *H*_Ind_ means (but the same dispersion) to find the split with the highest likelihood. The best split model was retained if the difference in likelihood compared to the no‐split model was significant (using a chi‐squared test with *χ* ^2^ = −2(LL_0_ − LL_1_) and *p* < 0.01). The split test was then applied to each resulting segment of the linkage group, followed by a third level of splitting. If, at any level, there was no significant split, the splitting function was stopped for that segment. Thus, the splitting function yielded between 1 and 8 segments with different *H*_Ind_ means. This process was repeated for each LG and individual.

A permutation test was run to look for clusters of splits for each linkage group and genetic group. Counts of significant splits were permuted over the possible boundaries between 1 cM windows to determine whether splits were clustered in certain parts of a linkage group. The observed variance in counts across a linkage group was calculated from 3 cM sliding windows of split counts, where each 3 cM window was the sum of the three 1 cM windows. Splits were then shuffled randomly among 1 cM windows by drawing counts from a multinomial distribution, ignoring any windows that had no markers on the linkage map. Permuted variance across a linkage group was calculated among 3 cM windows, following the approach for observed variance. Empirical *p*‐values were calculated for the observed variance compared to permuted variances from 10,000 replicates. Once all genetic groups were tested for each linkage group, the empirical *p*‐values were adjusted for multiple‐comparisons with a Benjamini–Hochberg correction (Benjamini & Hochberg, 1995).

Split clusters gave some indication of possible inversion boundaries; however, clusters could also represent changes in heterozygosity for other reasons, such as near centromeres or repeat regions. Visual inspection was used to infer if split clusters matched the patterns of inversions. *H*_Ind_ values were inspected using a plot of *H*_Ind_ for all individuals in a genetic group across each linkage group. Any bifurcations or trifurcations in *H*_Ind_ associated with split clusters indicated the presence of an inversion (Figure 1c).

Note that other types of chromosomal rearrangements and gaps in the linkage map can also cause heterozygosity differences among chromosomal regions and individuals (Mérot et al., 2020), which may falsely be called inversions using this type of analysis alone.

### 2.6 Inversion detection approach 2: window‐based PCA

A second approach used a 1 cM window‐based PCA to identify inversions by looking for clusters on the first principal component of SNP genotypes within each window. Inversions are expected to separate individuals into three distinct clusters, representing the separate karyotypes (Hanlon et al., 2022; Kemppainen et al., 2015; Nowling et al., 2020). The expected pattern is more complex for overlapping inversions on LG6 and LG14 of *L*. *saxatilis*, where three arrangements may be present, forming six clusters on a PC1 versus PC2 plot in a triangular pattern (Faria, Chaube, et al., 2019). Only PC1 was used in detection, but further analysis used PC1 and PC2 (see below). PCAs were conducted on all SNPs inside each 1 cM window using the *dudi*.*pca* command from the *adegenet* R package (Jombart, 2008; Jombart & Ahmed, 2011). Missing genotypes were imputed based on the mean score of the window with the *scaleGen()* function. PC1 scores for successive 1 cM windows were reorientated by switching the sign of any window where scores were negatively correlated with the preceding window. Adjusted PC1 scores were then visually inspected on the linkage map to detect regions with three clusters.

This approach is very similar to the detection approaches used in *local_PCA* (Li & Ralph, 2019) and *asaph* (Nowling et al., 2020). However, we developed a custom script so that local PCA could be run across the linkage map, as the reference genome is too fragmented to run existing tools.

### 2.7 Inversion boundaries

Inversion signals from heterozygosity splits and window‐based PCA were used to determine the approximate coordinates of inversion boundaries on the linkage map. However, these approaches were primarily meant to indicate the presence of polymorphic inversions, not specific breakpoint positions; thus, the edges of inversion signals can vary by a few cM among genetic groups. To attain a clearer resolution of inversion positions, the inferred boundaries were compared to the coordinates of published inversions (Hearn et al., 2022; Westram et al., 2021) on the new linkage map. If the published boundary positions corresponded to inversion patterns, the published positions were used to filter SNPs for genotyping the inversions with PCA; otherwise, we used the inferred boundaries of each genetic group for SNP filtering. Modified boundaries were used for the two missing inversions (see below) to avoid overlaps with neighbouring inversions; for LGC14.3 ([L]inkage [G]roup [C]luster 14.3; notation following Faria, Chaube, et al., 2019), SNPs between 12 and 34.66 cM were used, and for LGC12.3 SNPs between 46 and 50.09 cM were used. For new putative inversions inferred boundaries were used.

### 2.8 Inspecting and genotyping inversions

Putative inversions were inspected by genetic group through a PCA of all SNP genotypes within each inversion. These PCAs were run using the same command as the window‐based PCA. Additional PCAs were run for the full dataset (global PCA) and using only samples from locations where *L*. *arcana* was collected (*arcana‐saxatilis* PCA). Overlap between the coordinates of some inversions in LG12 and LG14 (other than the complex inversions) was observed, likely due to the low resolution of the genetic maps (Figures S12,S14, respectively). In such cases, the inversions were genotyped, excluding SNPs within those overlapping regions (see above).

As a control, the same analysis was run for the non‐inverted (colinear) regions of all linkage groups except for LG10 and LG12, which are mostly covered by inversions (Hearn et al., 2022; Westram et al., 2021). Colinear PCAs included all sites outside of an inversion, merging any potential left and right segments of the respective linkage group (e.g. LG9) and excluding a 2 cM buffer around inversions to account for the imprecise positions of boundaries (see Faria, Chaube, et al., 2019; Westram et al., 2021).

The presence of an inversion was supported by inspecting a scatterplot of PC1 versus PC2 for three clusters of points, which should be divided on PC1 (Figure 1e). For the complex inversions on LG6 and LG14, six clusters were expected to form *a triforce* shape (i.e. triangle within a triangle, from the Zelda video game series), with each vertex‐cluster representing one of three possible homokaryotypes and the heterokaryotype clusters found on each vertex of the inner triangle (Figure 1e).

Clusters were assigned with a K‐means clustering algorithm in R. One hundred random starting positions were tested for each inversion. The starting positions that had the highest variation between groups, measured as the sum of squares between groups, were retained. Two clustering counts (2 ≤ K ≤ 3) were tested for most inversions; additional clusters were considered (2 ≤ K ≤ 9) for complex inversions. Different K values were compared using the silhouette method in the R package *cluster*, retaining the K with the highest mean silhouette value. For simple inversions, the expected K = 2 or 3 fits were found using PC1 for clustering. For complex inversions, both PC1 and PC2 were considered in the K‐means clustering. In the case of LGC14.1/2, 6 clusters were found only after separating the inversion into two parts (i.e. the simple section of the inversions, LGC14.1 with three clusters; and the complex section where the two inversions overlap, LGC14.2 with six clusters).

Cluster scores were converted into genotypes by adjusting the labels for a consistent order among inversions, whereby the homokaryotypic cluster containing the most *crab* samples was labelled ‘RR’, the reference arrangement, while the other homokaryotypic cluster was labelled alternatively ‘AA’. LGC6.1/2 and LGC14.2 had three arrangements. For these cases, the homokaryoptypic cluster furthest from ‘RR’ on PC1 was labelled ‘A_1_A_1_’ and the final homokaryotypic cluster was labelled ‘A_2_A_2_’. Some inversion labels were manually adjusted to correct for noise from geographic or species diversity (see Supporting Information). Inversion PCAs with only two clusters could represent ‘RR’ and ‘AA’ or ‘RR’ and ‘RA’. In such cases, the global PCA, heterozygosity split plots and average heterozygosity scores were used to determine the presence or absence of the heterokaryotypic cluster. The frequency of each inversion arrangement was calculated from the counts assigned to clusters. An inversion was considered polymorphic at a sampling site if individuals from that site were heterokaryotypic or if more than one arrangement homokaryotype was present.

### 2.9 Inferring ancestral arrangement using *L*. *compressa*

The ancestral arrangements for each inversion were inferred by projecting genotypes from four *L*. *compressa* onto the PCA plot using the R function *suprow* from the *adegenet* package. Projection improved the resolution of inversion genotypes by preventing the PCA from being dominated by interspecific differences. The arrangement shared with *L*. *compressa* was considered ancestral. However, in a few cases, inversions were also polymorphic in *L*. *compressa* (Table 2). Deeper sampling of *L*. *compressa* may reveal additional polymorphic inversions, and, therefore, our inference of ancestry should be seen as preliminary.

### 2.10 Association of inversion frequencies with ecotypes

To identify the inversions contributing to divergence, we compared arrangement frequencies among ecotypes while controlling for variation among sampling locations. Three logistic regression models were run for each inversion:

*Null*: *N*_inv_ ~ location
*Eco*: *N*_inv_ ~ ecotype + location
*Int*: *N*_inv_ ~ ecotype × location

Inversion frequencies (*N*_inv_) were considered as a count of two possible states, either the ‘R’ or ‘A’ arrangement. For the complex inversions, LGC6.1/2 and LGC14.2, the ‘A_1_’ and ‘A_2_’ arrangements were summed to get the ‘A’ state count. Subsequent models were run for these inversions to test for differences between ‘A_1_’ and ‘A_2_’. Models were evaluated through a hierarchical comparison of AIC. First, the null model was compared to the ecotype model to establish if there was an ecotype effect (i.e. AIC_Null_ − AIC_Eco_). If *∆*AIC > 2, then *Int* and *Eco* were compared to establish the significance of the ecotype‐location interaction. If AIC_Null_ < AIC_Eco_, then *Null* and *Int* were compared instead. Only when AIC_Int_ − AIC_Eco_ > 2 was *Eco* the best model. The best model was further evaluated to determine the proportion of variance explained using Cohen's pseudo‐*R* ^2^ (1 − null deviance/fitted model deviance) and a *p*‐value estimated from the deviance associated with each term (Tables S4–S6). *p*‐values were adjusted using a Benjamini‐Hochberg correction.

Logistic regressions were run for two ecotypes and two species contrasts per inversion. Before running the logistic regression, the data were filtered to keep samples only from locations where both focal ecotypes (or species) were collected, as ecotypes have different distributions (with the *crab* and *wave* being the most widely distributed; Reid, 1996, pp. 305–318). The ecotype contrasts were between *crab* and *wave* or *wave* and *barnacle*. Other contrasts were not possible due to insufficient location overlaps. For the species contrasts, we compared the arrangement frequencies in *L*. *arcana* separately with the *wave* and *crab* ecotypes of *L*. *saxatilis* for sites where both species were sampled. As *crab* and *L*. *arcana* were only co‐sampled in a single location, the interaction model was not fitted, and the null model was simplified to *N*_inv_ ~ 1. Arrangement counts were expected to be more similar between *L*. *arcana* and the *wave* ecotype because *L*. *arcana* was found mainly in wave‐exposed habitats at the sampled locations.

## 3 RESULTS

### 3.1 Detecting inversions

**FIGURE 2:**
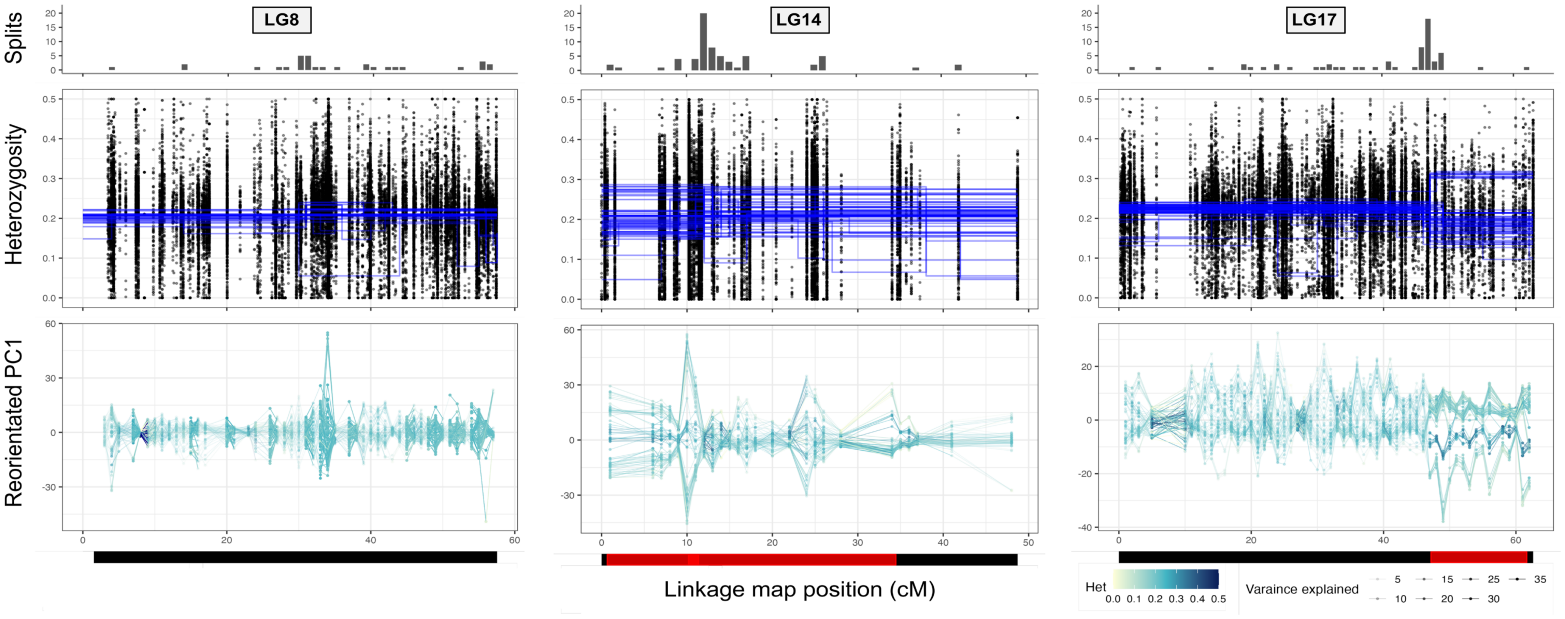
Three examples of the inversion detection results using Northern saxatilis data. From left to right, there is an example of a linkage group without an inversion (LG8), an example of an opaque pattern of multiple inversions (LG14) and a clear example of a single inversion (LG17). *Top panel*: shows the number of significant splits of heterozygosity between 1 cM windows of the linkage map. *2nd panel*: shows the results of the heterozygosity splits approach, where each dot represents the proportion of heterozygous SNPs per contig for each snail, and the blue lines represent the average heterozygosity between two significant splits for an individual (*H*_ind_). The *y*‐axis was limited to 0.5, and only contigs on the linkage map are shown. *3rd panel*: PC1 scores from a PCA for each 1 cM window. These scores were reorientated to positively correlate with the preceding window's scores. Lines and dots are coloured by average heterozygosity across the window and shaded by the percentage variation on PC1. Heterozygosity exceeding 0.5 was coloured dark blue. *Bottom panel*: red bars show the position of published inversions (Westram et al., 2021) on each linkage group. Overlapping boundaries between LGC14.1/2 (left) and LGC14.3 (right) are in lighter red. Plots for the other groups and inversions are in the Supporting Information (Figures S1–S17).

**FIGURE 3:**
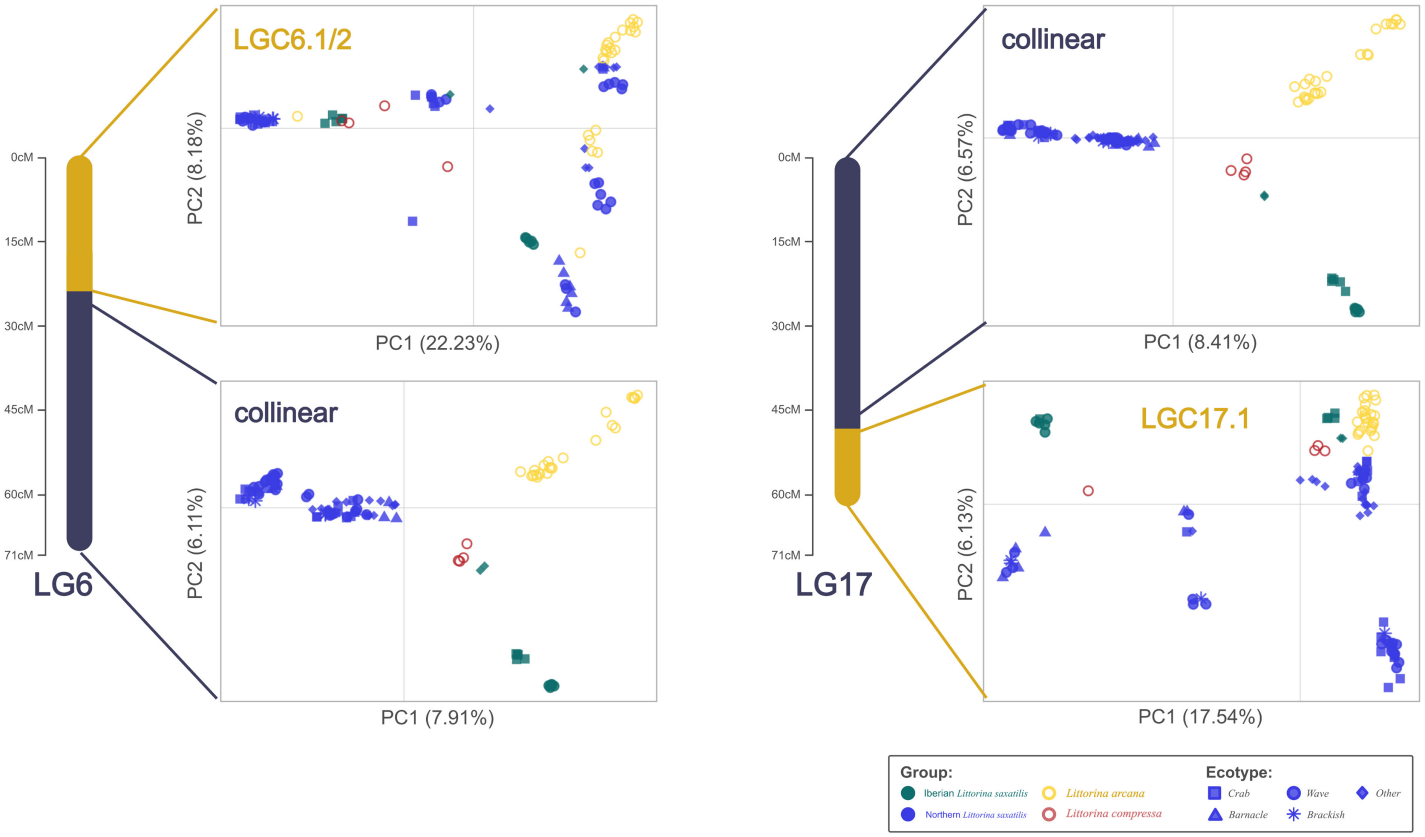
PCA plots for the colinear and inverted segments of two linkage groups using the combined data from all snails. Points are coloured by genetic group and shaped by ecotype. PC1 is on the *x*‐axis and PC2 on the *y*‐axis. Gridlines mark the origin. The bars left of the plots represent linkage groups, with the inverted regions coloured yellow. Plots for other linkage groups are in the Supporting Information (Figure S18).

The heterozygosity split approach and window‐based PCA provided evidence of polymorphic chromosomal inversions on most linkage groups (Table 1; Figure 2 and Figures S1–S17), most of which were identified in previous studies (Faria, Chaube, et al., 2019; Hearn et al., 2022; Westram et al., 2021). Heterozygosity splits clustered significantly on all linkage groups, with the ratio of observed versus permuted variance being higher on those containing published inversions (Table S2). In most cases, these clusters matched published positions of inversion boundaries (Hearn et al., 2022; Westram et al., 2021), with patterns of *H*_Ind_ and window‐based PCA consistent with inversions (Table 1). Typically, the detection methods were supported by PCA of the whole inverted region, which showed either 3 or 6 clusters, with some variation within clusters driven by geography and clearly different from PCAs for the collinear regions (Figure 3 and Figure S18).

**TABLE 1:** Summary of inversion detection for all genetic groups and linkage groups.

LG	Inversion	Group	Hetero. splits	PCA per cM	Inv. pos. (cM), Het|PCA	K	Fig. Ref.
LG1	LGC1.1	Northern saxatilis	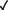	?	0–14	2	S1
		Iberian saxatilis	?	?	0–16|0–15	3	
		*Littorina arcana*	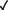	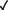	0–17|0–15	3	
					**0–19**		
	LGC1.2	Northern saxatilis	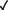	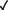	77–95.6*|76–95.6*	3	S1
		Iberian saxatilis	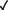	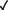	76–95.6*	3	
		*Littorina arcana*	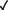	?	77–95.6*	3	
					**76.1–95.6**		
LG2	LGC2.1	Northern saxatilis	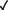	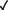	0–14|0–11*	3	S2
		Iberian saxatilis	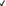	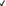	0–12	2	
		*Littorina arcana*	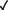	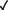	0–12	3	
					**0.3–10.9**		
LG3	No inversion						S3
LG4	LGC4.1	Northern saxatilis	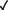	?	0–21|7–22	3	S4
		Iberian saxatilis	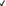	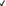	0–23	2	
		*Littorina arcana*	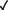	✕	0–24|–	2	
					**5.1–22.8**		
LG5	LGC5.1^!^	Northern saxatilis	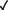	?	15–46|–	3	S5
		Iberian saxatilis	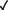	?	15–47|16–47	2	
		*Littorina arcana*	?	?	16–47|–	3	
LG6	LGC6.1/2	Northern saxatilis	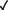	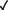	0–19	6	S6
		Iberian saxatilis	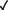	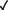	0–19	3	
		*Littorina arcana*	?	?	0–19|0–18	4	
					**0–24.3**		
LG7	LGC7.1	Northern saxatilis	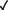	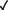	37–43*	3	S7
		Iberian saxatilis	?	?	37–43*	2	
		*Littorina arcana*	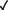	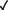	37–42*	3	
					**36.6–42.6**		
	LGC7.2	Northern saxatilis	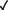	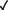	45–59.5|46–55	3	S7
		Iberian saxatilis	?	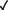	45–59.5|46–55	3	
		*Littorina arcana*	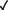	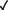	44–59.5|47–55	3	
					**46.9–58.2**		
LG8	No inversion						S8
LG9	LGC9.1	Northern saxatilis	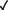	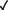	18–42*	3	S9
		Iberian saxatilis	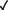	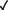	18–42*|18–41*	3	
		*Littorina arcana*	?	✕	18–42*|–	2	
					**18.7–42.1**		
	LGC9.2^!^	Northern saxatilis	?	✕	52–59.5|–	3	S9
		Iberian saxatilis	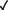	?	52–59.5|52–55	3	
		*Littorina arcana*	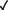	?	53–59.5|52–55	3	
LG10	LGC10.1	Northern saxatilis	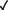	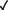	0–3*	3	S10
		Iberian saxatilis	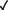	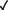	0–3*	3	
		*Littorina arcana*	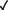	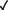	0–3*	3	
					**0.9–2.8**		
	LGC10.2	Northern saxatilis	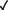	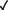	4–45.5*	3	S10
		Iberian saxatilis	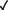	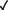	14–45.5	2	
		*Littorina arcana*	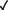	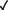	14–45.5	3	
					**3.1–44.1**		
LG11	LGC11.1	Northern saxatilis	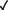	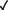	56–70|58–69	3	S11
		Iberian saxatilis	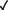	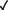	56–70|58–69	2	
		*Littorina arcana*	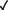	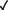	55–70|58–69	3	
					**50.9–69.2**		
LG12	LGC12.1	Northern saxatilis	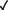	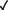	0–38|16–34	3	S12
		Iberian saxatilis	✕	✕	–	3	
		*Littorina arcana*	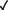	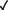	0–39|16–38	3	
					**9.3–40.5**		
	LGC12.2	Northern saxatilis	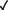	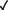	39–46|35–46	3	S12
		Iberian saxatilis	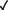	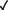	39–49|39–46	3	
		*Littorina arcana*	✕	✕	–	3	
					**39.3–45.6**		
	LGC12.3	Northern saxatilis	?	?	39–46	2	S12
		Iberian saxatilis	?	?	39–46	3	
		*Littorina arcana*	✕	✕	–	2	
					**40.5–50.1**		
	LGC12.4	Northern saxatilis	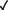	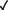	52–69.9|51–69.9	3	S12
		Iberian saxatilis	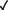	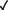	52–69.9|51–69.9	3	
		*Littorina arcana*	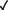	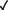	53–69.9|52–69.9	3	
					**48.3–68.2**		
LG13	No inversion						S13
LG14	LGC14.1/2	Northern saxatilis	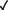	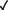	0–12*	7	S14
		Iberian saxatilis	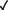	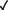	0–12*	5	
		*Littorina arcana*	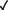	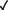	0–12*	9	
					**0.7–11.4**		
	LGC14.3	Northern saxatilis	✕	✕	–	2	S14
		Iberian saxatilis	✕	✕	–	2	
		*Littorina arcana*	✕	✕	–	2	
					**10.2–34.7**		
LG15	No inversion						S15
LG16	No inversion						S16
LG17	LGC17.1	Northern saxatilis	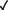	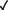	47–62.6*	3	S17
		Iberian saxatilis	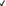	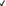	47–62.6*	2	
		*Littorina arcana*	✕	✕	–	3	
					**47.5–62.0**		

*Note*: New putative inversions are marked with ‘!’. ‘
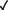
’ represents a clear pattern of a polymorphic inversion. Smaller ‘
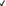
’ are cases where the heterokaryotype was not detected, ‘?’ represent uncertain patterns and ‘✕’ represent no pattern. Inversion positions were inferred from both identification approaches, each separated by a ‘|’. When both approaches aligned, only a single set of boundaries is presented. Missing inversions are represented by ‘–’. ‘*’ indicates matches with the published inversion boundaries (Westram et al., 2021; and Hearn et al., 2022 for LGC12.2 and LGC12.3), converted onto the new linkage map (in bold). PCAs were run per genetic group for all SNPs within these positions to validate the presence of inversions: ‘K’ is the optimal number of clusters found via K‐means using PC1, or PC1 and PC2 for complex inversions. ‘Fig. ref’ indexes the corresponding plots in the Supporting Information.

A clear example is LGC17.1 (Figure 2): this inversion was detected in all *L*. *saxatilis* populations, with consistent boundaries corresponding to those previously reported from Sweden (Westram et al., 2018, 2021), but it was not polymorphic in our *L*. *arcana* sample. However, not all published inversions were as easily identified, or their boundaries did not clearly match expectations, especially on LG14 (Figure 2) and LG10 (Figure S10). Heterozygosity varied widely among individuals across the length of both linkage groups, making inversion patterns hard to distinguish. Iberian snails had three inversions (LGC2.1, LGC4.1 and LGC17.1) where only putative homokaryotypes for each arrangement were found (Figures S2, S4 and S17), possibly reflecting strong genetic differentiation between ecotypes (Kess & Boulding, 2019). Finally, patterns of inversions at the expected genomic locations of LGC12.3 and LGC14.3 were absent across the entire data set.

### 3.2 Distribution of inversions within and between species

Most published inversions (89%) were identified as widespread polymorphisms across the species range of *L*. *saxatilis*. Indeed, all inversions were polymorphic in Northern saxatilis, excluding the two aforementioned exceptions of LGC12.3 and LGC14.3 (Table 2; Figure S18). Four additional inversions were not clearly identified in the PCA of Iberian saxatilis (LGC1.1, LGC7.1, LGC12.1 and LGC14.2), although LGC1.1 and LGC7.1 were identifiable from the global PCA of all snails (Figure S18). LGC12.1 may also be present in the global PCA; however, its polymorphic status rests on a single snail from a genetically distinct Iberian location (Burela). Lastly, no heterokaryotype clusters were identified in Iberia for three inversions (LGC2.1, LGC4.1 and LGC17.1) from either the Iberian (Figure S19) or global PCA (Figure 3 and Figure S18).

**TABLE 2:** Polymorphic status and ancestry of inversions.

Location	*N*	LGC1.1	LGC1.2	LGC2.1	LGC4.1	LGC6.1/2	LGC7.1	LGC7.2	LGC9.1	LGC10.1	LGC10.2	LGC11.1	LGC12.1	LGC12.2	LGC12.3	LGC12.4	LGC14.1	LGC14.2	LGC14.3	LGC17.1	LGC5.1	LGC9.2
Northern *Littorina saxatilis*																						
Arsklåvet	4	N	Y	Y	Y	Y	Y	Y	Y	Y	Y	Y	Y	Y	–	Y	Y	Y	–	Y	N	–
Broad Haven	1	N	N	N	Y	Y	Y	N	Y	Y	N	N	N	Y	–	N	Y	Y	–	N	N	–
Ceann Tra	2	N	N	Y	N	N	Y	Y	N	Y	N	N	N	N	–	N	N	N	–	N	Y	–
Dersingham	2	N	N	N	N	N	N	N	N	N	N	N	N	N	–	N	N	N	–	N	N	–
Holyhead	12	Y	Y	Y	Y	Y	Y	Y	Y	Y	Y	Y	Y	Y	–	Y	Y	Y	–	Y	Y	–
Laugarnes	2	N	Y	Y	Y	N	N	Y	Y	Y	Y	Y	Y	Y	–	Y	Y	Y	–	N	N	–
Oban	1	N	N	N	Y	N	Y	N	N	N	N	N	N	N	–	Y	Y	Y	–	Y	N	–
Port Saint Mary	1	N	N	N	N	N	Y	N	N	N	N	N	N	Y	–	Y	N	N	–	N	N	–
Ramsö	4	Y	Y	Y	Y	Y	Y	Y	Y	Y	Y	Y	Y	Y	–	Y	Y	Y	–	Y	N	–
Ravenscar	7	N	Y	Y	Y	Y	N	Y	Y	Y	Y	Y	Y	Y	–	Y	Y	Y	–	Y	N	–
Roscoff	6	Y	Y	Y	Y	Y	Y	Y	Y	Y	Y	Y	Y	Y	–	Y	Y	Y	–	Y	N	–
Saint Abbs	2	Y	N	Y	N	Y	Y	N	N	Y	N	Y	N	N	–	Y	N	N	–	Y	Y	–
Thornwick	8	Y	Y	N	Y	Y	Y	Y	Y	Y	Y	Y	Y	Y	–	Y	Y	Y	–	N	Y	–
Tjärnö	2	N	N	N	N	N	N	N	N	N	N	N	Y	Y	–	N	N	N	–	Y	N	–
Trondheim Fjord	3	N	N	Y	N	N	Y	Y	Y	N	N	Y	N	Y	–	Y	Y	Y	–	N	Y	–
Varanger Fjord	4	N	Y	Y	Y	Y	N	Y	Y	N	N	N	Y	Y	–	N	N	N	–	N	Y	–
White Sea	2	N	N	N	N	N	N	N	N	N	N	N	N	Y	–	N	Y	Y	–	Y	N	–
York ME	4	N	Y	Y	N	Y	N	Y	Y	N	Y	N	N	Y	–	Y	Y	Y	–	N	N	–
*Littorina arcana*																						
Amble	1	N	N	Y	–	–	Y	N	–	N	Y	N	N	–	–	N	–	Y	–	–	Y	N
Broad Haven	1	Y	Y	N	–	–	N	N	–	N	N	N	N	–	–	N	–	Y	–	–	N	N
Holyhead	4	Y	Y	Y	–	–	N	N	–	Y	Y	Y	Y	–	–	Y	–	N	–	–	N	Y
Ravenscar	7	N	Y	Y	–	–	Y	Y	–	Y	Y	Y	Y	–	–	Y	–	Y	–	–	N	N
Roscoff	4	N	N	N	–	–	N	Y	–	Y	N	Y	Y	–	–	Y	–	Y	–	–	N	N
Saint Abbs	2	Y	N	N	–	–	Y	Y	–	N	N	Y	Y	–	–	Y	–	Y	–	–	Y	N
Trondheim Fjord	1	N	Y	N	–	–	N	N	–	N	N	Y	N	–	–	N	–	Y	–	–	N	N
Varanger Fjord	4	Y	Y	N	–	–	Y	Y	–	Y	Y	Y	N	–	–	Y	–	Y	–	–	N	N
Iberian *Littorina saxatilis*																						
Burela	2	–	N	N	N	Y	–	Y	N	N	Y	Y	–	N	–	N	N	–	–	N	N	N
Centinela	10	–	Y	Y	Y	Y	–	Y	Y	Y	Y	Y	–	Y	–	Y	Y	–	–	Y	Y	Y
Ancestry		R	R	A	P	R	A	A	R	A	A	R	R	P	P	A	P	A_1_	–	R	–	R

*Note*: The PCA of each genetic group was used to determine the polymorphic status at each location. If samples from a single location were present in two or more karyotypes, or just the heterokaryotype, the inversion was considered polymorphic. The heterokaryotype cluster was determined by inspecting the global PCA plot. Polymorphic inversions are marked with a ‘Y’ on a green background. Non‐polymorphic inversions are marked with an ‘N’ on a red background. Ancestry was determined by the position of *L*. *compressa* with either the ‘A’, 'A_1_' or ‘R’ arrangement cluster in the global PCA. Any ‘P’ represents a case where *L*. *compressa* overlapped with the heterokaryotype or occurred in two clusters, suggesting polymorphism. Any plots without clear clusters are represented by ‘–’. N represents the sample size. The two novel inversions from this study are listed separately on the far right. Site coordinates are in Table S1.

For most inversions, the PCAs of inversion areas clustered snails by karyotype on PC1, combined with a signal of geographic structure on PC2 (Figure 3 and Figure S18,S19). The strongest geographic signal was the separation of the Iberian from the Northern saxatilis, which typically was stronger in one of the two arrangements. Iberian saxatilis also formed a distinct cluster in the colinear genome (Figure S18). A finer scale geographic separation also existed within the Northern saxatilis (Figures S19,S20). North American samples were often outliers on PC2, and snails sampled from the North Sea (excluding Dersingham, UK) clustered together within and outside inversions. All inversion polymorphisms were identified in the most densely sampled locations in Sweden (Ramsö), France (Roscoff), Wales (Holyhead) and Spain (Centinela), with a diffuse presence at other sites (Table 2). Of note, two‐thirds of inversions were polymorphic in the single North American site (York, ME, USA), and no polymorphisms were identifiable in a *brackish* ecotype site (Dersingham, UK). However, the lack of observed polymorphism at a site does not mean inversions are absent or fixed, as many sites had too few samples (≤2) to detect rare arrangements.

Most of the previously identified inversion polymorphisms observed in *L*. *saxatilis* were also polymorphic in *L*. *arcana* (Table 2). On the PCA plots of inversion regions, *L*. *arcana* typically clustered separately from *L*. *saxatilis* within each karyotype (Figure 3 and Figure S18). For the colinear genome, *L*. *arcana*, Northern saxatilis and Iberian saxatilis formed three groups with similar separation on PCA axes (Figure S18). Within inversions, PC2 explained most of the difference between species, with the variation on PC1 in *L*. *arcana* being consistent with the inversion karyotypes in *L*. *saxatilis*, suggesting the presence of the same arrangements (Figure S21). There was also finer‐scale geographic separation within *L*. *arcana*, consistent with the North Sea clustering seen in *L*. *saxatilis* (Figure S19).

Only two inversion polymorphisms (LGC12.2 and LGC17.1) found in *L*. *saxatilis* were not identified in *L*. *arcana* (Table 1). Both inversions were polymorphic in the outgroup, *L*. *compressa*, suggesting that one arrangement was either lost or fixed in *L*. *arcana* (Figure S18). The status of LGC4.1 and LGC14.1/2 in *L*. *arcana* was uncertain. PCA showed diffuse clusters of samples for the same position on the linkage map, but they were poorly aligned with the *L*. *saxatilis* clusters (Figure 2 and Figures S18,S21). Further investigation identified weak patterns in *L*. *arcana* consistent with polymorphism for LGC4.1, but only the first part of LGC14.1/2 (LGC14.1, which appears to be absent in *L*. *arcana*) could be aligned (Figure S22).

### 3.3 Identification and distribution of new inversion polymorphisms

In addition to identifying previously described inversions from Sweden (Hearn et al., 2022; Westram et al., 2021), new patterns were found on LG5 and LG9 that are consistent with polymorphic inversions (Figures S5,S9). Following established convention (Faria, Chaube, et al., 2019), these were labelled LGC5.1 and LGC9.2 (Table 1). However, these patterns were not found in all genetic groups, and their patterns were inconsistent between the heterozygosity split and PCA‐based approaches. PCA for LGC5.1 was similar to the collinear region of LG5, casting doubt on the inversion status of this genomic region (Figure S18). While PCA for LGC9.2 showed a signal that looked like an inversion polymorphism at low frequency, upon further inspection, this was seen to be geographic variation (see Supplementary Method; Figure S22). The patterns for LGC9.2 for other genetic groups and the global PCA were clear (Table 2; Figure S18). Both putative inversions appear fixed for the more common arrangement in Sweden (Table 2), explaining why they could have been missed in previous studies (Faria, Chaube, et al., 2019; Westram et al., 2021).

### 3.4 Associating inversion frequencies with ecotypes and species

**FIGURE 4:**
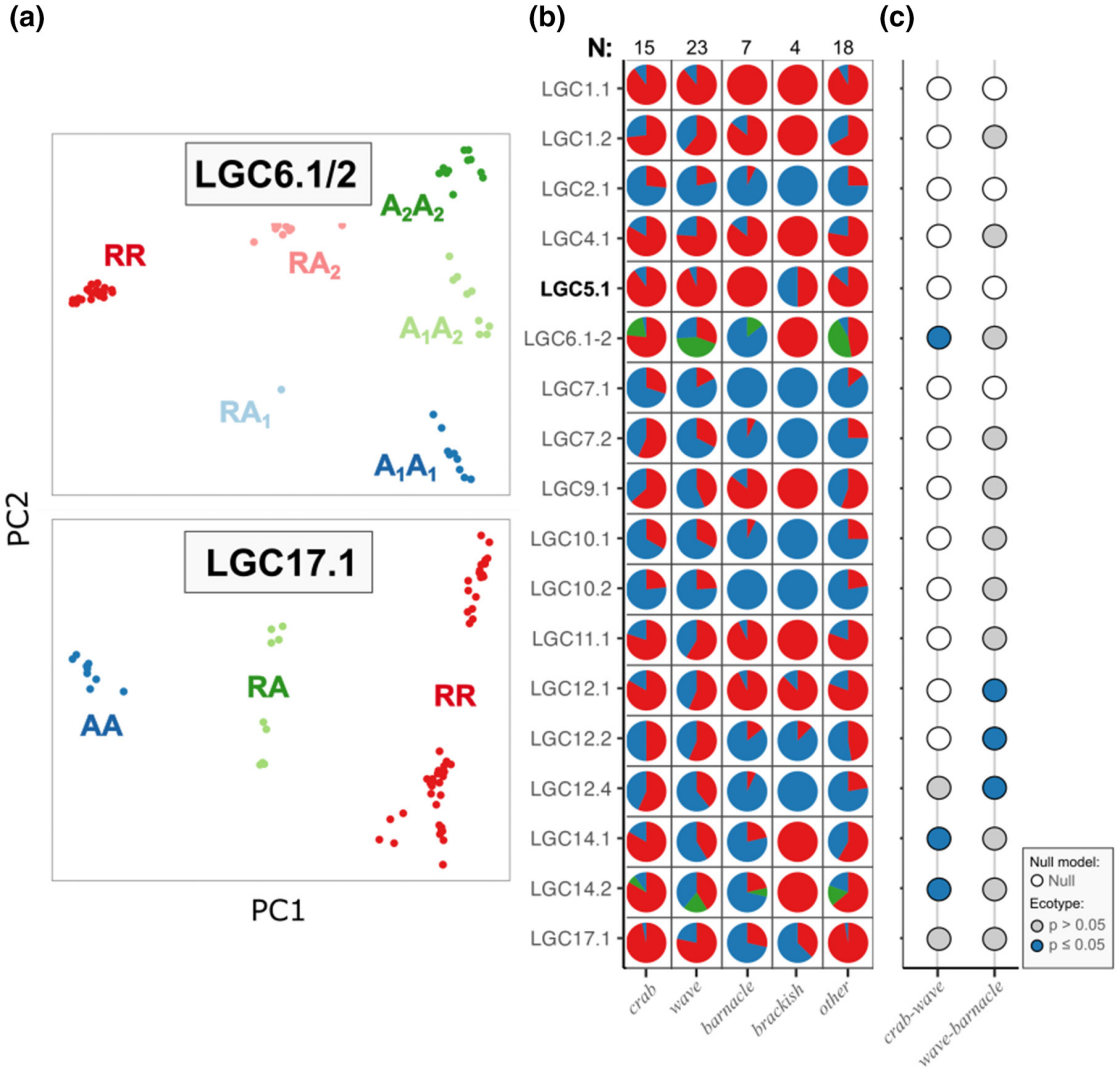
Inversion arrangement frequencies in Northern *Littorina saxatilis*. (a) Two examples of PCA plots, coloured by genotype. LGC6.1/2 with six genotypes (complex inversion) and LGC17.1 with three genotypes. ‘R’ = reference, ‘A’ = alternative, with ‘A’ subdivided into ‘A_1_’ = alternative 1 and ‘A_2_’ = alternative 2, the latter corresponding to the third arrangement in LGC6.1/2 (Faria, Chaube, et al., 2019). Plots for other inversions are in Supporting Information (Figure S19). (b) Arrangement frequencies for all inversions (new inversion in bold font) with either two or three arrangements. Colours represent the arrangements (R = red; A or A_1_ = blue; A_2_ = green). Separate pie charts were made for each ecotype. Sample sizes for each ecotype are shown above the panel. (c) Logistic regression results grouped by ecotype contrast. The fill of circles represents the best model evaluated by AIC: empty = null model and filled = ecotype model. Blue circles were significant for the ecotype effect (*p* ≤ 0.05). For these tests, A_1_ and A_2_ were grouped together. Ecotype contrasts subset individuals to locations where both focal ecotypes were sampled. Detailed results are in Tables S4,S6.

Northern saxatilis ecotype contrasts found that different inversions contribute to divergence between the *crab*–*wave* and *wave*–*barnacle* ecotypes. Considering all sites together, 11 of 19 inversions showed clear arrangement frequency differences between the *crab–wave* ecotypes and 15 of 19 inversions for the *wave* and *barnacle* ecotypes (Figure 4b). However, when a logistic regression analysis was used to account for frequency differences driven by location, only LGC6.1/2, LGC14.1 and LGC14.2 had significant ecotype‐related differences for the *crab–wave* contrasts (Figure 4c; Tables S3, S4 and S6). LGC14.1 and LGC14.2 frequencies were better explained by a model containing an interaction term between ecotype and location (Tables S4,S6), indicating that the role of these inversions may vary with location. For example, the arrangement frequency differences in LGC14.1 were strong at only two of five sampling locations (Table S3). Meanwhile the three tested inversions on LG12 were significant for *wave–barnacle* contrasts (Figure 4c; Tables S3, S4 and S6), with marginal signals in several other inversions that were lost after correcting for multiple tests.

**FIGURE 5:**
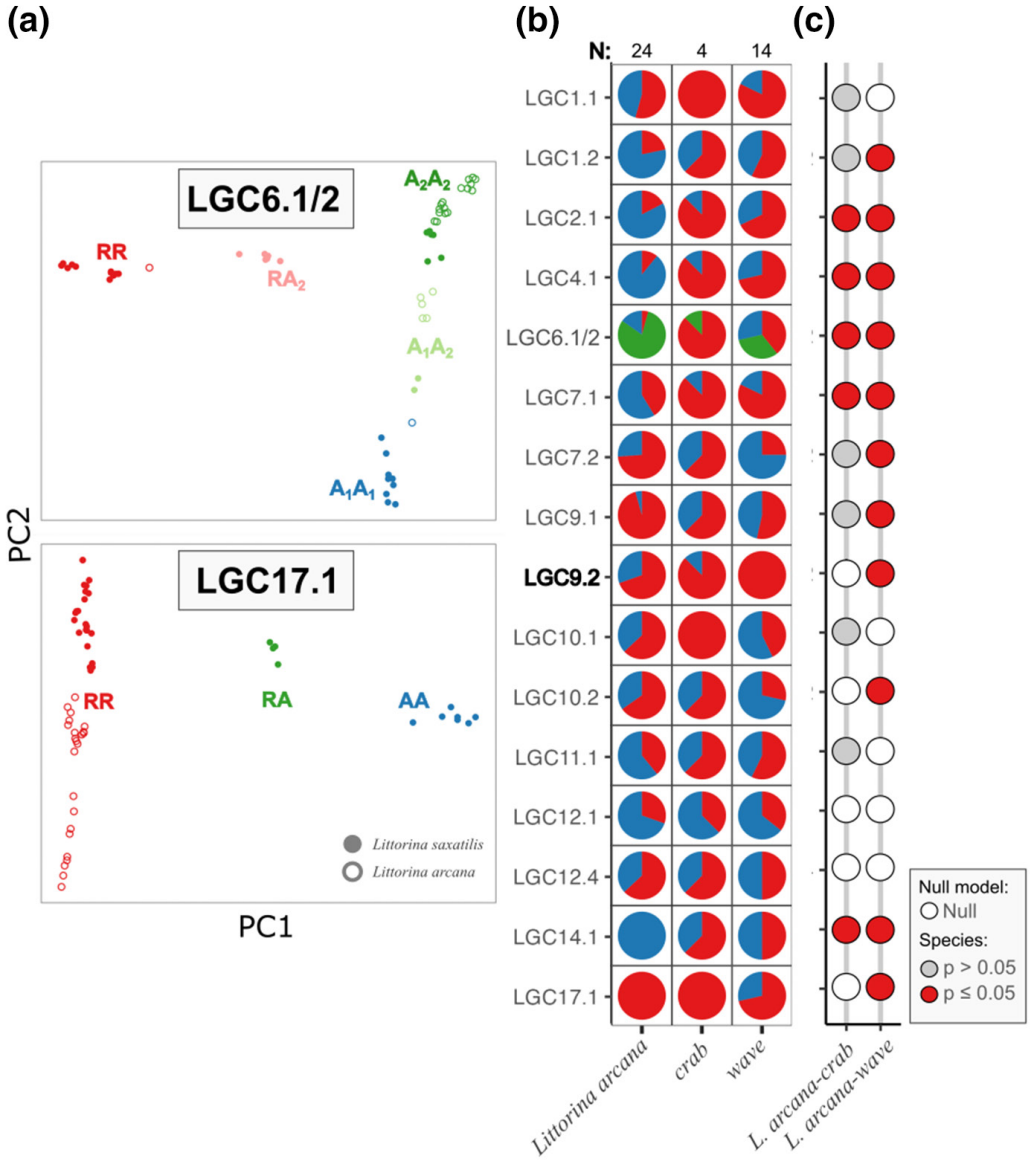
Inversion arrangement frequency comparisons between *Littorina saxatilis* and *L*. *arcana*. Only locations where both species were collected were included. (a) Example PCA plots for two inversions coloured by genotype. ‘R’ = reference, ‘A’ = alternative, with ‘A’ subdivided into ‘A_1_’ = alternative 1 and ‘A_2_’ = alternative 2, the latter corresponding to the third arrangement in LGC6.1/2 (Faria, Chaube, et al., 2019). Plots for other inversions are in Supporting Information (Figure S20). (b) Arrangement frequencies for all inversions (new inversion in bold font) with either two or three arrangements. Colours represent the arrangements (R = red; A or A_1_ = blue; A_2_ = green). Separate pie charts have been made for *L*. *arcana* and two *L*. *saxatilis* ecotypes. Sample sizes for each group are shown above the panel. (c) Logistic regression results grouped by species contrast. The fill of circles represents the best model evaluated by AIC: empty = null model and filled = species model. Red circles were significant for the species effect (*p* ≤ 0.05). For these tests, A_1_ and A_2_ were grouped together. Since *L*. *arcana* and *crab* ecotypes were only found together in one location, the interaction model left out these contrasts. Detailed results are in Tables S5,S6.

The species contrasts between *L*. *saxatilis* ecotypes and *L*. *arcana* had more signatures of divergence than the *L*. *saxatilis* ecotype contrasts (Figure 5). Apart from LGC12.1, arrangement frequencies were different between species for all inversions. Breaking this down to *L*. *saxatilis* ecotypes, 15 of the 16 tested inversions were different between *L*. *arcana* and *wave*, while 13 of the 16 inversions were different between *L*. *arcana* and *crab* (Figure 5b). Logistic regression found five inversions with significant differences in arrangement frequencies among *L*. *arcana* and the *crab* ecotype, and 11 significant differences among *L*. *arcana* and the *wave* ecotype (Figure 5c; Tables S5,S6). Three of the significant inversions (LGC1.2, LGC7.1 and LGC10.2) in the *L*. *arcana‐wave* contrasts were better explained by a model considering the interaction between species and location (Table S5), suggesting that some interspecific differences may be location‐specific.

## 4 DISCUSSION

We have shown that several inversions identified in Swedish populations of *L*. *saxatilis* are distributed across the species range (Figure 2; Tables 1, 2). The arrangement frequencies for some of these inversions were different among ecotypes (Figure 4). Since inversions contribute to divergent adaptive phenotypes in Sweden, this suggests that they have a widespread role in parallel ecotype formation (Koch et al., 2021, 2022; Morales et al., 2019; Westram et al., 2021). These inversions are possibly ancient polymorphisms, as several were found to be polymorphic in the sister species *L*. *arcana* (Figure 5).

By aggregating samples over broad areas, our inversion detection approaches clearly showed that all previously published inversion polymorphisms detected in Sweden for *L*. *saxatilis* (Hearn et al., 2022; Westram et al., 2018, 2021) are widespread across the species' native range, with two exceptions (Figure 2 and Figure S1–S17; Table 1). One exception was LGC14.3 (Figure 2 and Figure S14; Table 1), which was only weakly supported in the original description (Faria, Chaube, et al., 2019). The other was LGC12.3, which was only detected when males and females were analysed separately in the original description (Hearn et al., 2022). The widespread distribution of most inversions across the species range is surprising considering the strong geographic structure within *L*. *saxatilis*, especially the separation between Iberian and North Atlantic populations that is believed to result from long‐term isolation on either side of the Bay of Biscay (Doellman et al., 2011; Morales et al., 2019; Panova et al., 2014; Tirado et al., 2016). Moreover, geographic differences in the PCA plots were smaller than the differences among inversion karyotypes (Figure 3 and Figure S18), consistent with an ancient origin of the inversion polymorphisms and low gene flux between arrangements. Interestingly, geographic structure was typically stronger in one of the arrangements (e.g. LGC17.1; Figure 3) which might be explained by the recent geographic spread of one arrangement (or a derived haplotype within one arrangement) across the species range, perhaps driven by selection.

We also identified patterns consistent with two additional inversion polymorphisms (LGC5.1 and LGC9.2). However, our detection approaches did not always align. One reason is that the success of the heterozygosity split approach is impacted by the age and frequency of an inversion. Young inversions may be missed if the inverted homokaryotype is rare in a population, since the rise in heterozygosity in heterokaryotypes will be hard to perceive until the inverted arrangement accumulates substitutions and becomes common in a population. Old inversions may be missed when the heterokaryotype is rare, since genetic diversity accumulates over time, restoring heterozygosity in the inverted homokaryotype. On the other hand, uncommon inversions may be easier to detect using the heterozygosity split approach than with PCA because a few individuals can stand out on the split plot, whereas they would not contribute enough variance to group individuals on PC1. Analyses with simulations and inversions of known age are needed to evaluate the heterozygosity split approach more fully relative to other approaches. Identification of inversions was harder in Iberia due to a small sample size (*n* = 12) and a phylogeographic barrier between Iberian sampling sites (Tirado et al., 2016). In addition, inversion frequency differences are stronger between ecotypes in Iberia (Morales et al., 2019), and hybrids are less common (Kess & Boulding, 2019), resulting in fewer heterokaryotypes and making our detection methods less effective. Because our approaches can only infer patterns of variation that are typical of inversions, breakpoint sequencing using long‐read sequencing in combination with LD analyses, could be used to validate our observations.

Genotyping of the inversions showed differences in arrangement frequency that could be explained if some of the loci contributing to ecotype formation were present within inversions. Around 35% of the tested inversions (7 of 19) had significant differences in arrangement frequency among *crab–wave* or *wave–barnacle* ecotypes in Northern saxatilis (Figure 4; Tables S4,S6), indicating that several inversions relate to ecotype formation. The general pattern was that differences between the former ecotype contrast were only associated with a limited set of inversions, LGC6.1/2 and LGC14.1/2, which matched some of the previously published *crab–wave* candidate regions (Koch et al., 2022; Westram et al., 2018, 2021), while *wave–barnacle* differences were located on LG12. These *wave–barnacle* differences may relate to shore height, as LG12 has previously been associated with shore height gradients (Morales et al., 2019). Alternatively, this might relate to sex, since LGC12.2 is likely to contain a sex‐determining locus (Hearn et al., 2022), and all *barnacle* samples were taken from females while *wave* samples were composed of both males and females. However, the sex‐determining locus was only associated with LGC12.2 in *crab* snails, and sex does not explain why LGC12.1 and LGC12.4 showed *wave–barnacle* differences.

These results should be interpreted with some caution. To reduce noise from geographic variation, samples were filtered to only sites where both ecotypes (or species) were collected. These filters mean that the numbers of both snails and sampling locations varied among the different tests (Table S3). However, the handful of investigated locations still covered a large geographic area around North‐West Europe, which is compatible with the role of inversions in parallel ecotype formation and the inferences of a previous study (Morales et al., 2019). Despite this, LGC14.1/2 was better supported by the model containing an interaction term between the *crab* and *wave* ecotypes and location (Table S6), suggesting that the same inversion arrangement may carry different sets of adaptive alleles for different habitats in different populations. Morales et al. (2019) showed that clusters of *F*_ST_ outliers for several inversions were restricted to Spain and Sweden. Even within a small part of the Swedish coast, associations between inversions, traits and habitat variables are not fully consistent, supporting the hypothesis that an inversion's allelic content can vary among locations (Koch et al., 2022). Overall, this suggests that, although two inversions contribute to parallel phenotypic divergence between *crab* and *wave* ecotypes, their genetic basis may differ across locations (Faria, Johannesson, et al., 2019).

An alternative but non‐mutually exclusive hypothesis is that the selective pressures or habitats differ among locations, reducing the parallelism among environmental contrasts (Bolnick et al., 2018). This is likely if *crab–wave* divergence is also influenced by other environmental features. Shore‐height gradients are a prime candidate (Morales et al., 2019). In Spain and the UK, the *crab* and *wave* ecotypes are differentiated by their height on shore (Butlin et al., 2008), leading to an additional adaptive gradient that is conflated with the *crab–wave* gradient in different directions in the two regions (Morales et al., 2019). Hypothetically, the *wave*–*barnacle* ecotype contrast should also be affected by a shore‐height gradient, as *barnacle* snails typically inhabit lower tidal zones (Reid, 1996, p. 315). In fact, one inversion (LGC12.2) that shows significant differences between *wave* and *barnacle* ecotypes in our study (Figure 4) overlaps with shore‐height candidates from Morales et al. (2019). However, other inversions involved in shore‐height detected by Morales et al. (2019) were not involved in *wave* and *barnacle* divergence in this study, possibly because differences between the *wave* and *barnacle* ecotypes are more multifaceted than shore‐height alone.

Most *L*. *saxatilis* inversions were also polymorphic in its sister species, *L*. *arcana* (Tables 1, 2; Figure S18). Arrangements typically diverge between the species, with one arrangement splitting off from the *L*. *saxatilis* cluster on the second PC axis. We also saw suggestive evidence of inversion polymorphisms in *L*. *compressa* (Table 2; Figure 3 and Figure S18) but did not follow up on these observations because of the limited sample size, making it difficult to implement our other analyses on this species. The existence of shared inversion polymorphisms among species suggests that the arrangements originated before the species split (1.7–0.06 My; Reid et al., 2012). Alternative explanations are that arrangements introgressed between species or inversions evolved repeatedly at the same positions. The repeated evolution scenario has been suggested in humans (Carvalho & Lupski, 2016; Flores et al., 2007), *Drosophila* (Ranz et al., 2007) and deer mice (Harringmeyer & Hoekstra, 2022). However, repeated evolution of inversions is unlikely to apply to these *Littorina* species since the separation of arrangements across species on the same PC axis suggests that they share part of their divergence history, as expected for ancestral polymorphism. It is more challenging to disentangle the patterns of introgression from co‐inherited ancestral variation. Hypothetically, an introgressed arrangement should be less diverged than a shared ancestral arrangement, as the arrangement was more recently shared between species (Fuller et al., 2018; Jay et al., 2018). However, in practice, PCA is a poor guide to seeing divergence differences among arrangements. Furthermore, gene flow has been estimated to be extremely infrequent between the two *Littorina* species (Stankowski et al., 2020). Thus, until introgression and shared ancestry can be properly investigated with tree‐based and demographic analyses, the most parsimonious scenario is that the inversions were co‐inherited from a common ancestor. Assuming common ancestry, these inversions likely appeared before the expansion of *L*. *saxatilis* after the last glacial period (estimated as 0.37 My; Panova et al., 2014). Many inversions in other species appear to be much older than this (Barrón et al., 2019; Coughlan & Willis, 2019; Fuller et al., 2018; Jay et al., 2018; MacGuigan et al., 2022; Todesco et al., 2020), likely maintained under balancing selection preventing fixation or loss in any specific location (Durmaz et al., 2020; Wellenreuther & Bernatchez, 2018). Whether due to shared ancestry or introgression, polymorphic inversions may have provided a ready source of genetic diversity when the snails expanded into new habitats after glacial retreat (Faria, Johannesson, et al., 2019).

Contrary to our predictions, we detected that *L*. *arcana* arrangement frequencies were often closer to *crab* ecotype *L*. *saxatilis* than *wave* ecotype, despite the lower sample sizes (Figure 5; Tables S5,S6). *L*. *arcana* typically inhabits the more wave‐exposed parts of *L. saxatilis'* shore distribution (Reid, 1996). Thus, rare gene flow events are expected to be more common in the wave habitat, and selection is more similar for *wave* ecotype‐adapted alleles or inversion arrangements. Therefore, the arrangement frequency differences we observed suggest either that additional habitat variables influence inversion frequencies or that the sets of adaptive alleles carried by each arrangement vary among species.

Parallel ecotype formation may often be underpinned by polymorphic inversions. With our inversion detection approaches, we have shown that Swedish inversions in *L. saxatilis* are widespread, with some consistently differentiating ecotypes. The majority were also detected in *L*. *arcana*, suggesting that they are ancient polymorphisms that could be maintained by balancing selection. These approaches could be applied to the vast majority of other species that have fragmented reference genomes. Overall, our detection and genotyping demonstrate the important role that inversions play in the diversification of *L. saxatilis* and other closely related species.

## AUTHOR CONTRIBUTIONS

JR, RF & RB conceived this project. SS collated and processed the WGS data. EK constructed the new version of the linkage map. JR conducted the analysis and wrote this paper with the support of RB & RF. All authors reviewed and approved the draft of this paper before submission.

## CONFLICT OF INTEREST STATEMENT

The authors have no conflict of interest to declare.

## Supporting information


Appendix S1.

## Data Availability

All raw sequence data were uploaded to the NCBI short read archive (PRJNA626520). All scripts used for this project are uploaded to GitHub (https://github.com/ja‐Reeve/Littorina_inversion_identification.git).
