# Supplementary material for: Chromosomal inversion polymorphisms are widespread across the species ranges of rough periwinkles (Littorina saxatilis and L. arcana)
Source: Mol Ecol. 2023 Oct 16;33(24):e17160. doi: 10.1111/mec.17160 (PMC11628645; doi:10.1111/mec.17160)
Supplement: Supplementary file 1 — Appendix S1. [file MEC-33-e17160-s001.docx]

**Supplementary material: Chromosomal inversions polymorphisms are widespread across the species ranges of rough periwinkles (*L. saxatilis* and *L. arcana*)**

**Supplementary method 1: consensus map construction**

Two versions of a *Littorina saxatilis* linkage map currently exist. The older version was created from a cross of two *crab* ecotype snails and sequenced with 40,000 capture probes (Westram et al. 2018, Supplementary methods). Henceforth, it will be referred to as the ‘crab map’. The younger version was assembled from 13 F2 families from two crosses of *wave* x *crab* parents (Koch et al. 2021). This later map will be called the ‘crab x wave map’. Both maps were assembled with Lep-MAP (*crab map* = Lep-MAP2 (Rastas et al., 2016); *crab x wave map* = Lep-MAP3 (Rastas 2017)).

Both maps have a problem with ordering markers in some inverted regions (defined in Westram et al. 2021). The parental genotypes were unknown in both maps prior to crossing. If one of the parents was heterozygous for an inversion, map distances were compressed within this inversion, as recombination in inverted regions is supressed when a parent was a heterkaryotype (Coyne et al., 1991). Map compression was very noticeable on LG10 and LG14, as they are almost entirely covered by inversions (Faria et al., 2019; Westram et al., 2021). To improve the resolution of distances in inversions we merged information across maps to generate a more informative ‘consensus map’.

The *consensus map* was built by merging the *crab map* and *crab x wave map* using the R package ‘LPmerge’ (Endelman & Plomion 2014). Before merging markers in inverted regions were filtered to retain those which had the greatest maps distances, and thus the least recombination suppression. LG10 and LG14 had lower marker density and large gaps in the consensus, therefore only *crab map* markers were used for these linkage groups. The average marker position was calculated for each contig. Any markers >2cM from their contig average were removed and a new average was calculated from the remaining markers. These average positions were defined as the *consensus map* positions.

**Supplementary method 2: manual adjustments to inversion genotyping**

K-means clustering could not always identify a number of clusters consistent with the patterns of simple (K = 3) or complex (K = 6) inversions. Often this was because some aspect of geographic distance influenced the variance explained on the first principal component. Each inversion in the Northern saxatilis group and the *arcana*-*saxatilis* comparison, was visually inspected after K-means clustering. Any inversion where the best K ≠ 3 or 6, or where the clustering seemed to be distorted by geographic variation, was manually adjusted. The problems and manual adjustments for each inversion are described below. For step-by-step details see the code at <https://github.com/ja-Reeve/Littorina_inversion_identification/tree/main/R_scripts/6.5_manual_edits_of_inversion_genotype.R>

*Adjustments for Northern saxatilis:*

LGC1.1: K-means found only two clusters because clustering was slightly diagonal on the PCA plot (Figure S19). Since K-means only used PC1 scores for most inversions, the heterokaryotypic cluster had to be demarcated visually. Arrangements were manually adjusted with thresholds ‘AA’ = PC1 < -40, ‘RA’ = -40 < PC1 < -10, and ‘RR’ = PC1 > -10.

LGC2.1: a small clump of four American samples falls between the ‘AA’ and ‘RA’ clusters down the bottom of the PCA plot (Figure S19). One sample, *York_B-1_Ls,* seemed to belong to the ‘RA’ cluster while the others belong to the ‘AA’ cluster. Removing these samples then projecting them back onto the PC axes confirmed this visual assessment.

LGC9.2: the gap between the ‘RA’ and ‘AA’ clusters is unclear (Figure S22). Most of spread on PC2 was from snails sampled from the North Sea. Looking at this plot by region, only samples collected from the Celtic Sea appear to have a pattern consistent with an inversion polymorphism. To verify, the largest group, North Sea samples, was removed resulting in four clusters. PC2 explained the variation in the Celtic Sea while PC1 distinguished the American samples (Figure S22). Considering that the variance explained along PC1 was low and reflects geographic separation, we deemed that the support for this inversion was too insubstantial to include it in the association tests.

*Adjustments for the* arcana-saxatilis *comparisons:*

Note: *L. arcana* and *L. saxatilis* were filtered to keep only samples from locations where both species were collected. For brevity, we will just use the species name in the following section.

LGC4.1: *L.* *saxatilis* forms three clear clusters, which did not align well with *L. arcana* (Figure S21). To improve the alignment, *L. arcana* samples were projected onto a PCA of *L. saxatilis.* Projected *L. arcana* clustered with the right arrangement in *L. saxatilis* (Figure S22). Interesting, there were two clusters within *L. arcana*, with one cluster falling between the right and central *L. saxatilis* clusters. These centralized points were not associated with any sampling site or specific country, and the average heterozygosity was higher than other *L. arcana*. Based on this information, *L. arcana* samples were labelled using the following thresholds: ‘RA’ = 15 < PC1 < 30 & PC2 > -5, all others were ‘AA’.

LGC5.1: *L. arcana* clusters separate on PC2 while *L. saxatilis* clusters are unclear (Figure S22). PC1 only explains a small proportion of the variance among samples in the Northern saxatilis PCA (Figure S20), suggesting that this inversion may have a weak pattern relative to the differences among species. *L. arcana* samples were projected onto a PCA of *L. saxatilis*, and vice versa (Figure S22). When *L. saxatilis* was projected onto *L. arcana*’s PC axes, *L. saxatilis* becomes a small cluster. The reverse occurs for the inverse projection. Since *L. arcana* aligned poorly with both the ‘RR’ and ‘AA’ clusters, it is hard to assign it. Thus, this inversion was not assessed in the species comparisons.

LGC6.1/2: five of the six clusters diagnostic of a complex double inversion are present in the PCA plot of both species (Figure S21), but the clusters in *L. arcana* fall between *L. saxatilis* clusters. Projecting *L. arcana* onto the *L. saxatilis* PC axes, aligned the cluster better between species (Figure S22). One *L. arcana* cluster, which comprised a single outlying sample, still falls between *L. saxatilis* clusters. After looking at average heterozygosity, this sample was assigned to the ‘RR’ cluster. Arrangements for both species were assigned with the following thresholds: ‘RR’ = PC1 < -30; ‘RA_1_’ not found; ‘RA_2_’ = -30 < PC1 < 0; ‘A_1_A_1_’ = PC2 < -30; ‘A_1_A_2_’ = -30 < PC2 < 0; ‘A_2_A_2_’ = PC1 > 12 & PC2 > 5.

LGC14.1: similar to LGC4.1, *L. saxatilis* had three clear clusters, while *L. arcana* formed a cloud of points (Figure S21). Projecting *L. arcana* onto *L. saxatilis* PCA axes showed that there was only one cluster for *L. arcana,* which aligned with ‘AA’ (Figure S22).

LGC14.2: *L. saxatilis* had six clusters and *L. arcana* formed a separate group along the right edge of the PCA plot (Figure S22). This pattern suggests the double inversion is fixed for one arrangement in *L. arcana*. There were no clear clusters in a PCA plot of just *L. arcana*. Instead, samples seemed to be grouped by geographic region. Projecting *L. arcana* onto *L. saxatilis* PCA axes did not improve the clustering, as *L. arcana* still formed a continuous group between two *L. saxatilis* clusters (Figure S22). Further testing of SNPs in the boundary regions also showed similar patterns. Given this complexity and the lack of a clear resolution with these data, LGC14.2 was not assessed in the species comparisons.

**References:**

Coyne, J. A., Aulard, S., & Berry, A. (1991). Lack of underdominance in a naturally occurring pericentric inversion in *Drosophila melanogaster* and its implications for chromosome evolution. *Genetics, 129*, 791-802. https://doi.org/10.1093/genetics/129.3.791

Endelman, J. B., & Plomion, C. (2014). LPmerge: an R package for merging genetic maps by linear programming. *Bioinformatics, 30*(11), 1623-1624. https://doi.org/10.1093/bioinformatics/btu091

Faria, R., Chaube, P., Morales, H. E., Larsson, T., Lemmon, A. R., Lemmon, E. M., Rafajlović, M., Panova, M., Ravinet, M., Johannesson, K., Westram, A. M., & Butlin, R. K. (2019). Multiple chromosomal rearrangements in a hybrid zone between *Littorina saxatilis* ecotypes. *Molecular Ecology, 28*(6), 1375–1393. https://doi.org/10.1111/mec.14972

Koch, E. L., Morales, H. E., Larsson, J., Westram, A. M., Faria, R., Lemmon, A. R., Lemmon, E. M., Johannesson, K., & Butlin, R. K. (2021). Genetic variation for adaptive traits is associated with polymorphic inversions in *Littorina saxatilis*. *Evolution Letters, 5*(3), 196–213. <https://doi.org/10.1002/evl3.227>

Korunes, K. L., & Noor, M. A. F., (2017). Gene conversion and linkage: effects on genome evolution and speciation. *Molecular Ecology, 26*, 351-364. https://doi.org/10.1111/mec.13736

Rastas, P., Calboli, F. C. F., Guo, B., Shikano, T., & Merilä, J. (2016). Construction of ultradense linkage maps with Lep-MAP2: stickleback F_2_ recombinant crosses as an example. *Genome Biology and Evolution, 8*(1), 8-93. https://doi.org/10.1093/gbe/evv250

Rastas, P. (2017). Lep-MAP3: robust linkage mapping even for low-coverage whole genome sequencing data. *Bioinformatics, 33*(23), 3726-3732. <https://doi.org/10.1093/bioinformatics/btx494>

Stankowski, S., Zagrodzka, Z. B., Galindo, J., Montaño-Rendón, M., Faria, R., Mikhailova, N., Blakeslee, A. M. H., Arnason, E., Broquet, T., Morales, H. E., Grahame, J. W., Westram, A. M., Johannesson, K., & Butlin, R. K. (2023) Whole-genome phylogeography of the intertidal snail *Littorina saxatilis*. *Evolutionary Journal of the Linnean Society*. Online Early Access. https://doi.org/10.1093/evolinnean/kzad002

Westram, A. M., Faria, R., Johannesson, K., Butlin, R., & Barton, N. (2022). Inversions and parallel evolution. *Philosophical Transactions of the Royal Society B: Biological Sciences, 377*(1856). https://doi.org/10.1098/rstb.2021.0203

Westram, A. M., Rafajlović, M., Chaube, P., Faria, R., Larsson, T., Panova, M., Ravinet, M., Blomberg, A., Mehlig, B., Johannesson, K., & Butlin, R. (2018). Clines on the seashore: The genomic architecture underlying rapid divergence in the face of gene flow. *Evolution Letters, 2*(4), 297–309. <https://doi.org/10.1002/evl3.74>

**Supplementary tables:**

**Table S1:** Snail sampling details. N_sax_ , N_arc_, and N_comp_ are the number of *Littorina saxatilis*, *L. arcana* and  *L. compressa* collected. ‘-‘ indicates locations outside the species range of *L. arcana* or *L. compressa*. Ramsö and Arsklåvet were called CZA and CZD in Westram et al. 2021. Collector names are abbreviated in the table.

MMR = Mauricio Montaño-Rendón; Sheffield Chlidren’s NHS Foundation Trust, UK

AMW = Anja Marie Westram; Nord University, Norway

KJ = Kerstin Johanesson; University of Gothenburg, Sweden

RB = Roger Butlin; University of Sheffield, UK & University of Gothenburg, Sweden

SS = Sean Stankowski; ISTA, Austria

JL = Jenny Larsson; University of Gothenburg & Chalmers University, Sweden

ZZ = Zuzanna Zagrodzka; University of Sheffield, UK

EA = Einar Árnason; University of Iceland

TB = Tomas Broquet; Station Biologique de Roscoff, France

PK = Petri Kemppainen; University of Helsinki, Finland

NM = Natalia Mikhailova; St. Petersburg State University, Russia

AB = April Blakeslee; East Carolina University, USA

JG = Juan Galindo; University of Vigo, Spain

| **Location** | **Country** | **Latitude** | **Longitude** | **N_sax_** | **N_arc_** | **N_­comp_** | **Collector** |
| --- | --- | --- | --- | --- | --- | --- | --- |
| *Northern samples (*L. saxatilis *= 67;* L.arcana *= 24;* L. compressa *= 4):* | | | | | | | |
| Amble | England (UK) | 55.33578 | -1.56953 | 0 | 1 | 0 | MMR |
| Arsklåvet | Sweden | 58.83091 | 11.13305 | 4 | - | - | AMW, KJ, RB |
| Broad Haven | Wales (UK) | 51.60891 | -4.91878 | 1 | 1 | 0 | MMR |
| Ceann Tra | Ireland | 52.13205 | -10.36071 | 2 | 0 | 1 | MMR |
| Dersingham | England (UK) | 52.86750 | 0.44738 | 2 | - | - | MMR |
| Holyhead | Wales (UK) | 53.29981 | -4.67967 | 12 | 4 | 1 | SS, JL, ZZ |
| Laugarnes | Iceland | 64.15250 | -21.88383 | 2 | - | - | EA |
| Oban | Scotland (UK) | 56.42207 | -5.48392 | 1 | 0 | 0 | MMR |
| Port Saint Mary | Isle of Mann | 54.07602 | -4.73618 | 1 | 0 | 0 | MMR |
| Ramsö | Sweden | 58.82438 | 11.06258 | 4 | - | - | AMW, KJ, RB |
| Ravenscar | England (UK) | 54.41036 | -0.49196 | 7 | 7 | - | SS, AMW, ZZ |
| Roscoff | Brittany (France) | 48.69481 | -4.10734 | 6 | 4 | 1 | TB |
| Saint Abbs | Scotland (UK) | 55.89968 | -2.13004 | 2 | 2 | - | MMR |
| Thornwick | England (UK) | 54.13267 | -0.11503 | 8 | 0 | - | RB |
| Tjärnö | Sweden | 58.88994 | 11.13866 | 2 | - | - | KJ |
| Trondheim Fjord | Norway | 63.55228 | 10.46486 | 3 | 1 | 0 | PK |
| Varanger Fjord | Norway | 70.04039 | 29.58401 | 4 | 4 | 1 | NM |
| White Sea | Russia | 66.33082 | 33.06251 | 2 | - | - | NM |
| York | Maine (USA) | 43.15093 | -70.62182 | 4 | - | - | AB |
| *Iberian samples (*L. saxatilis *= 12):* | | | | | | | |
| Burella | Spain | 43.66556 | -7.35782 | 2 | - | - | JG |
| Centinela | Spain | 42.07786 | -8.89555 | 10 | - | - | JG |

**Table S2:** Tests for clustering of heterozygosity splits along each linkage group for each genetic group. V_obs_ = observed variation among 3cM overlapping windows. V_Perm_ = average variance of 10,000 random draws of variance (± standard deviation). Obs/Perm = ratio of observed to permuted variance. P = empirical P-values, adjusted by a Bejamini-Hochberg correction. Significant results are coloured in green, given an α ≤ 0.05.

| **LG** | **Genetic Group** | **V_Obs_** | **V_Perm_ ± SD** | **Obs/ Perm** | **P-value** | **LG** | **V_Obs_** | **V_Perm_ ± SD** | **Obs/ Perm** | **P-value** |
| --- | --- | --- | --- | --- | --- | --- | --- | --- | --- | --- |
| LG1 | Northern saxatilis | 42.99 | 4.35±1.00 | 9.88 | 0.0001 | LG10 | 19.08 | 4.50±1.69 | 4.24 | 0.0001 |
|  | Iberian saxatilis | 0.88 | 0.77±0.17 | 1.14 | 0.3061 |  | 5.50 | 2.02±0.76 | 2.72 | 0.0031 |
|  | *Littorina arcana* | 8.01 | 1.92±0.44 | 4.18 | 0.0003 |  | 26.67 | 5.66±2.18 | 4.71 | 0.0003 |
| LG2 | Northern saxatilis | 12.18 | 3.84±0.93 | 3.17 | 0.0001 | LG11 | 17.91 | 3.66±1.17 | 4.89 | 0.0001 |
|  | Iberian saxatilis | 5.06 | 1.07±0.25 | 4.72 | 0.0004 |  | 2.22 | 0.62±0.19 | 3.55 | 0.0004 |
|  | *Littorina arcana* | 3.17 | 1.70±0.40 | 1.86 | 0.0077 |  | 8.51 | 1.87±0.59 | 4.55 | 0.0003 |
| LG3 | Northern saxatilis | 5.68 | 1.98±0.51 | 2.86 | 0.0001 | LG12 | 123.10 | 3.66±1.17 | 4.89 | 0.0001 |
|  | Iberian saxatilis | 0.25 | 0.39±0.10 | 0.64 | 0.9698 |  | 9.25 | 1.96±0.62 | 4.72 | 0.0004 |
|  | *Littorina arcana* | 0.84 | 0.78±0.20 | 1.07 | 0.4138 |  | 36.48 | 4.68±1.49 | 7.80 | 0.0003 |
| LG4 | Northern saxatilis | 6.58 | 2.51±0.62 | 2.62 | 0.0001 | LG13 | 3.37 | 1.49±0.43 | 2.26 | 0.0010 |
|  | Iberian saxatilis | 0.42 | 0.28±0.07 | 1.49 | 0.0910 |  | 0.11 | 0.11±0.03 | 0.99 | 0.9394 |
|  | *Littorina arcana* | 1.79 | 1.05±0.26 | 1.70 | 0.0272 |  | 0.60 | 0.59±0.17 | 1.02 | 0.4422 |
| LG5 | Northern saxatilis | 37.54 | 4.67±1.32 | 8.04 | 0.0001 | LG14 | 34.67 | 7.43±3.10 | 4.67 | 0.0001 |
|  | Iberian saxatilis | 1.14 | 0.87±0.24 | 1.32 | 0.1784 |  | 1.92 | 1.18±0.49 | 1.62 | 0.1193 |
|  | *Littorina arcana* | 1.42 | 1.12±0.31 | 1.27 | 0.2028 |  | 4.21 | 2.26±0.94 | 1.86 | 0.0797 |
| LG6 | Northern saxatilis | 74.99 | 6.29±1.73 | 11.93 | 0.0001 | LG15 | 2.23 | 1.24±0.35 | 1.80 | 0.0125 |
|  | Iberian saxatilis | 9.13 | 1.33±0.36 | 6.86 | 0.0004 |  | 0.37 | 0.41±0.11 | 0.89 | 0.7421 |
|  | *Littorina arcana* | 2.11 | 1.56±0.42 | 1.35 | 0.1583 |  | 1.71 | 1.28±0.36 | 1.33 | 0.1711 |
| LG7 | Northern saxatilis | 25.33 | 4.10±1.16 | 6.18 | 0.0001 | LG16 | 1.31 | 1.05±0.29 | 1.25 | 0.1682 |
|  | Iberian saxatilis | 1.18 | 0.69±0.19 | 1.70 | 0.0408 |  | 0.21 | 0.10±0.02 | 2.12 | 0.0300 |
|  | *Littorina arcana* | 10.52 | 2.74±0.76 | 3.84 | 0.0003 |  | 0.70 | 0.50±0.14 | 1.39 | 0.1583 |
| LG8 | Northern saxatilis | 7.43 | 2.49±0.74 | 2.98 | 0.0001 | LG17 | 21.12 | 3.19±0.87 | 6.63 | 0.0001 |
|  | Iberian saxatilis | 0.06 | 0.05±0.01 | 1.06 | 0.9624 |  | 2.05 | 0.74±0.21 | 2.78 | 0.0007 |
|  | *Littorina arcana* | 0.38 | 0.49±0.14 | 0.77 | 0.7982 |  | 0.83 | 0.78±0.22 | 1.05 | 0.4201 |
| LG9 | Northern saxatilis | 98.78 | 8.42±2.35 | 11.73 | 0.0001 |  |  |  |  |  |
|  | Iberian saxatilis | 2.68 | 1.24±0.34 | 2.17 | 0.0036 |  |  |  |  |  |
|  | *Littorina arcana* | 1.96 | 1.35±0.37 | 1.45 | 0.1264 |  |  |  |  |  |

**Table S3:** Inversion arrangement counts for different ecotype and species contrasts. Total arrangement counts are written next to the titles.

| **Crab vs wave (n = 56)** | | | | | | | | | | | | | | | | | | | | | | | | |
| --- | --- | --- | --- | --- | --- | --- | --- | --- | --- | --- | --- | --- | --- | --- | --- | --- | --- | --- | --- | --- | --- | --- | --- | --- |
|  | LGC1.1 | | | | LGC1.2 | | | | LGC2.1 | | | | LGC4.1 | | | | LGC5.1 | | | | LGC7.1 | | | |
| Location | *crab* | | *wave* | | *crab* | | *wave* | | *crab* | | *wave* | | *crab* | | *wave* | | *crab* | | *wave* | | *crab* | | *wave* | |
|  | R | A | R | A | R | A | R | A | R | A | R | A | R | A | R | A | R | A | R | A | R | A | R | A |
| Arsklåvet | 4 | 0 | 4 | 0 | 2 | 2 | 2 | 2 | 3 | 1 | 0 | 4 | 3 | 1 | 4 | 0 | 4 | 0 | 4 | 0 | 2 | 2 | 0 | 4 |
| Ceann Tra | 2 | 0 | 2 | 0 | 2 | 0 | 2 | 0 | 0 | 2 | 1 | 1 | 2 | 0 | 2 | 0 | 1 | 1 | 1 | 1 | 1 | 1 | 1 | 1 |
| Holyhead | 8 | 0 | 8 | 4 | 5 | 3 | 8 | 4 | 1 | 7 | 3 | 9 | 7 | 1 | 8 | 4 | 7 | 1 | 11 | 1 | 1 | 7 | 3 | 9 |
| Ramsö | 2 | 2 | 4 | 0 | 3 | 1 | 3 | 1 | 2 | 2 | 0 | 4 | 2 | 2 | 3 | 1 | 4 | 0 | 4 | 0 | 3 | 1 | 1 | 3 |
| Thornwick | 7 | 1 | 8 | 0 | 6 | 2 | 5 | 3 | 0 | 8 | 0 | 8 | 8 | 0 | 6 | 2 | 7 | 1 | 8 | 0 | 0 | 8 | 1 | 7 |
|  | LGC7.2 | | | | LGC9.1 | | | | LGC10.1 | | | | LGC10.2 | | | | LGC11.1 | | | | LGC12.1 | | | |
| Arsklåvet | 2 | 2 | 0 | 4 | 3 | 4 | 0 | 2 | 2 | 3 | 0 | 4 | 1 | 3 | 0 | 4 | 4 | 0 | 3 | 1 | 4 | 0 | 2 | 2 |
| Ceann Tra | 1 | 1 | 1 | 1 | 2 | 2 | 0 | 2 | 0 | 2 | 1 | 1 | 0 | 2 | 0 | 2 | 2 | 0 | 2 | 0 | 2 | 0 | 2 | 0 |
| Holyhead | 5 | 3 | 3 | 9 | 3 | 5 | 3 | 10 | 2 | 0 | 6 | 6 | 5 | 3 | 5 | 7 | 5 | 3 | 7 | 5 | 5 | 3 | 10 | 2 |
| Ramsö | 3 | 1 | 2 | 2 | 3 | 4 | 0 | 2 | 2 | 3 | 0 | 4 | 1 | 3 | 0 | 4 | 2 | 2 | 2 | 2 | 4 | 0 | 2 | 2 |
| Thornwick | 4 | 4 | 5 | 3 | 4 | 6 | 2 | 2 | 6 | 8 | 2 | 6 | 0 | 8 | 3 | 5 | 7 | 1 | 4 | 4 | 6 | 2 | 2 | 6 |
|  | LGC12.2 | | | | LGC12.4 | | | | LGC14.1 | | | | LGC17.1 | | | |  | | | |  |  |  |  |
| Arsklåvet | 2 | 2 | 2 | 2 | 3 | 1 | 2 | 2 | 4 | 0 | 0 | 4 | 4 | 0 | 3 | 1 |  |  |  |  |  |  |  |  |
| Ceann Tra | 0 | 2 | 0 | 2 | 0 | 2 | 0 | 2 | 2 | 0 | 2 | 0 | 2 | 0 | 2 | 0 |  |  |  |  |  |  |  |  |
| Holyhead | 8 | 0 | 6 | 6 | 5 | 3 | 5 | 7 | 5 | 3 | 5 | 7 | 8 | 0 | 9 | 3 |  |  |  |  |  |  |  |  |
| Ramsö | 1 | 3 | 2 | 2 | 4 | 0 | 0 | 4 | 3 | 1 | 2 | 2 | 4 | 0 | 3 | 1 |  |  |  |  |  |  |  |  |
| Thornwick | 3 | 5 | 6 | 2 | 3 | 5 | 2 | 6 | 8 | 0 | 1 | 7 | 8 | 0 | 8 | 0 |  |  |  |  |  |  |  |  |
|  | LGC6.1/2 | | | | | | LGC14.2 | | | | | |  |  |  |  |  |  |  |  |  |  |  |  |
|  | R | A_1_ | A_2_ | R | A_1_ | A_2_ | R | A_1_ | A_2_ | R | A_1_ | A_2_ |  |  |  |  |  |  |  |  |  |  |  |  |
| Arsklåvet | 3 | 0 | 1 | 0 | 1 | 3 | 4 | 0 | 0 | 0 | 4 | 0 |  |  |  |  |  |  |  |  |  |  |  |  |
| Ceann Tra | 2 | 0 | 0 | 2 | 0 | 0 | 2 | 0 | 0 | 2 | 0 | 0 |  |  |  |  |  |  |  |  |  |  |  |  |
| Holyhead | 7 | 0 | 1 | 6 | 4 | 2 | 5 | 2 | 1 | 5 | 4 | 3 |  |  |  |  |  |  |  |  |  |  |  |  |
| Ramsö | 3 | 0 | 1 | 0 | 1 | 3 | 3 | 0 | 1 | 2 | 2 | 0 |  |  |  |  |  |  |  |  |  |  |  |  |
| Thornwick | 6 | 1 | 1 | 1 | 2 | 5 | 8 | 0 | 0 | 1 | 5 | 2 |  |  |  |  |  |  |  |  |  |  |  |  |
| **Wave vs barnacle (n = 30)** | | | | | | | | | | | | | | | | | | | | | | | | |
|  | LGC1.1 | | | | LGC1.2 | | | | LGC2.1 | | | | LGC4.1 | | | | LGC5.1 | | | | LGC7.1 | | | |
| Location | *barn* | | *wave* | | *barn* | | *wave* | | *barn* | | *wave* | | *barn* | | *wave* | | *barn* | | *wave* | | *barn* | | *wave* | |
|  | R | A | R | A | R | A | R | A | R | A | R | A | R | A | R | A | R | A | R | A | R | A | R | A |
| Holyhead | 4 | 0 | 8 | 4 | 4 | 0 | 8 | 4 | 0 | 4 | 3 | 9 | 4 | 0 | 8 | 4 | 4 | 0 | 11 | 1 | 0 | 4 | 3 | 9 |
| Ravenscar | 4 | 0 | 10 | 0 | 4 | 0 | 4 | 6 | 0 | 4 | 2 | 8 | 4 | 0 | 7 | 3 | 4 | 0 | 10 | 0 | 0 | 4 | 0 | 10 |
|  | LGC7.2 | | | | LGC9.1 | | | | LGC10.1 | | | | LGC10.2 | | | | LGC11.1 | | | | LGC12.1 | | | |
| Holyhead | 0 | 4 | 3 | 9 | 4 | 4 | 0 | 10 | 2 | 4 | 6 | 6 | 0 | 4 | 5 | 7 | 4 | 0 | 7 | 5 | 4 | 0 | 10 | 2 |
| Ravenscar | 0 | 4 | 4 | 6 | 3 | 4 | 0 | 2 | 8 | 4 | 4 | 6 | 0 | 4 | 3 | 7 | 4 | 0 | 6 | 4 | 4 | 0 | 2 | 8 |
|  | LGC12.2 | | | | LGC12.4 | | | | LGC14.1 | | | | LGC17.1 | | | |  | | | |  |  |  |  |
| Holyhead | 0 | 4 | 6 | 6 | 0 | 4 | 5 | 7 | 0 | 4 | 5 | 7 | 2 | 2 | 9 | 3 |  |  |  |  |  |  |  |  |
| Ravenscar | 0 | 4 | 9 | 1 | 0 | 4 | 6 | 4 | 0 | 4 | 4 | 6 | 0 | 4 | 8 | 2 |  |  |  |  |  |  |  |  |
|  | LGC6.1/2 | | | | | | LGC14.2 | | | | | |  |  |  |  |  |  |  |  |  |  |  |  |
|  | R | A_1_ | A_2_ | R | A_1_ | A_2_ | R | A_1_ | A_2_ | R | A_1_ | A_2_ |  |  |  |  |  |  |  |  |  |  |  |  |
| Holyhead | 0 | 4 | 0 | 6 | 4 | 2 | 0 | 4 | 0 | 5 | 4 | 3 |  |  |  |  |  |  |  |  |  |  |  |  |
| Ravenscar | 0 | 4 | 0 | 2 | 4 | 4 | 0 | 4 | 0 | 4 | 3 | 3 |  |  |  |  |  |  |  |  |  |  |  |  |
| **Crab vs *L. arcana* (n = 16)** | | | | | | | | | | | | | | | | | | | | | | | | |
|  | LGC1.1 | | | | LGC1.2 | | | | LGC2.1 | | | | LGC4.1 | | | | LGC7.1 | | | | LGC7.2 | | | |
| Location | *crab* | | *arcana* | | *crab* | | *arcana* | | *crab* | | *arcana* | | *crab* | | *arcana* | | *crab* | | *arcana* | | *crab* | | *arcana* | |
|  | R | A | R | A | R | A | R | A | R | A | R | A | R | A | R | A | R | A | R | A | R | A | R | A |
| Holyhead | 8 | 0 | 5 | 3 | 5 | 3 | 1 | 7 | 7 | 1 | 1 | 7 | 7 | 1 | 0 | 8 | 7 | 1 | 0 | 8 | 5 | 3 | 8 | 0 |
|  | LGC9.1 | | | | LGC9.2 | | | | LGC10.1 | | | | LGC10.2 | | | | LGC11.1 | | | | LGC12.1 | | | |
| Holyhead | 5 | 3 | 8 | 0 | 7 | 1 | 6 | 2 | 8 | 0 | 5 | 3 | 5 | 3 | 7 | 1 | 5 | 3 | 1 | 7 | 3 | 5 | 3 | 5 |
|  | LGC12.4 | | | | LGC14.1 | | | | LGC17.1 | | | | LGC6.1/2 | | | | | |  |  |  |  |  |  |
|  |  | | | |  | | | |  | | | | R | A_1_ | A_2_ | R | A_1_ | A_2_ |  |  |  |  |  |  |
| Holyhead | 5 | 3 | 7 | 1 | 5 | 3 | 0 | 8 | 8 | 0 | 8 | 0 | 7 | 0 | 1 | 0 | 1 | 7 |  |  |  |  |  |  |
| **Wave vs *L. arcana* (n = 56)** | | | | | | | | | | | | | | | | | | | | | | | | |
|  | LGC1.1 | | | | LGC1.2 | | | | LGC2.1 | | | | LGC4.1 | | | | LGC7.1 | | | | LGC7.2 | | | |
| Location | *wave* | | *arcana* | | *wave* | | *arcana* | | *wave* | | *arcana* | | *wave* | | *arcana* | | *wave* | | *arcana* | | *wave* | | *arcana* | |
|  | R | A | R | A | R | A | R | A | R | A | R | A | R | A | R | A | R | A | R | A | R | A | R | A |
| Broad Haven | 2 | 0 | 1 | 1 | 0 | 2 | 1 | 1 | 0 | 2 | 0 | 2 | 1 | 1 | 0 | 2 | 1 | 1 | 2 | 0 | 0 | 2 | 0 | 2 |
| Holyhead | 8 | 4 | 5 | 3 | 8 | 4 | 1 | 7 | 9 | 3 | 1 | 7 | 8 | 4 | 0 | 8 | 9 | 3 | 0 | 8 | 3 | 9 | 8 | 0 |
| Ravenscar | 10 | 0 | 14 | 0 | 4 | 6 | 3 | 11 | 8 | 2 | 7 | 7 | 7 | 3 | 3 | 11 | 10 | 0 | 11 | 3 | 4 | 6 | 11 | 3 |
| St. Abbs | 3 | 1 | 2 | 2 | 4 | 0 | 0 | 4 | 2 | 2 | 0 | 4 | 4 | 0 | 1 | 3 | 3 | 1 | 1 | 3 | 0 | 4 | 3 | 1 |
|  | LGC9.1 | | | | LGC9.2 | | | | LGC10.1 | | | | LGC10.2 | | | | LGC11.1 | | | | LGC12.1 | | | |
| Broad Haven | 1 | 1 | 2 | 0 | 2 | 0 | 2 | 0 | 1 | 1 | 2 | 0 | 0 | 2 | 2 | 0 | 0 | 2 | 0 | 2 | 0 | 2 | 0 | 2 |
| Holyhead | 9 | 3 | 8 | 0 | 12 | 0 | 6 | 2 | 6 | 6 | 5 | 3 | 5 | 7 | 7 | 1 | 7 | 5 | 1 | 7 | 2 | 10 | 3 | 5 |
| Ravenscar | 5 | 5 | 13 | 1 | 10 | 0 | 14 | 0 | 4 | 6 | 5 | 9 | 3 | 7 | 3 | 11 | 6 | 4 | 9 | 5 | 8 | 2 | 4 | 10 |
| St. Abbs | 0 | 4 | 3 | 1 | 4 | 0 | 0 | 4 | 1 | 3 | 4 | 0 | 0 | 4 | 4 | 0 | 3 | 1 | 1 | 3 | 0 | 4 | 2 | 2 |
|  | LGC12.4 | | | | LGC14.1 | | | | LGC17.1 | | | | LGC6.1/2 | | | | | |  |  |  |  |  |  |
|  |  |  |  |  |  |  |  |  |  |  |  |  | R | A_1_ | A_2_ | R | A_1_ | A_2_ |  |  |  |  |  |  |
| Broad Haven | 2 | 0 | 0 | 2 | 1 | 1 | 0 | 2 | 2 | 0 | 2 | 0 | 1 | 0 | 1 | 0 | 1 | 1 |  |  |  |  |  |  |
| Holyhead | 5 | 7 | 7 | 1 | 5 | 7 | 0 | 8 | 9 | 3 | 8 | 0 | 6 | 4 | 2 | 0 | 1 | 7 |  |  |  |  |  |  |
| Ravenscar | 6 | 4 | 7 | 7 | 4 | 6 | 0 | 14 | 8 | 2 | 14 | 0 | 2 | 4 | 4 | 0 | 2 | 12 |  |  |  |  |  |  |
| St. Abbs | 1 | 3 | 2 | 2 | 4 | 0 | 0 | 4 | 1 | 3 | 4 | 0 | 2 | 0 | 2 | 0 | 1 | 3 |  |  |  |  |  |  |

**Table S4:** Logistic regression results for the crab-wave and wave-barnacle contrasts of the Northern saxatilis genetic group. Three models were run per inversion; Null: the location effect, Eco: location + ecotype effects, and Int: the interaction effect. AIC = Akaike information criteria. R^2^ = Cohen’s pseudo R^2^. Dev = deviance. df = degrees of freedom. P-value = P-value with a Benjamini-Hochberg adjustment. Bold AIC indicates the best model and significant P-values are highlighted in green.

| Inv | Model | *crab-wave* (n=52) | | | | | *wave-barnacle* (n=30) | | | | |
| --- | --- | --- | --- | --- | --- | --- | --- | --- | --- | --- | --- |
|  |  | AIC | R^2^ | Dev | df | P-value | AIC | R^2^ | Dev | df | P-value |
|  | *Eco* | 28.34 | 7.54e^-4^ | 7.27e^-3^ | 1 | 1.00 | 8.87 | 1.00 | 2.72 | 1 | 0.30 |
| LGC1.1 | *Int* | 26.70 | 1.00 | 9.64 | 4 | 0.70 | 10.87 | 1.00 | 2.81e^-10^ | 1 | 1.00 |
|  | *Null* | **26.34** |  |  |  |  | **9.59** |  |  |  |  |
| LGC1.2 | *Eco* | 29.94 | 0.10 | 0.03 | 1 | 1.00 | **11.63** | 1.00 | 8.38 | 1 | 0.07 |
|  | *Int* | 37.64 | 1.00 | 0.30 | 4 | 1.00 | 13.63 | 1.00 | 4.20e^-11^ | 1 | 1.00 |
|  | *Null* | **27.97** |  |  |  |  | 18.01 |  |  |  |  |
| LGC2.1 | *Eco* | 32.32 | 0.09 | 1.09 | 1 | 1.00 | 11.10 | 1.00 | 3.42 | 1 | 0.26 |
|  | *Int* | 29.65 | 1.00 | 10.67 | 4 | 0.55 | 13.10 | 1.00 | 2.66e^-12^ | 1 | 1.00 |
|  | *Null* | **31.41** |  |  |  |  | **12.53** |  |  |  |  |
| LGC4.1 | *Eco* | 30.30 | 0.08 | 0.50 | 1 | 1.00 | **11.51** | 1.00 | 5.05 | 1 | 0.13 |
|  | *Int* | 32.48 | 1.00 | 5.82 | 4 | 1.00 | 13.51 | 1.00 | 8.08e^-13^ | 1 | 1.00 |
|  | *Null* | **28.80** |  |  |  |  | 14.56 |  |  |  |  |
| LGC5.1 | *Eco* | 21.44 | 0.34 | 0.53 | 1 | 1.00 | 7.91 | 1.00 | 0.60 | 1 | 0.44 |
|  | *Int* | 28.43 | 1.00 | 1.01 | 4 | 1.00 | 9.91 | 1.00 | 1.66e^-10^ | 1 | 1.00 |
|  | *Null* | **19.97** |  |  |  |  | **6.51** |  |  |  |  |
| LGC6.1/2 | See Table S6 | | | | | | | | | | |
| LGC7.1 | *Eco* | 33.74 | 0.05 | 0.38 | 1 | 1.00 | 8.71 | 1.00 | 1.95 | 1 | 0.33 |
|  | *Int* | 34.63 | 1.00 | 7.11 | 4 | 1.00 | 10.71 | 1.00 | 2.44e^-10^ | 1 | 1.00 |
|  | *Null* | **31.12** |  |  |  |  | **8.66** |  |  |  |  |
| LGC7.2 | *Eco* | 35.44 | 0.34 | 2.44 | 1 | 1.00 | **11.47** | 1.00 | 5.24 | 1 | 0.13 |
|  | *Int* | 38.79 | 1.00 | 4.65 | 4 | 1.00 | 13.47 | 1.00 | 1.64e^-11^ | 1 | 1.00 |
|  | *Null* | **35.88** |  |  |  |  | 14.71 |  |  |  |  |
| LGC9.1 | *Eco* | 30.85 | 0.67 | 3.22 | 1 | 1.00 | **15.75** | 0.73 | 6.69 | 1 | 0.12 |
|  | *Int* | 37.28 | 1.00 | 1.57 | 4 | 1.00 | 15.24 | 1.00 | 2.51 | 1 | 1.00 |
|  | *Null* | **32.07** |  |  |  |  | 20.43 |  |  |  |  |
| LGC9.2 | Not tested due to uncertain clustering | | | | | | | | | | |
| LGC10.1 | *Eco* | 35.99 | 0.12 | 1.80 | 1 | 1.00 | **11.74** | 1.00 | 7.83 | 1 | 0.08 |
|  | *Int* | **30.15** | 1.00 | 13.84 | 4 | 0.16 | 13.74 | 1.00 | 5.76e^-12^ | 1 | 1.00 |
|  | *Null* | 35.79 |  |  |  |  | 17.57 |  |  |  |  |
| LGC10.2 | *Eco* | 32.12 | 0.01 | 0.11 | 1 | 1.00 | **11.59** | 1.00 | 5.91 | 1 | 0.12 |
|  | *Int* | 31.47 | 1.00 | 8.65 | 4 | 0.91 | 13.59 | 1.00 | 8.97e^-12^ | 1 | 1.00 |
|  | *Null* | **30.23** |  |  |  |  | 15.50 |  |  |  |  |
| LGC11.1 | *Eco* | 30.12 | 0.42 | 1.80 | 1 | 1.00 | **11.72** | 1.00 | 6.87 | 1 | 0.12 |
|  | *Int* | 35.60 | 1.00 | 2.52 | 4 | 1.00 | 13.72 | 1.00 | 1.68e^-13^ | 1 | 1.00 |
|  | *Null* | **29.92** |  |  |  |  | 16.58 |  |  |  |  |
| LGC12.1 | *Eco* | **34.23** | 0.29 | 3.52 | 1 | 0.97 | **10.83** | 1.00 | 10.36 | 1 | 0.02 |
|  | *Int* | 33.56 | 1.00 | 8.67 | 4 | 0.91 | 12.83 | 1.00 | 0.00 | 1 | 1.00 |
|  | *Null* | 35.74 |  |  |  |  | 19.19 |  |  |  |  |
| LGC12.2 | *Eco* | 38.09 | 5.06e^-3^ | 0.05 | 1 | 1.00 | **10.87** | 1.00 | 0.16 | 1 | 1.15e^-3^ |
|  | *Int* | 35.46 | 1.00 | 10.63 | 4 | 0.55 | 12.87 | 1.00 | 1.47e^-10^ | 1 | 1.00 |
|  | *Null* | **36.14** |  |  |  |  | 25.16 |  |  |  |  |
| LGC12.3 | Not tested due to uncertain clustering | | | | | | | | | | |
| LGC12.4 | *Eco* | **33.40** | 0.42 | 5.41 | 1 | 0.34 | **11.72** | 1.00 | 9.24 | 1 | 0.04 |
|  | *Int* | 34.04 | 1.00 | 7.36 | 4 | 1.00 | 13.72 | 1.00 | 1.93e^-11^ | 1 | 1.00 |
|  | *Null* | 36.80 |  |  |  |  | 18.95 |  |  |  |  |
| LGC14.1 | *Eco* | 34.85 | 0.58 | 16.57 | 1 | 9.37e^-4^ | **11.72** | 1.00 | 6.87 | 1 | 0.12 |
|  | *Int* | **31.04** | 1.00 | 11.80 | 4 | 0.38 | 13.72 | 1.00 | 1.64e^-13^ | 1 | 1.00 |
|  | *Null* | 49.42 |  |  |  |  | 16.58 |  |  |  |  |
| LGC14.2 | See Table S6 | | | | | | | | | | |
| LGC14.3 | Not tested due to uncertain clustering | | | | | | | | | | |
| LGC17.1 | *Eco* | **18.16** | 1.00 | 6.47 | 1 | 0.20 | **16.20** | 0.68 | 6.81 | 1 | 0.12 |
|  | *Int* | 26.16 | 1.00 | 0.00 | 4 | 1.00 | 15.07 | 1.00 | 3.13 | 1 | 1.00 |
|  | *Null* | 22.63 |  |  |  |  | 21.01 |  |  |  |  |

**Table S5:** Logistic regression results for *Littorina saxatilis* – *L. arcana* contrasts. *L. saxatilis* was separated into crab and wave ecotypes. Three models were run *per* inversion; Null: the location effect, Eco: location + ecotype effects, and Int: the interaction effect. *Crab-arcana* contrasts were limited to a single location. AIC = Akaike information criteria. R^2^ = *Cohen*’s pseudo R^2^. Dev = deviance. df = degrees of freedom. P-value = P-value with a Benjamini-Hochberg adjustment. Bold AIC indicates the best model and significant P-values are highlighted in green.

| Inv | Model | *crab-arcana* (n=16) | | | | | *wave-arcana* (n=56) | | | | |
| --- | --- | --- | --- | --- | --- | --- | --- | --- | --- | --- | --- |
|  |  | AIC | R^2^ | Dev | df | P-value | AIC | R^2^ | Dev | df | P-value |
| LGC1.1 | *Eco* | **6.53** | 0 | 4.86 | 1 | 0.33 | 21.96 | 0.36 | 0.82 | 1 | 1.00 |
|  | *Int* | **-** | - | - | - | - | 26.48 | 1.00 | 1.48 | 3 | 1.00 |
|  | *Null* | 9.39 |  |  |  |  | **20.78** |  |  |  |  |
| LGC1.2 | *Eco* | **8.40** | 0 | 4.56 | 1 | 0.33 | 32.66 | 0.45 | 8.99 | 1 | 0.02 |
|  | *Int* | - | - | - | - | - | **27.64** | 1.00 | 11.02 | 3 | 0.19 |
|  | *Null* | 10.96 |  |  |  |  | 39.65 |  |  |  |  |
| LGC2.1 | *Eco* | **7.74** | 0 | 10.12 | 1 | 0.02 | **23.94** | 0.87 | 12.11 | 1 | 4.51e^-3^ |
|  | *Int* | - | - | - | - | - | 28.06 | 1.00 | 1.88 | 3 | 1.00 |
|  | *Null* | 15.86 |  |  |  |  | 34.05 |  |  |  |  |
|  | *Eco* | **5.87** | 0 | 15.90 | 1 | 1.13e^-3^ | **24.54** | 0.87 | 22.13 | 1 | 4.08e^-5^ |
| LGC4.1 | *Int* | - | - | - | - | - | 27.38 | 1.00 | 3.16 | 3 | 1.00 |
|  | *Null* | 19.77 |  |  |  |  | 44.67 |  |  |  |  |
| LGC5.1 | Not tested due to uncertain clustering | | | | | | | | | | |
| LGC6.1/2 | See Table S6 | | | | | | | | | | |
| LGC7.1 | *Eco* | **5.87** | 0 | 15.90 | 1 | 1.13e^-3^ | 28.78 | 0.60 | 12.90 | 1 | 3.28e^-3^ |
|  | *Int* | - | - | - | - | - | **26.30** | 1.00 | 8.48 | 3 | 0.48 |
|  | *Null* | 19.77 |  |  |  |  | 39.69 |  |  |  |  |
| LGC7.2 | *Eco* | **6.53** | 0 | 4.86 | 1 | 0.33 | **24.52** | 0.81 | 19.29 | 1 | 1.68e^-4^ |
|  | *Int* | - | - | - | - | - | 25.95 | 1.00 | 4.57 | 3 | 1.00 |
|  | *Null* | 9.39 |  |  |  |  | 41.82 |  |  |  |  |
| LGC9.1 | *Eco* | **6.53** | 0 | 4.86 | 1 | 0.33 | **21.90** | 0.92 | 15.80 | 1 | 9.15e^-4^ |
|  | *Int* | - | - | - | - | - | 26.55 | 1.00 | 1.35 | 3 | 1.00 |
|  | *Null* | 9.39 |  |  |  |  | 35.70 |  |  |  |  |
| LGC9.2 | *Eco* | 8.20 | 0 | 0.42 | 1 | 1.00 | **12.33** | 1.00 | 15.10 | 1 | 1.23e^-3^ |
|  | *Int* | - | - | - | - | - | 18.33 | 1.00 | 0 | 3 | 1.00 |
|  | *Null* | **6.62** |  |  |  |  | 25.43 |  |  |  |  |
| LGC10.1 | *Eco* | **6.53** | 0 | 4.86 | 1 | 0.33 | 30.86 | 0.21 | 1.74 | 1 | 0.75 |
|  | *Int* | - | - | - | - | - | 30.44 | 1.00 | 6.42 | 3 | 1.00 |
|  | *Null* | 9.39 |  |  |  |  | **30.60** |  |  |  |  |
| LGC10.2 | *Eco* | 8.40 | 0 | 1.38 | 1 | 1.00 | 33.78 | 0.37 | 7.89 | 1 | 0.03 |
|  | *Int* | - | - | - | - | - | **26.22** | 1.00 | 13.57 | 3 | 0.06 |
|  | *Null* | **7.79** |  |  |  |  | 39.67 |  |  |  |  |
| LGC11.1 | *Eco* | **8.40** | 0 | 4.56 | 1 | 0.33 | 27.86 | 0.44 | 2.95 | 1 | 0.43 |
|  | *Int* | - | - | - | - | - | 30.08 | 1.00 | 3.78 | 3 | 1.00 |
|  | *Null* | 10.96 |  |  |  |  | **28.81** |  |  |  |  |
| LGC12.1 | *Eco* | 9.07 | 0 | 1.33e^-15^ | 1 | 1.00 | 32.95 | 0.03 | 0.37 | 1 | 1.00 |
|  | *Int* | - | - | - | - | - | **28.26** | 1.00 | 10.69 | 3 | 0.20 |
|  | *Null* | **7.07** |  |  |  |  | 31.32 |  |  |  |  |
| LGC12.2 | Not tested due to sex-chromosome bias | | | | | | | | | | |
| LGC12.3 | Not tested due to uncertain clustering | | | | | | | | | | |
| LGC12.4 | *Eco* | 8.40 | 0 | 1.38 | 1 | 1.00 | 34.94 | 0.03 | 0.38 | 1 | 1.00 |
|  | *Int* | - | - | - | - | - | **30.40** | 1.00 | 10.54 | 3 | 0.20 |
|  | *Null* | **7.79** |  |  |  |  | 33.31 |  |  |  |  |
|  | *Eco* | **6.53** | 0 | 9.29 | 1 | 0.03 | **17.10** | 1.00 | 27.18 | 1 | 3.16e^-6^ |
| LGC14.1 | *Int* | - | - | - | - | - | 23.10 | 1.00 | 0 | 3 | 1.00 |
|  | *Null* | 13.82 |  |  |  |  | 42.28 |  |  |  |  |
| LGC14.2 | Not tested due to uncertain clustering | | | | | | | | | | |
| LGC14.3 | Not tested due to uncertain clustering | | | | | | | | | | |
| LGC17.1 | *Eco* | 4.00 | 0 | 0 | 1 | 1.00 | **16.83** | 1.00 | 13.26 | 1 | 2.98e^-3^ |
|  | *Int* | - | - | - | - | - | 22.83 | 1.00 | 0 | 3 | 1.00 |
|  | *Null* | **2.00** |  |  |  |  | 28.09 |  |  |  |  |

**Table S6:** Logistic regression results of the complex double inversions; LGC6.1/2 and LGC14.2. Analyses were run twice, firstly combining the two alternate arrangements (A_1_ + A_2_ = A) before contrasting with the R arrangement. Secondly, the A_1_ and A_2_ arrangement were compared to determine if the ecotype effect is present in the alternate arrangements. Three models were run per inversion; Null: the location effect, Eco: location + ecotype effects, and Int: the interaction effect. Crab-arcana contrasts were limited to a single location. AIC = Akaike information criteria. R^2^ = Cohen’s pseudo R^2^. Dev = deviance. df = degrees of freedom. P-value = P-value with a Benjamini-Hochberg adjustment. Bold AIC indicates the best model and significant P-values are highlighted in green.

|  |  | *crab-wave* (n=52) | | | | | *wave-barnacle* (n=30) | | | | |
| --- | --- | --- | --- | --- | --- | --- | --- | --- | --- | --- | --- |
| Inv | Model | AIC | R^2^ | Dev | df | P-value | AIC | R^2^ | Dev | df | P-value |
| LGC6.1/2  (R vs. A) | *Eco* | **26.89** | 0.89 | 19.92 | 1 | 1.70e^-4^ | **11.37** | 1.00 | 6.01 | 1 | 0.12 |
|  | *Int* | 32.50 | 1.00 | 2.39 | 4 | 1.00 | 13.37 | 1.00 | 6.43e^-11^ | 1 | 1.00 |
|  | *Null* | 44.81 |  |  |  |  | 15.38 |  |  |  |  |
| LGC6.1/2  (A_1_ vs. A_2_) | *Eco* | 22.06 | 1.00 | 6.56 | 1 | 1.00 | **10.82** | 1.00 | 6.56 | 1 | 0.12 |
|  | *Int* | 25.35 | 1.00 | 1.49e^-11^ | 1 | 1.00 | 12.82 | 1.00 | 1.49e^-11^ | 1 | 1.00 |
|  | *Null* | **20.59** |  |  |  |  | 15.37 |  |  |  |  |
| LGC14.2  (R vs. A) | *Eco* | 34.85 | 0.58 | 16.57 | 1 | 9.3e^-4^ | **11.72** | 1.00 | 6.87 | 1 | 0.12 |
|  | *Int* | **31.04** | 1.00 | 11.80 | 4 | 0.38 | 13.72 | 1.00 | 1.64e^-13^ | 1 | 1.00 |
|  | *Null* | 49.42 |  |  |  |  | 16.58 |  |  |  |  |
| LGC14.2  (A_1_ vs. A_2_) | *Eco* | 19.93 | 1.00 | 6.87 | 1 | 0.57 | **10.78** | 1.00 | 7.23 | 1 | 0.11 |
|  | *Int* | 18.36 | 1.00 | 1.64e^-13^ | 1 | 0.82 | 12.78 | 1.00 | 2.57e^-12^ | 1 | 1.00 |
|  | *Null* | **18.26** |  |  |  |  | 16.01 |  |  |  |  |
|  |  |  | | | | |  | | | | |
|  |  | *crab-arcana* (n=16) | | | | | *wave-arcana* (n=56) | | | | |
| Inv | Model | AIC | R^2^ | Dev | df | P-value | AIC | R^2^ | Dev | df | P-value |
| LGC6.1/2  (R vs. A) | *Eco* | **5.87** | 0 | 15.90 | 1 | 1.13e^-3^ | **18.72** | 1.00 | 16.74 | 1 | 6.01e^-4^ |
|  | *Int* | - | - | - | - | - | 24.72 | 1.00 | 1.73e^-11^ | 3 | 1.00 |
|  | *Null* | 19.77 |  |  |  |  | 33.46 |  |  |  |  |
| LGC6.1/2  (A_1_ vs. A_2_) | *Eco* | 5.87 | 0 | 0.25 | 1 | 1.00 | **27.82** | 0.43 | 4.18 | 1 | 0.25 |
|  | *Int* | - | - | - | - | - | 28.26 | 1.00 | 5.56 | 3 | 1.00 |
|  | *Null* | **4.12** |  |  |  |  | 30.00 |  |  |  |  |

**Supplementary figures:**

**
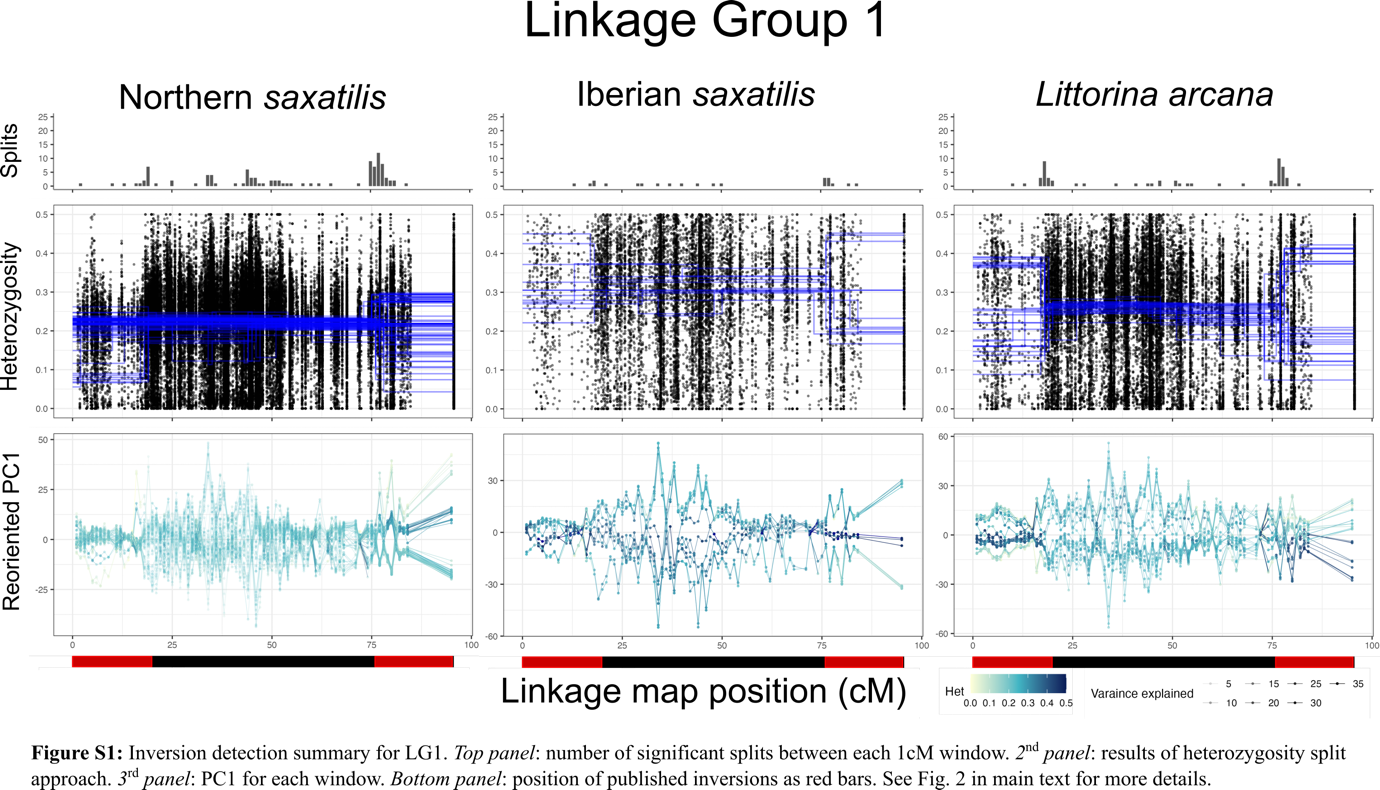
Figure S1:** Inversion detection summary for LG1. *Top panel:* number of significant splits between each 1cM window. *2^nd^ panel*: results of heterozygosity split approach. *3^rd^ panel*: PC1 for each window. *Bottom panel*: position of published inversions as red bars. See Figure 2 in the main text for more details.


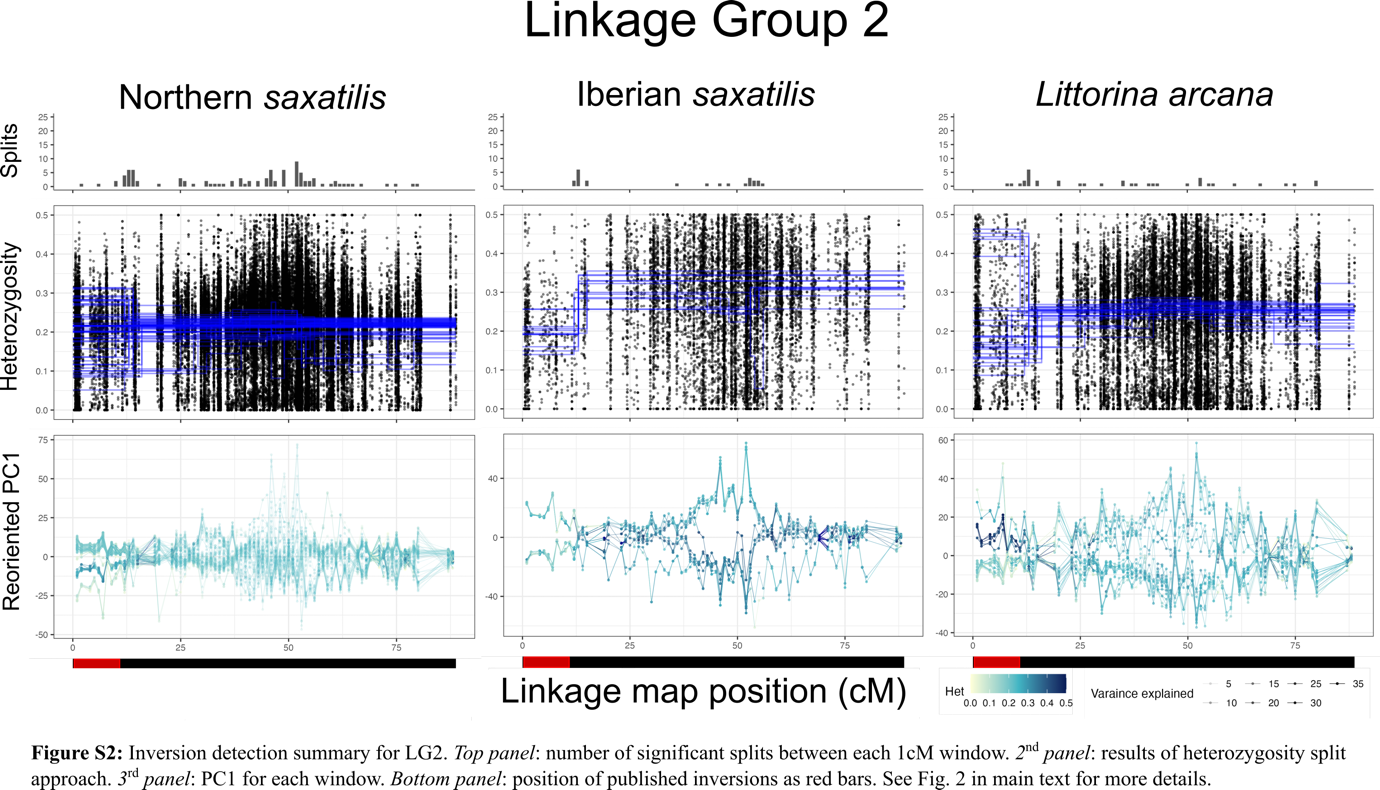
**Figure S2:** Inversion detection summary for LG2. *Top panel:* number of significant splits between each 1cM window. *2^nd^ panel*: results of heterozygosity split approach. *3^rd^ panel*: PC1 for each window. *Bottom panel*: position of published inversions as red bars. See Figure 2 in the main text for more details.


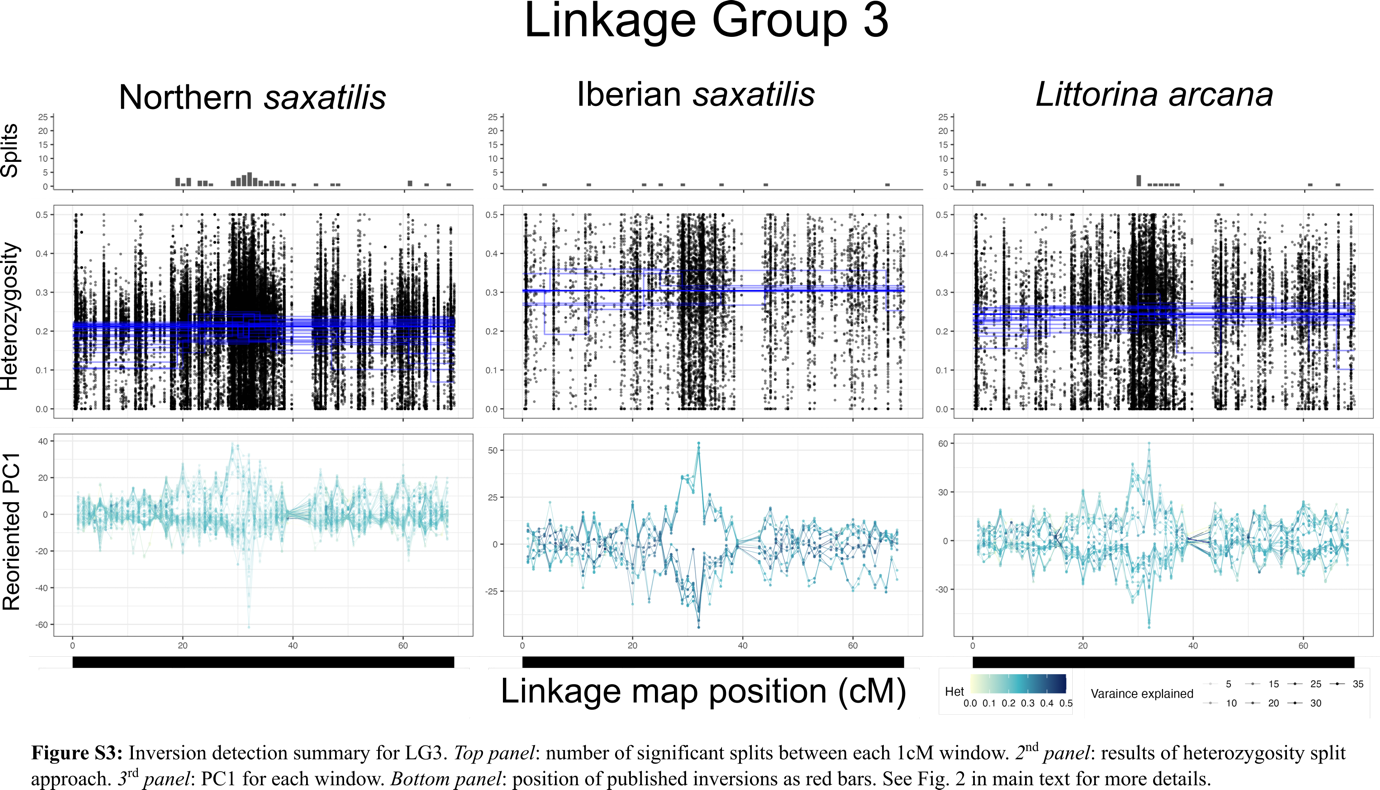
**Figure S3:** Inversion detection summary for LG3. *Top panel:* number of significant splits between each 1cM window. *2^nd^ panel*: results of heterozygosity split approach. *3^rd^ panel*: PC1 for each window. *Bottom panel*: position of published inversions as red bars. See Figure 2 in the main text for more details.


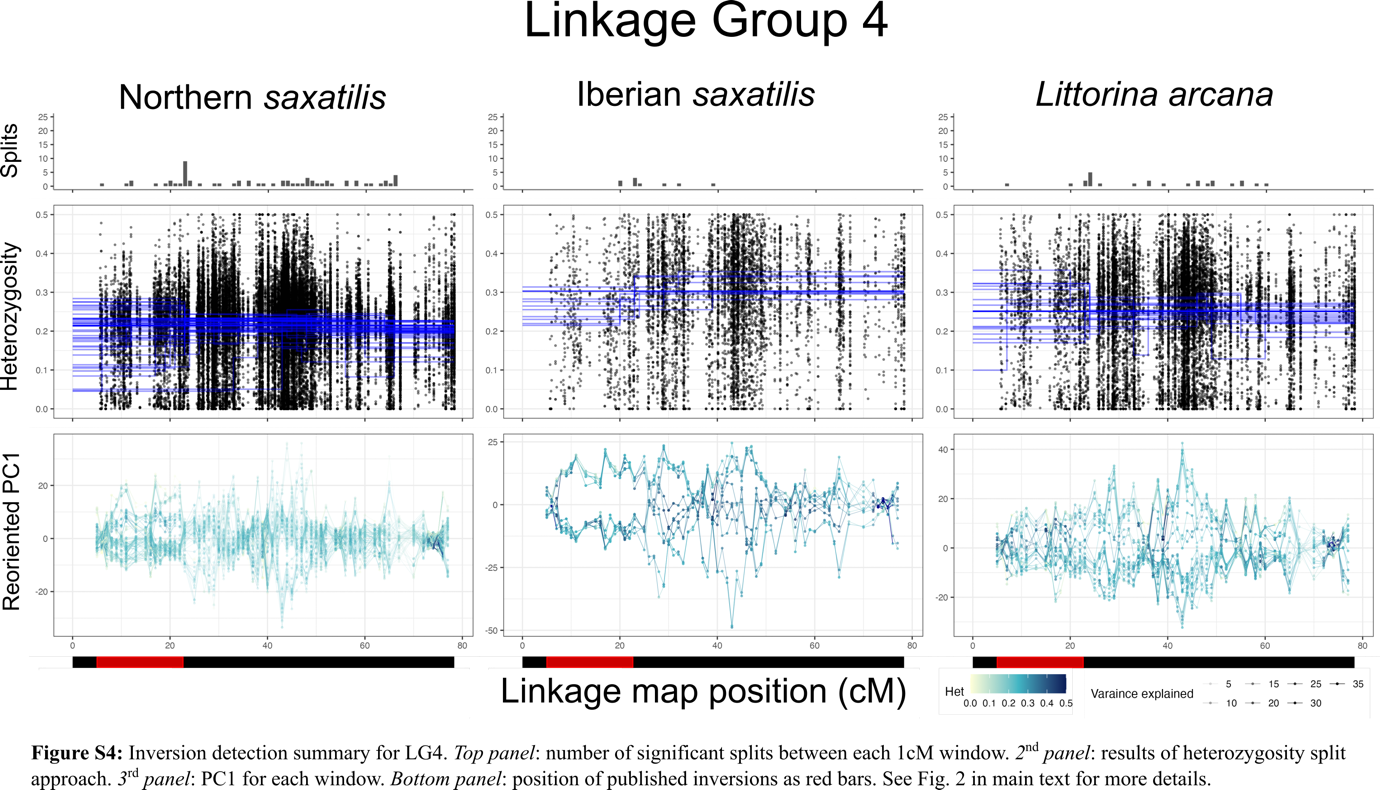
**Figure S4:** Inversion detection summary for LG4. *Top panel:* number of significant splits between each 1cM window. *2^nd^ panel*: results of heterozygosity split approach. *3^rd^ panel*: PC1 for each window. *Bottom panel*: position of published inversions as red bars. See Figure 2 in the main text for more details.

**
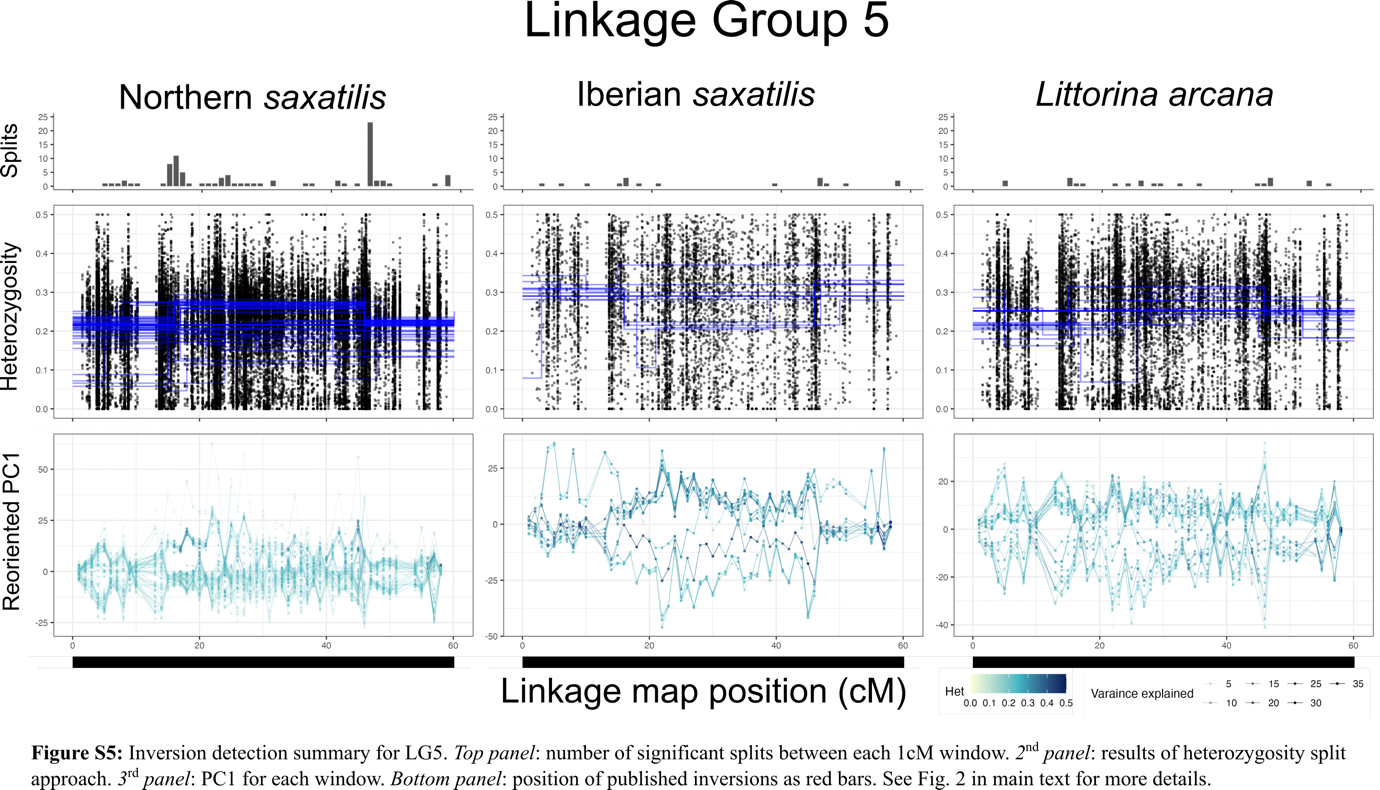
Figure S5:** Inversion detection summary for LG5. *Top panel:* number of significant splits between each 1cM window. *2^nd^ panel*: results of heterozygosity split approach. *3^rd^ panel*: PC1 for each window. *Bottom panel*: position of published inversions as red bars. See Figure 2 in the main text for more details.

**
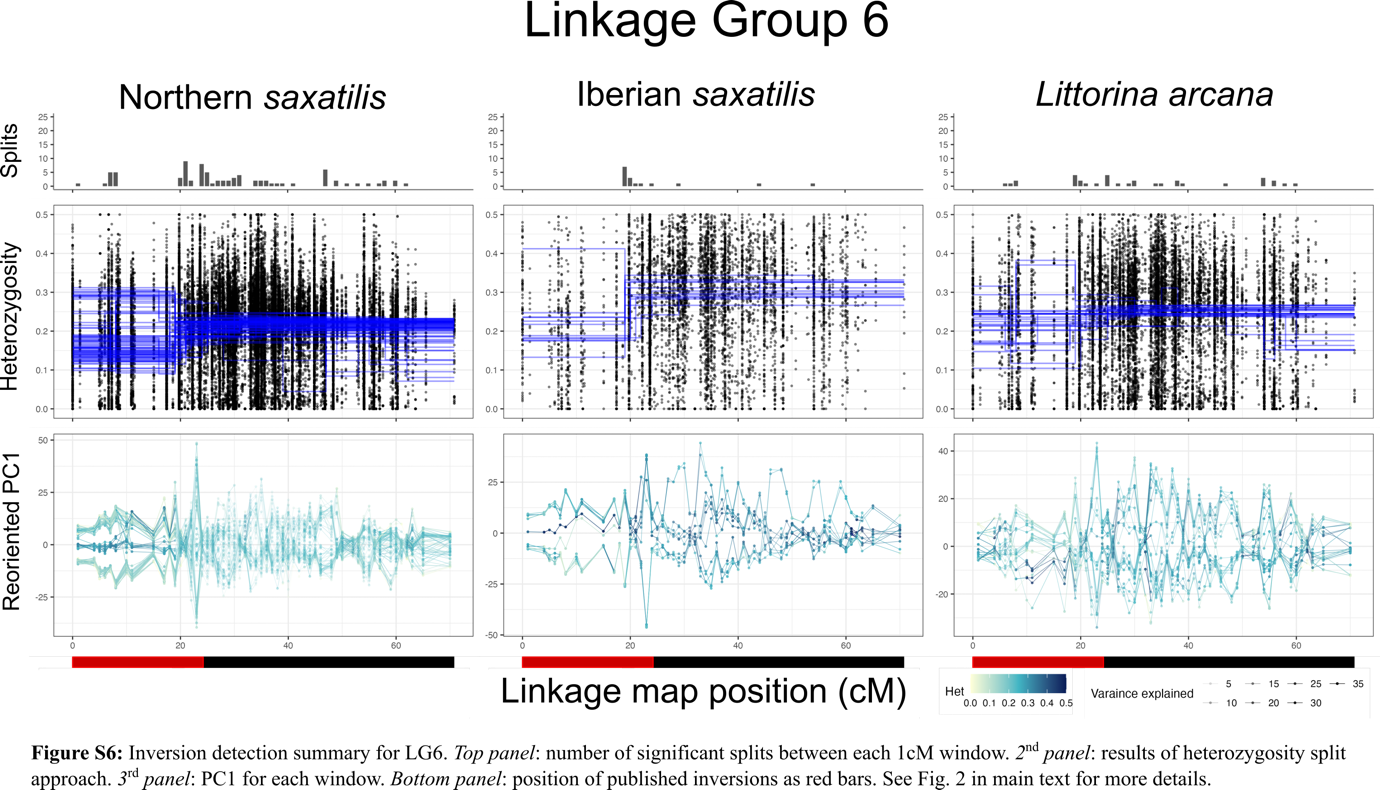
Figure S6:** Inversion detection summary for LG6. *Top panel:* number of significant splits between each 1cM window. *2^nd^ panel*: results of heterozygosity split approach. *3^rd^ panel*: PC1 for each window. *Bottom panel*: position of published inversions as red bars. See Figure 2 in the main text for more details.

**
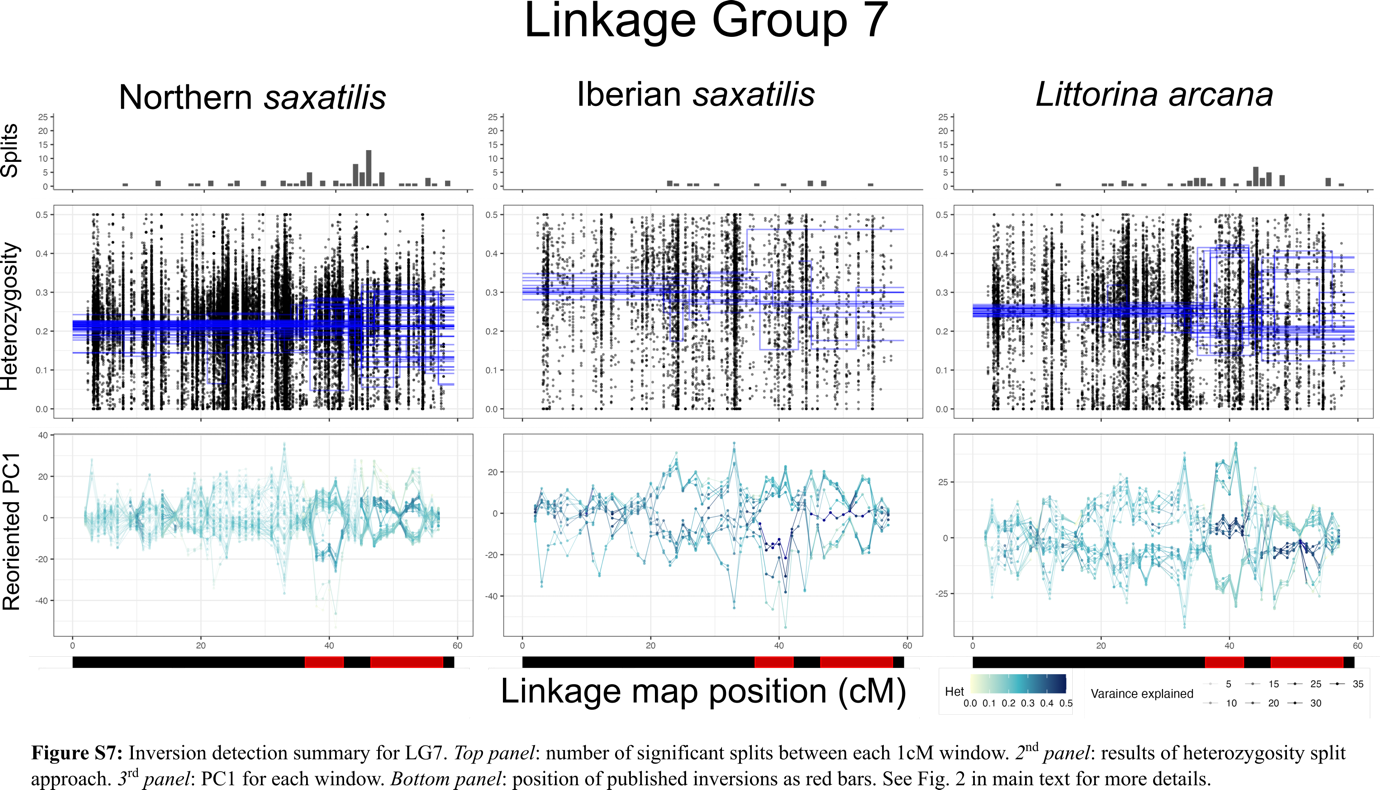
Figure S7:** Inversion detection summary for LG7. *Top panel:* number of significant splits between each 1cM window. *2^nd^ panel*: results of heterozygosity split approach. *3^rd^ panel*: PC1 for each window. *Bottom panel*: position of published inversions as red bars. See Figure 2 in the main text for more details.

**
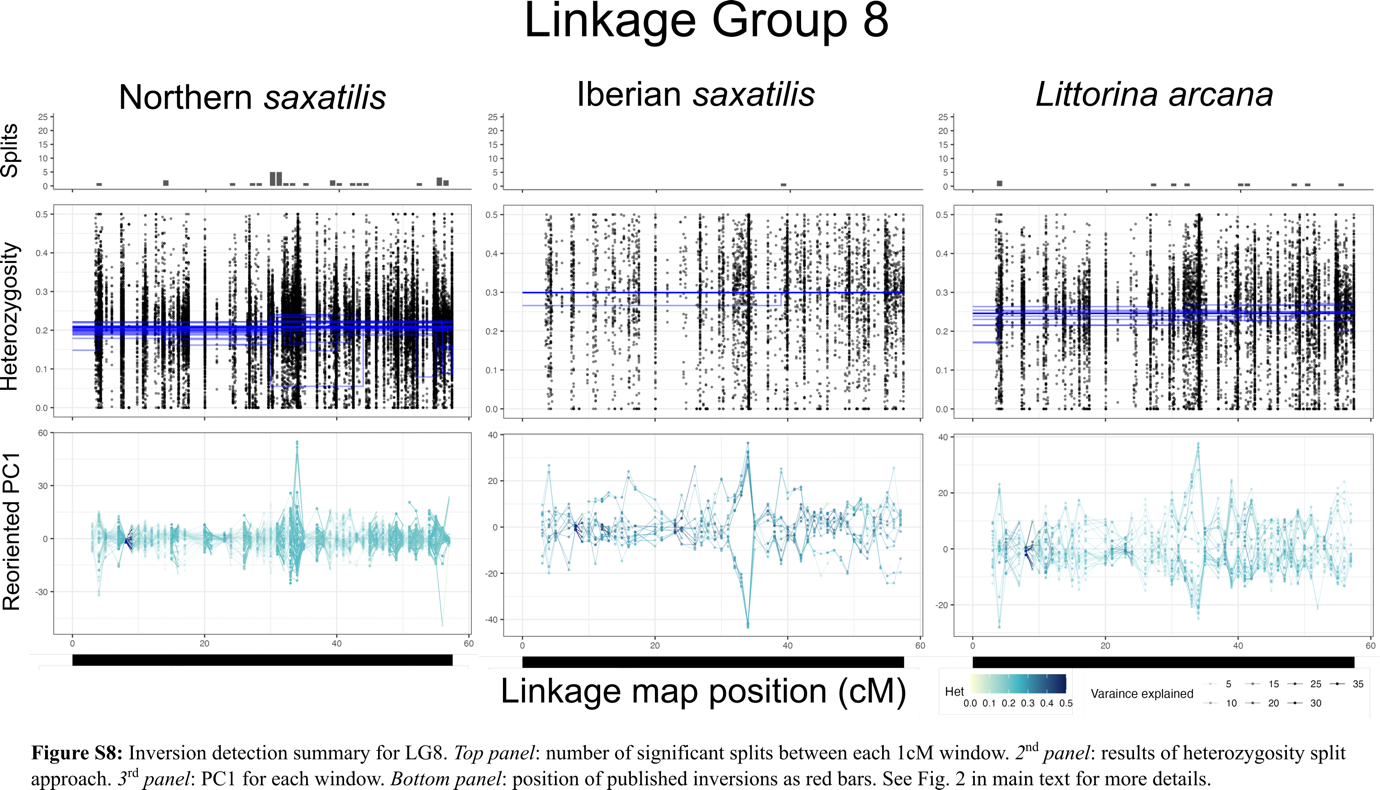
Figure S8:** Inversion detection summary for LG8. *Top panel:* number of significant splits between each 1cM window. *2^nd^ panel*: results of heterozygosity split approach. *3^rd^ panel*: PC1 for each window. *Bottom panel*: position of published inversions as red bars. See Figure 2 in the main text for more details.

**
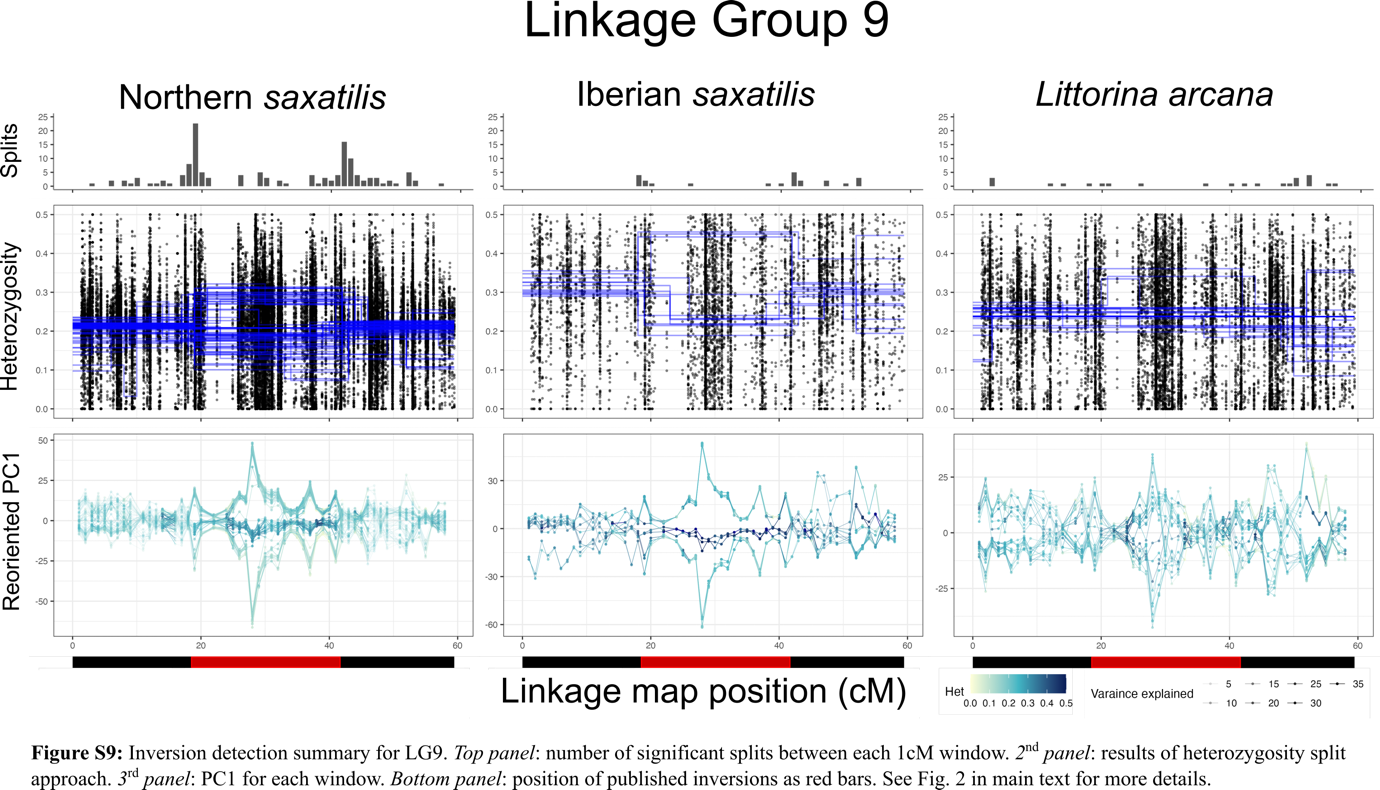
Figure S9:** Inversion detection summary for LG9. *Top panel:* number of significant splits between each 1cM window. *2^nd^ panel*: results of heterozygosity split approach. *3^rd^ panel*: PC1 for each window. *Bottom panel*: position of published inversions as red bars. See Figure 2 in the main text for more details.

**
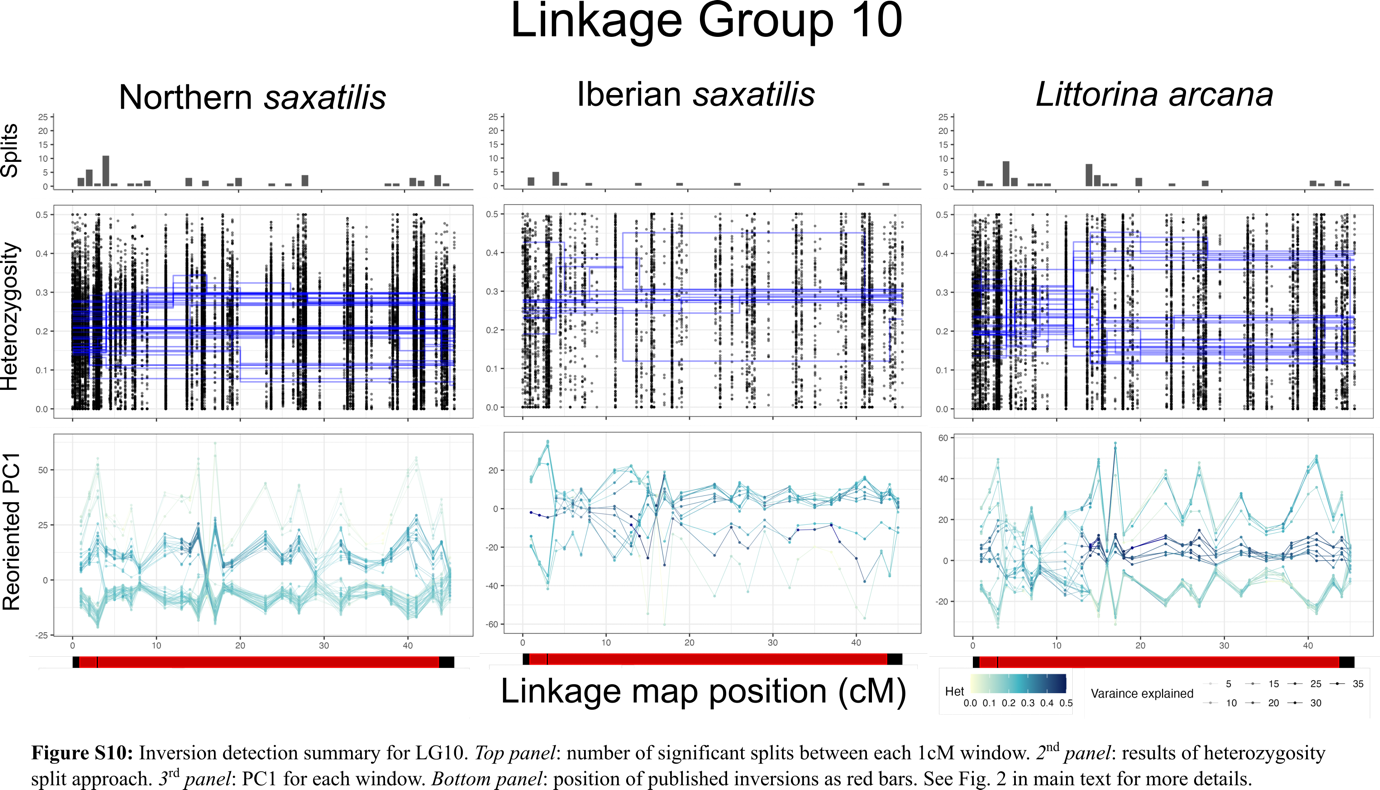
Figure S10:** Inversion detection summary for LG10. *Top panel:* number of significant splits between each 1cM window. *2^nd^ panel*: results of heterozygosity split approach. *3^rd^ panel*: PC1 for each window. *Bottom panel*: position of published inversions as red bars. See Figure 2 in the main text for more details.

**
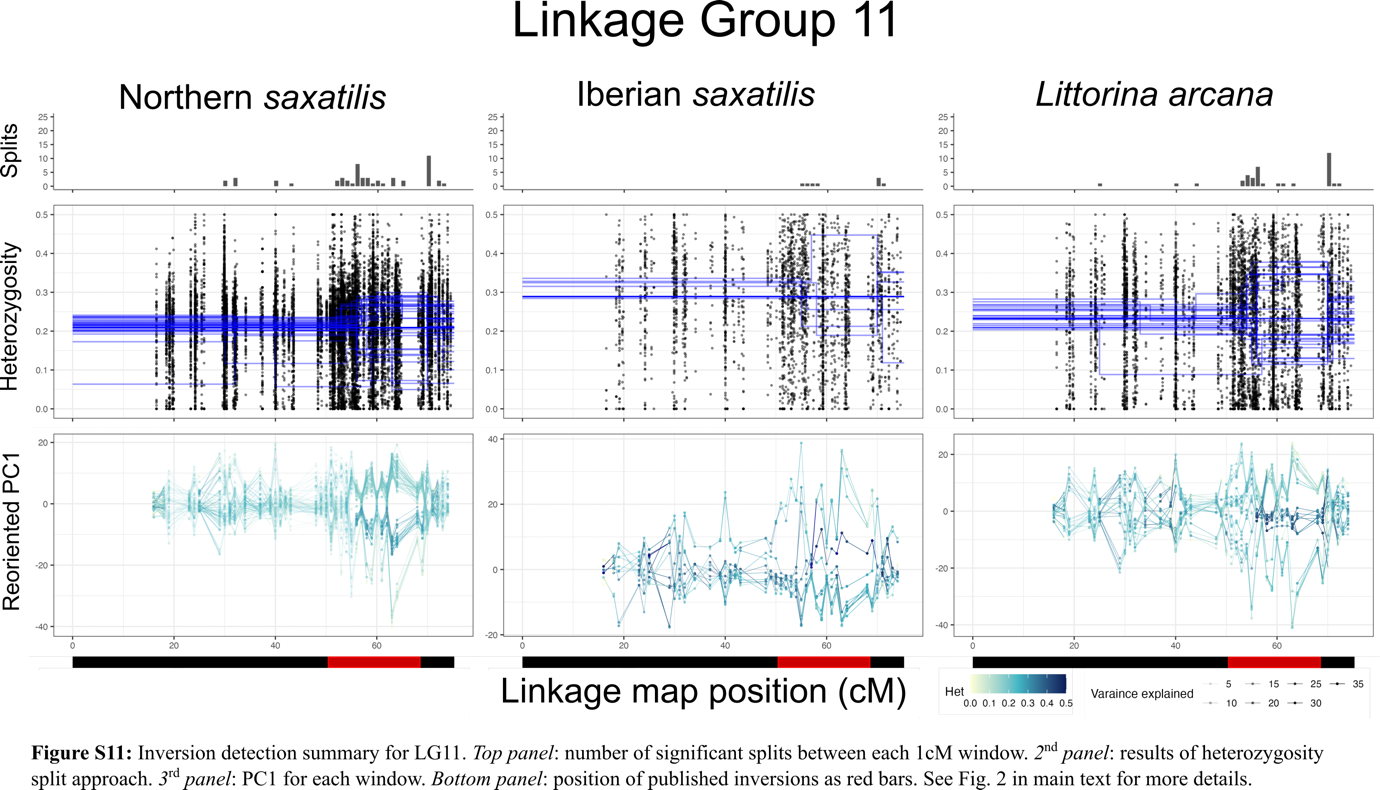
Figure S11:** Inversion detection summary for LG11. *Top panel:* number of significant splits between each 1cM window. *2^nd^ panel*: results of heterozygosity split approach. *3^rd^ panel*: PC1 for each window. *Bottom panel*: position of published inversions as red bars. See Figure 2 in the main text for more details.

**
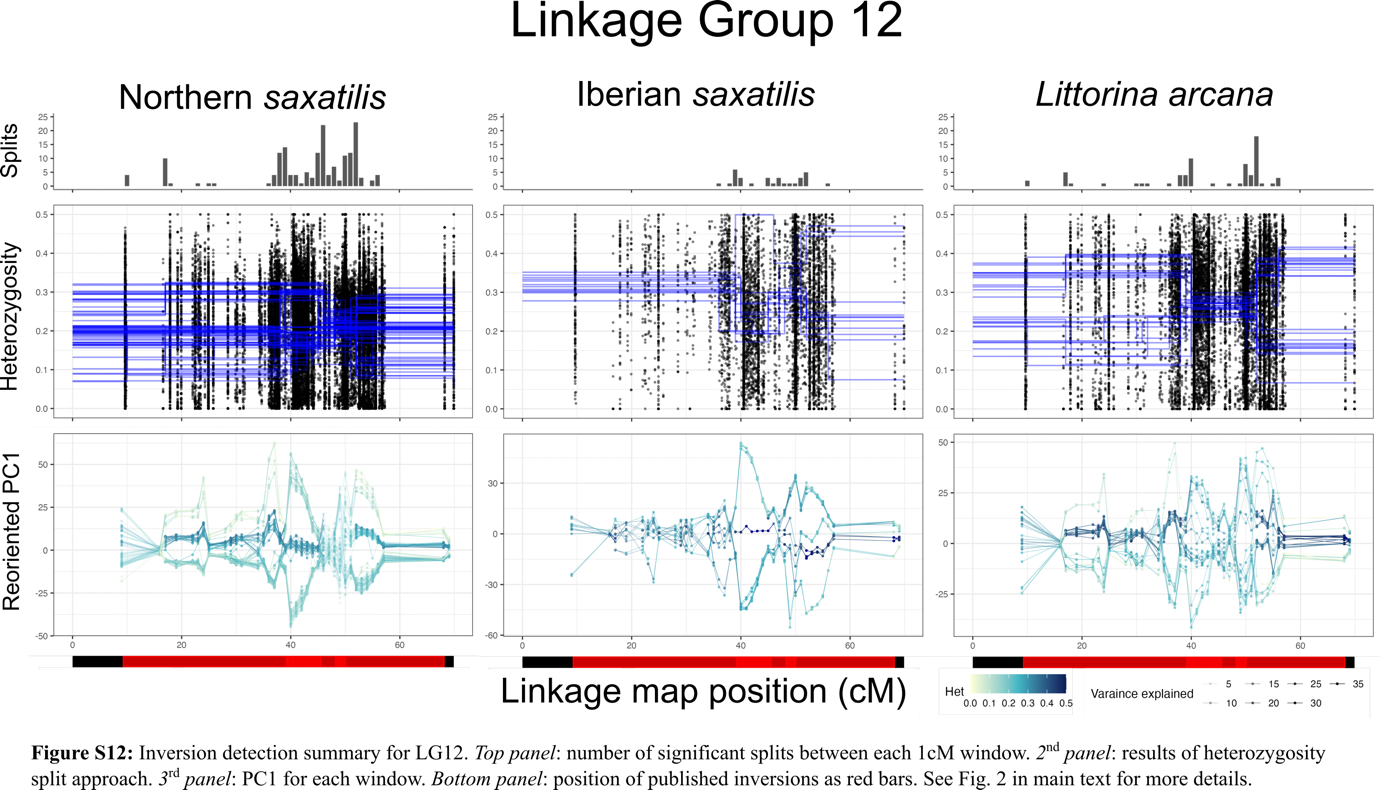
Figure S12:** Inversion detection summary for LG12. *Top panel:* number of significant splits between each 1cM window. *2^nd^ panel*: results of heterozygosity split approach. *3^rd^ panel*: PC1 for each window. *Bottom panel*: position of published inversions as red bars. See Figure 2 in the main text for more details.

**
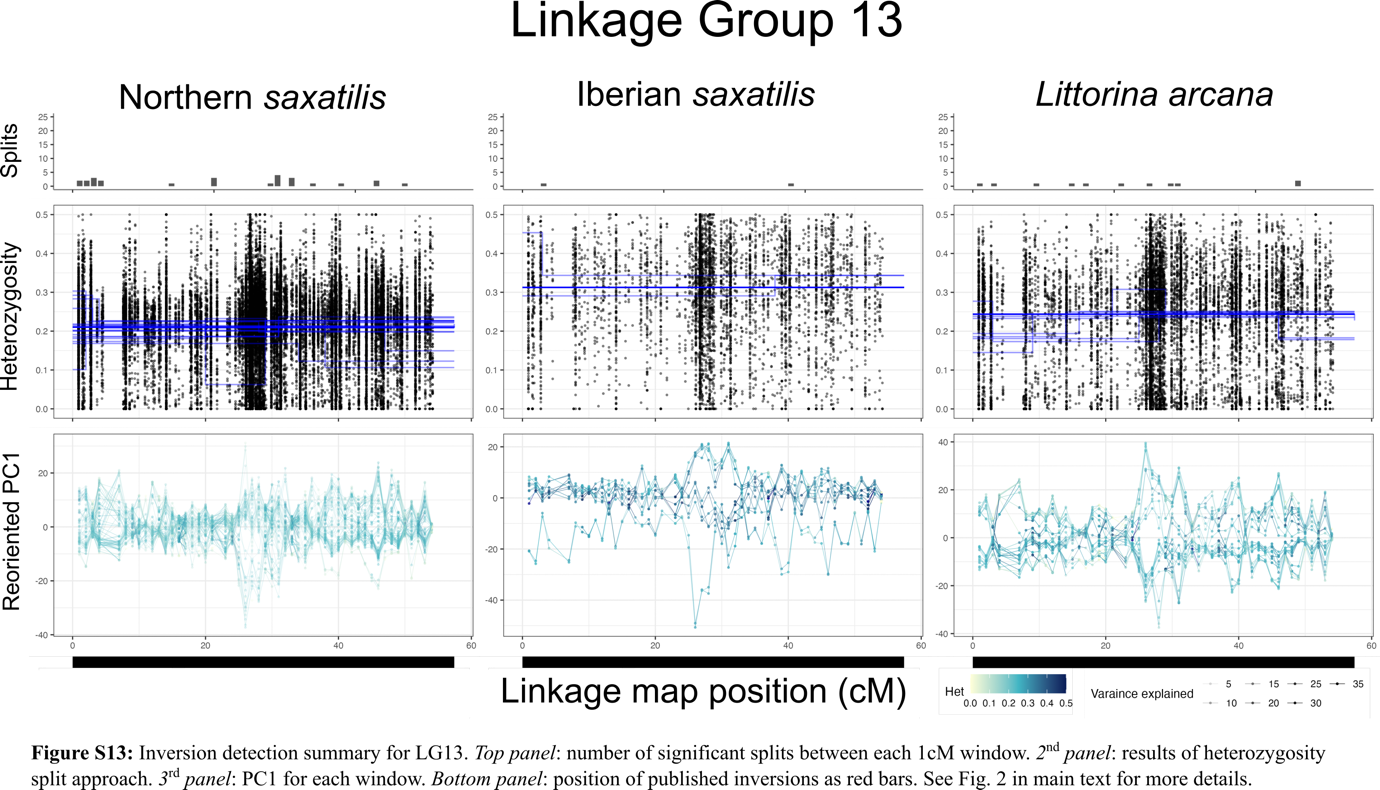
Figure S13:** Inversion detection summary for LG13. *Top panel:* number of significant splits between each 1cM window. *2^nd^ panel*: results of heterozygosity split approach. *3^rd^ panel*: PC1 for each window. *Bottom panel*: position of published inversions as red bars. See Figure 2 in the main text for more details.

**
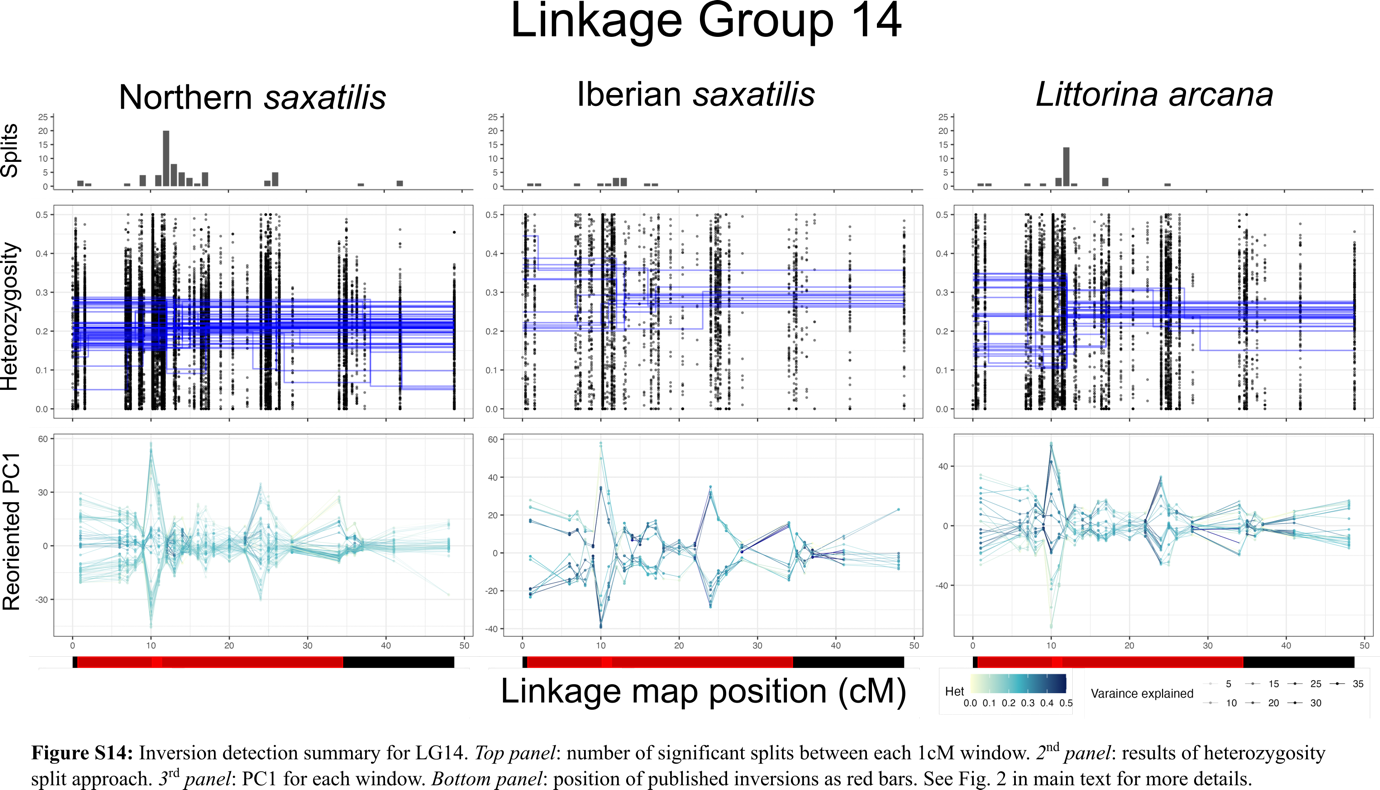
Figure S14:** Inversion detection summary for LG14. *Top panel:* number of significant splits between each 1cM window. *2^nd^ panel*: results of heterozygosity split approach. *3^rd^ panel*: PC1 for each window. *Bottom panel*: position of published inversions as red bars. See Figure 2 in the main text for more details.

**
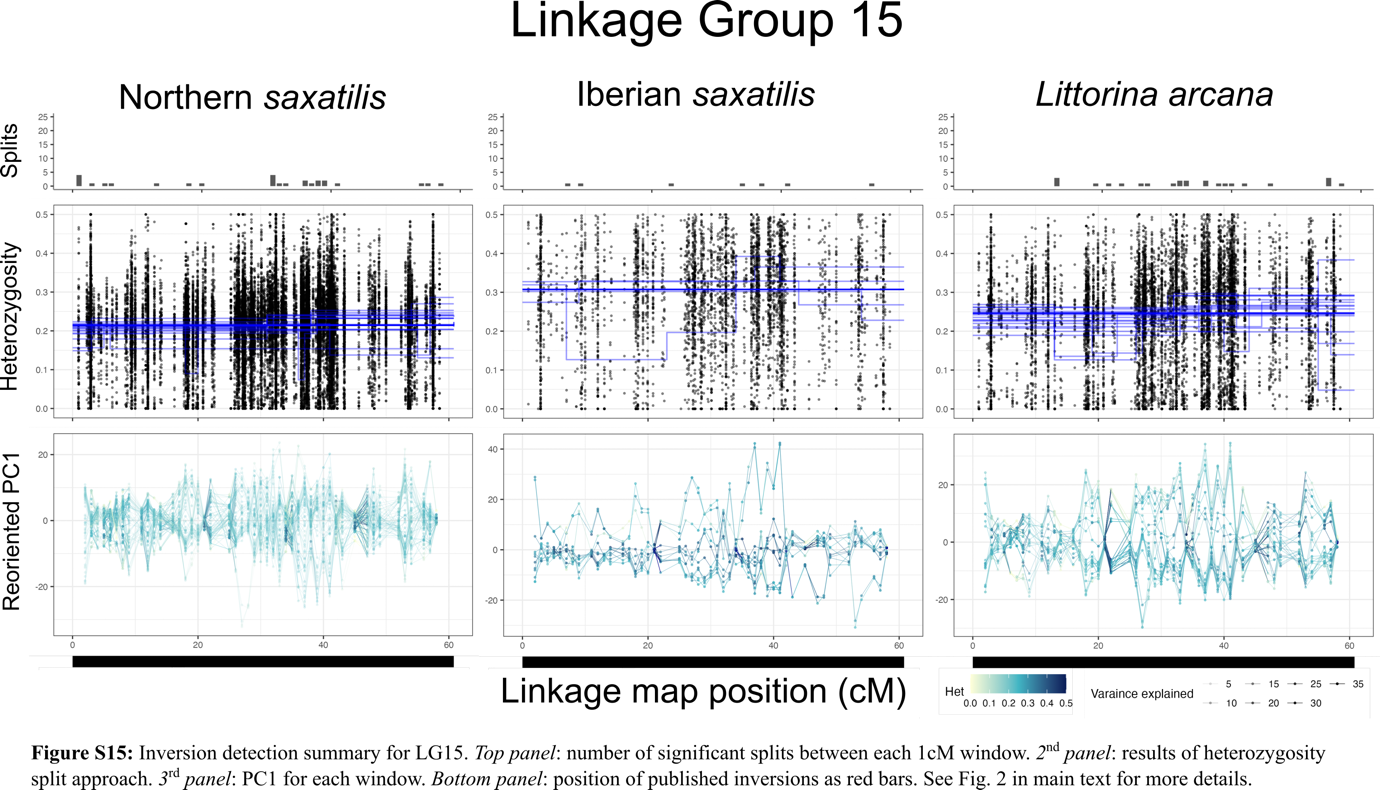
Figure S15:** Inversion detection summary for LG15. *Top panel:* number of significant splits between each 1cM window. *2^nd^ panel*: results of heterozygosity split approach. *3^rd^ panel*: PC1 for each window. *Bottom panel*: position of published inversions as red bars. See Figure 2 in the main text for more details.

**
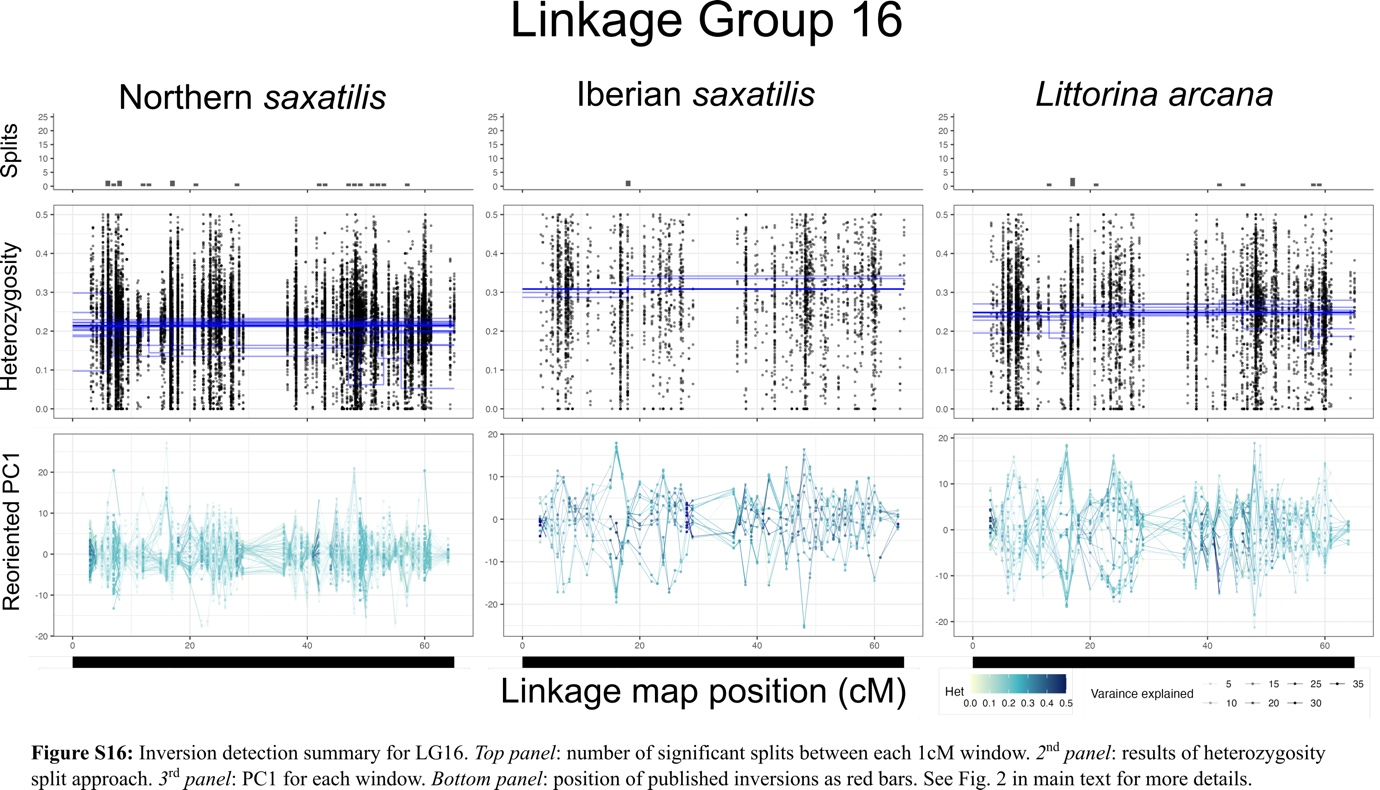
Figure S16:** Inversion detection summary for LG16. *Top panel:* number of significant splits between each 1cM window. *2^nd^ panel*: results of heterozygosity split approach. *3^rd^ panel*: PC1 for each window. *Bottom panel*: position of published inversions as red bars. See Figure 2 in the main text for more details.

**
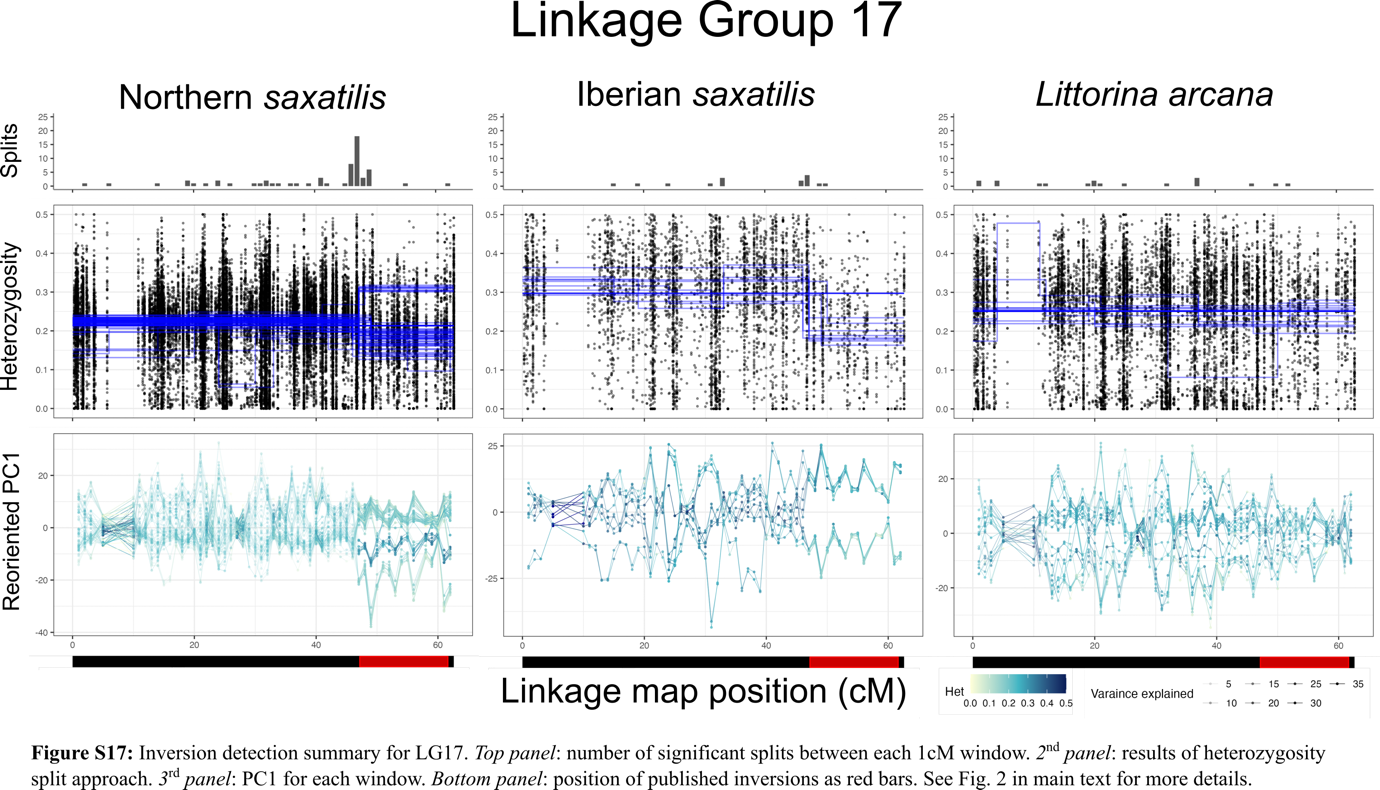
Figure S17:** Inversion detection summary for LG17. *Top panel:* number of significant splits between each 1cM window. *2^nd^ panel*: results of heterozygosity split approach. *3^rd^ panel*: PC1 for each window. *Bottom panel*: position of published inversions as red bars. See Figure 2 in the main text for more details.

**
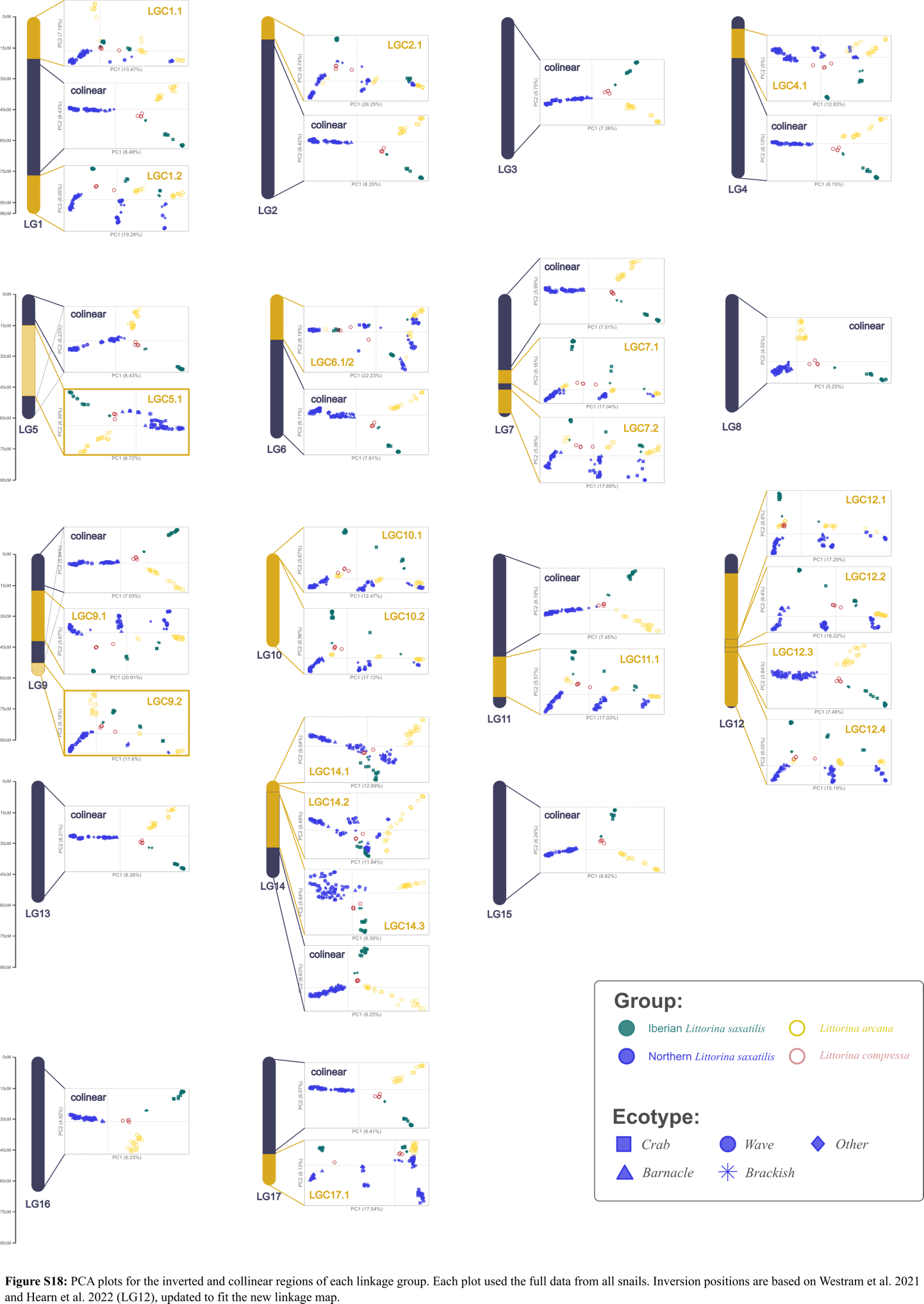
Figure S18:** PCA plots for the inverted and colinear regions of each linkage group. Yellow bordered plots indicate the new putative inversions identified in this study. Each plot uses the full data from all snails. Inversion positions are based on Westram et al., 2021 and Hearn et al., 2022 (LG12), updated to fit the new linkage map.


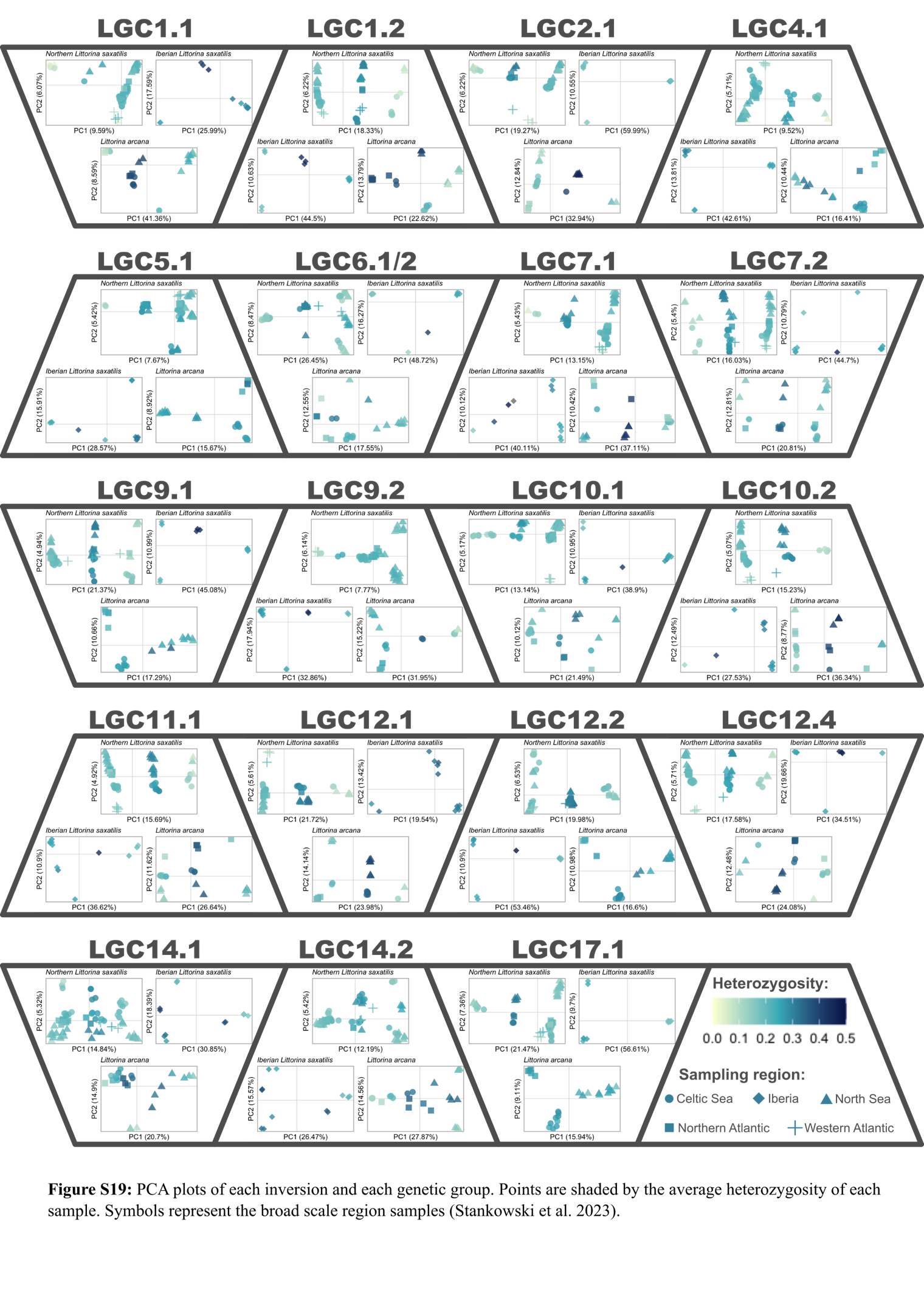


**Figure S19:** PCA plots of each inversion and genetic group. Points are shaded by the average heterozygosity per sample. Symbols represent the broad scale geographic regions identified in a recent phylogeographic study (Stankowski et al., 2023). Western Atlantic is an additional category we defined based on these plots.

**
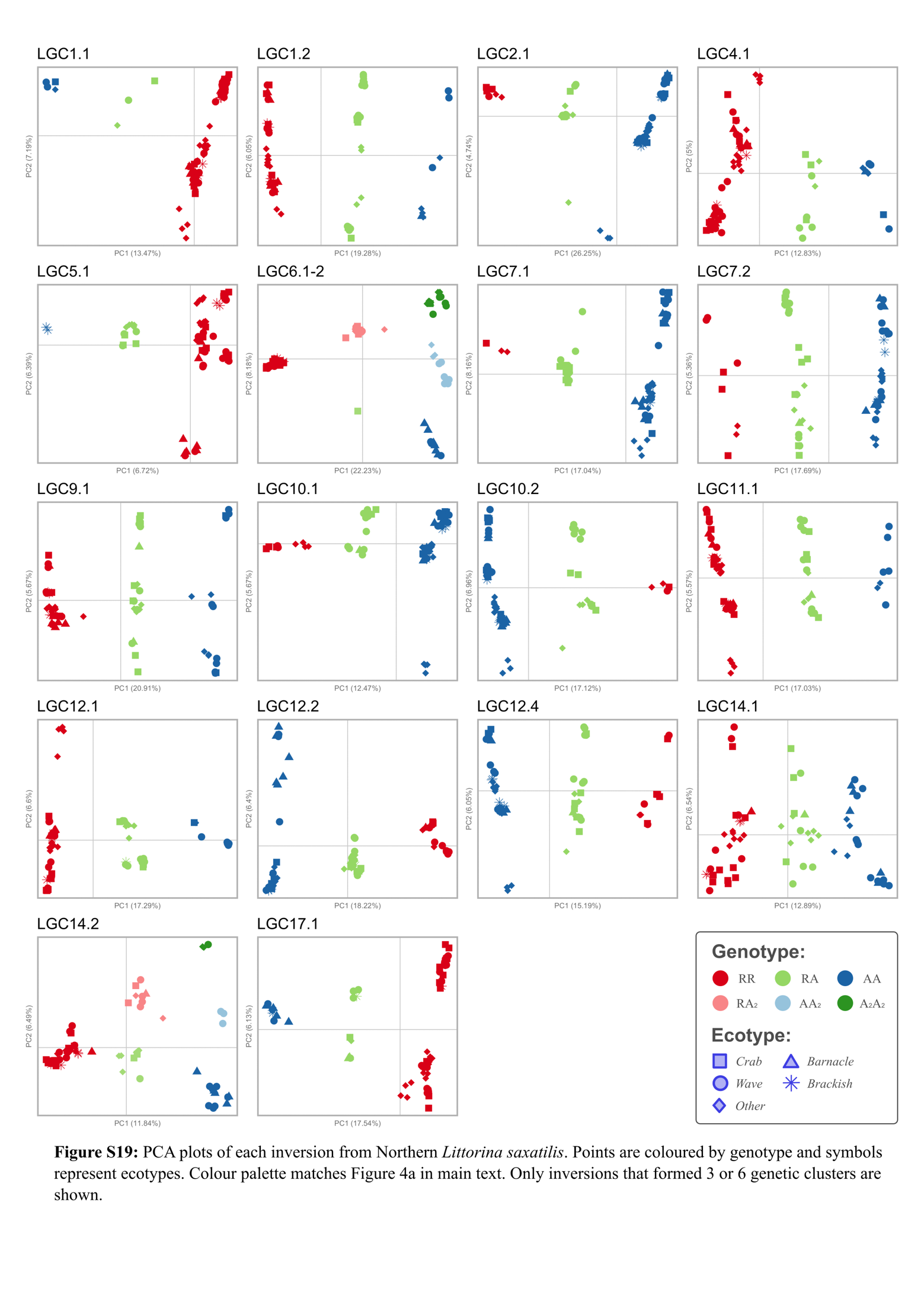
Figure S20:** PCA plots of each inversion from Northern *Littorina saxatilis*. Points are coloured by genotype and symbols represent ecotypes. Colour palette matches Figure 4a in the main text. Only inversions that formed 3 or 6 clusters are shown.

**
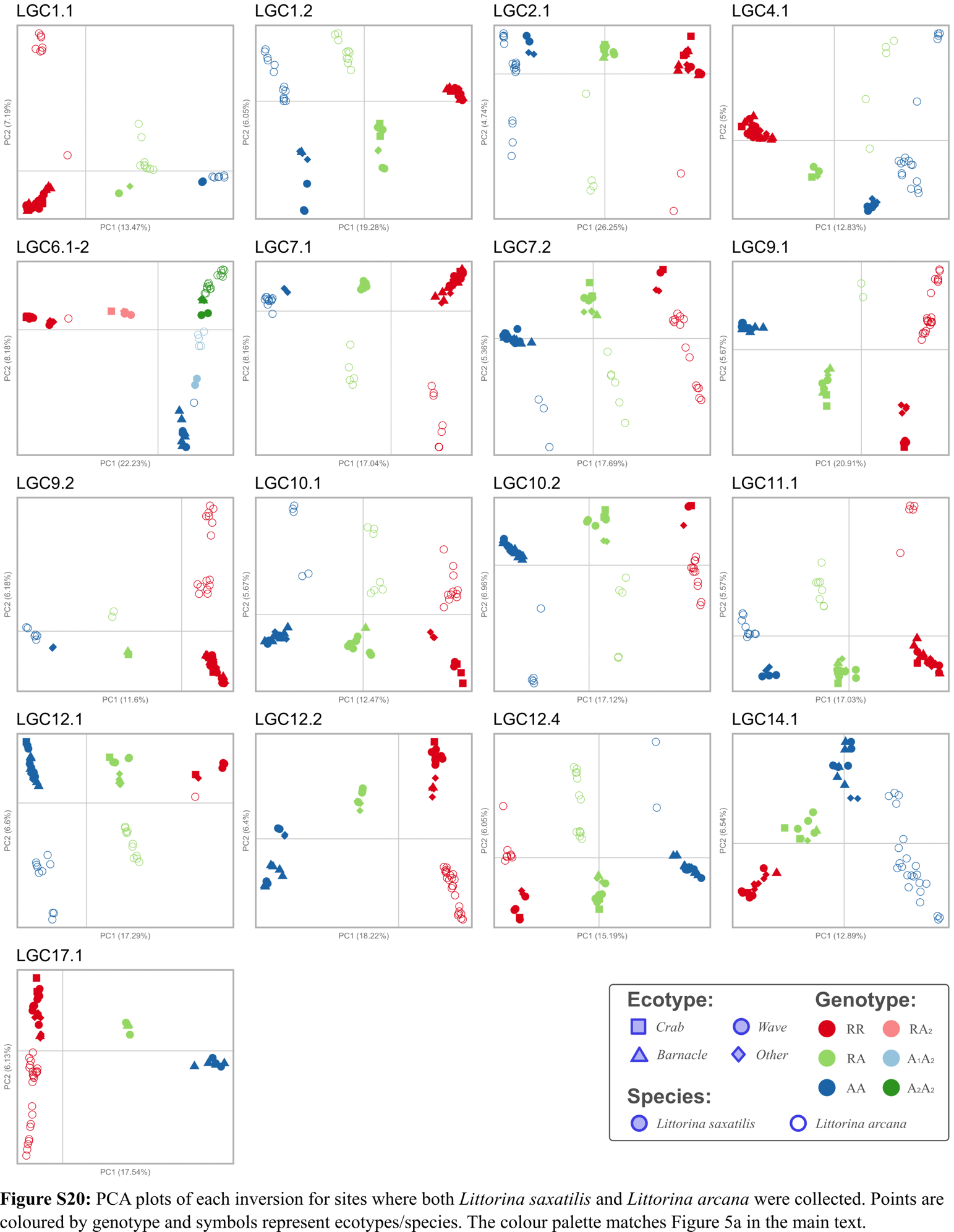
Figure S21:** PCA plots of each inversion for sites where both *Littorina saxatilis* and *Littorina arcana* were collected. Points are coloured by genotype and symbols represent ecotypes/species. The colour palette matches Figure 5a in the main text.

**
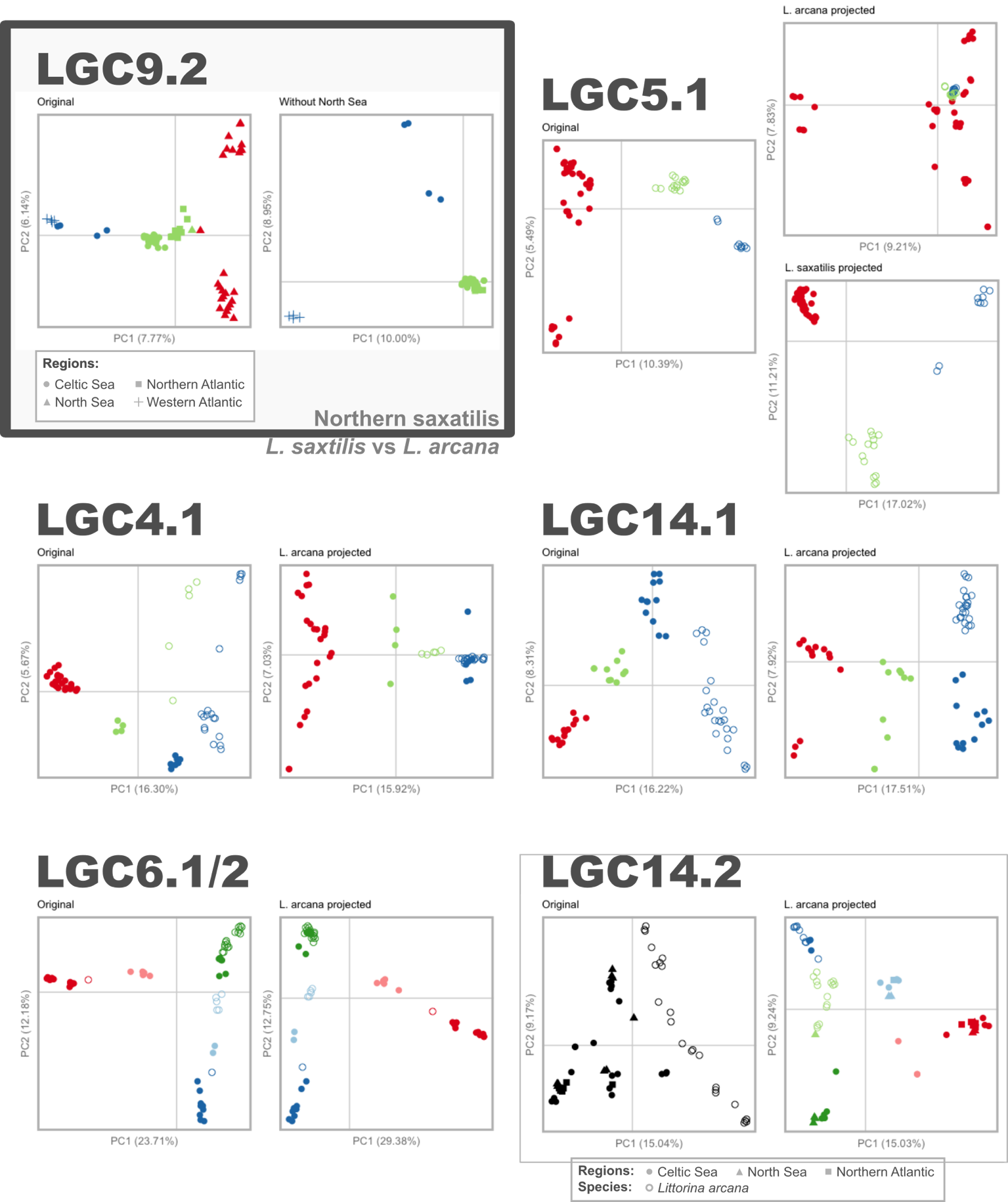
Figure S22:** PCA plots demonstrating the manual adjustments made to genotype some inversions. Adjustments are separated into those that were made using the Northern saxatilis data (grey box), and the *Littorina saxatilis* vs. *L. arcana* data. Titles indicate if the panel is the original PCA result or a projection of one species onto the PCA of the other. In cases when geographic signal was pertinent to the manual adjustment, regions are displayed. Geographic regions were defined from a recent phylogeographic study (Stankowski et al., 2023), with USA samples split into a separate Western Atlantic group. Colours and shapes (except for LGC9.2 and LGC14.2) are the same as Figure 5a in the main text. Sampling regions for LGC9.2 and LGC14.2 are assigned different shapes (see legends), with *L. arcana* represented as hollow circles. The original projection of LGC14.2 is coloured black because genotypes could not be determined for this PCA.
